# Louse-borne relapsing fever—A systematic review and analysis of the literature: Part 2—Mortality, Jarisch–Herxheimer reaction, impact on pregnancy

**DOI:** 10.1371/journal.pntd.0008656

**Published:** 2021-03-11

**Authors:** Pascal Kahlig, Andreas Neumayr, Daniel H. Paris

**Affiliations:** 1 Swiss Tropical and Public Health Institute, Basel, Switzerland; 2 University of Basel, Basel, Switzerland; 3 Department of Public Health and Tropical Medicine, College of Public Health, Medical and Veterinary Sciences, James Cook University, Queensland, Australia; Baylor College of Medicine, UNITED STATES

## Abstract

Louse-borne relapsing fever (LBRF) is a classical epidemic disease, which in the past was associated with war, famine, poverty, forced migration, and crowding under poor hygienic conditions around the world. The disease’s causative pathogen, the spirochete bacterium *Borrelia recurrentis*, is confined to humans and transmitted by a single vector, the human body louse *Pediculus humanus corporis*. Since the disease was at its peak before the days of modern medicine, many of its aspects have never been formally studied and to date remain incompletely understood. In order to shed light on some of these aspects, we have systematically reviewed the accessible literature on LBRF since the recognition of its mode of transmission in 1907, and summarized the existing data on mortality, Jarisch–Herxheimer reaction (JHR), and impact on pregnancy.

Publications were identified by using a predefined search strategy of electronic databases and a subsequent review of the reference lists of the obtained publications. All publications reporting patients with a confirmed diagnosis of LBRF published in English, French, German, and Spanish since 1907 were included. Data extraction followed a predefined protocol and included a grading system to judge the certainty of the diagnosis of reported cases.

The high mortality rates often found in literature are confined to extreme scenarios. The case fatality rate (CFR) of untreated cases is on average significantly lower than frequently assumed. In recent years, a rise in the overall CFRs is documented, for which reasons remain unknown.

Lacking standardized criteria, a clear diagnostic threshold defining antibiotic treatment-induced JHR does not exist. This explains the wide range of occurrence rates found in literature. Pre-antibiotic era data suggest the existence of a JHR-like reaction also in cases treated with arsenicals and even in untreated cases.

LBRF-related adverse outcomes are observed in 3 out of 4 pregnancies.

## Author summary

Louse-borne relapsing fever (LBRF) is an ancient epidemic disease, with descriptions dating back to Hippocrates’ times. Linked to war, famine, poverty, forced migration, and crowding under poor hygienic conditions, the disease has accompanied humankind throughout history and, until 100 years ago, the disease was well known among physicians in Europe and North America. Since then, the disease has fallen into oblivion, and reports of the disease are largely confined to Ethiopia. However, the disease has recently resurfaced when it was recognized as a cause of fever in East African migrants (originating from Somalia, Eritrea, and Ethiopia) arriving to Europe. Since the disease was at its peak before the days of modern medicine, many aspects of the disease have never been formally studied and, to date, remain incompletely understood. In order to shed light on some of these aspects, we have reviewed and analysed the accessible literature on LBRF since the recognition of its mode of transmission in 1907, and compiled this two-part review focusing on epidemiology and diagnostic aspects (part 1) and mortality, JHR, and impact on pregnancy (part 2). We deliberately did not include an analysis on the antimicrobial treatment of LBRF, since Guerrier and Doherty have published a comprehensive meta-analysis on this aspect in 2011.

## Introduction

Relapsing fevers are potentially fatal ectoparasite-borne diseases which are caused by spirochetes of the genus *Borrelia* and characterized by recurring episodes of fever [1].

Relapsing fevers are classified according to their transmitting vector which include tick-borne relapsing fever (TBRF) and louse-borne relapsing fever (LBRF). While several *Borrelia* spp. have been identified to cause TBRF, *Borrelia recurrentis* is the only species known to cause LBRF [2]. The incubation period of LBRF ranges from 4 to 18 (average 7) days. The attack starts abruptly with a fever that increases to nearly 40°C in a few days, accompanied by rigors. Early symptoms include headache, dizziness, generalized aches and pains (affecting especially the lower back, knees, and elbows), anorexia, nausea, vomiting, and diarrhea. Upper abdominal pain, cough, and epistaxis develop later [3]. A petechial or ecchymotic rash, particularly involving the trunk, is seen in 2% to 8% of patients, and 7% to >70% of patients develop jaundice [4]. Subconjunctival hemorrhages and epistaxis are common (25%), hemoptysis, gastrointestinal bleeding, and retinal hemorrhages less so [3]. In severe cases, neurological involvement, myocarditis, acute respiratory distress syndrome, hepatic failure, spleen rupture, disseminated intravascular coagulation leading to intracranial, massive gastrointestinal, pulmonary or peripartum hemorrhage may occur [3]. Case fatalities between 30% and 80% [4–12] have been reported in untreated patients during major epidemics, but with antibiotic treatment mortality is reduced to 2% to 5% [13]. Untreated attacks resolve by crisis after 4 to 10 (average 5) days. This is followed by an afebrile remission of 5 to 9 days and succeeded by up to 5 relapses of diminishing severity, during which there may be epistaxis, but no petechial rashes [3]. The crisis which abruptly terminates untreated attacks is a consequence of specific bactericidal antibodies lysing spirochetes in the blood. The relapse phenomenon can be attributed to the antigenic variation of the bacterium’s outer membrane lipoproteins (vmp) [14]. Once antibodies have been generated against a specific vmp variant, a new vmp variant is expressed by the *Borrelia*. After the removal of the majority population through antibodies, the minority variant population expands until antibodies are also generated against the new vmp variant. Also linked to vmp is the phenomenon of Jarisch–Herxheimer reaction (JHR) [15]. Named after the researchers who first described the reaction in patients with syphilis [16–18], the JHR has been described to occur after the elimination of spirochetes in patients with syphilis, leptospirosis, Lyme disease, and relapsing fever [18]. Around 1970, several authors had studied this reaction in LBRF patients after treatment with antibiotics [4,18–21]. The observations resembled the 4 phases described for fever induced by endotoxin [22]. JHR occurs about 45 minutes to 2 hours after administration of antibiotic treatment and is characterized by restlessness followed by a chill phase of acute and intense rigors lasting 10 to 30 minutes. During this chill phase, body temperature, pulse, and respiratory rates rise steeply, and associated delirium (sometimes leading to dangerous behavior) as well as potentially fatal hyperpyrexia may occur. The flush phase following the chill phase is characterized by a systemic vasodilatation with a fall in blood pressure (possibly leading to collapse and intractable hypotension). This is accompanied by profuse sweating and a slowly declining body temperature, threatening the patient for several hours. The prodromal phase, the chill phase, the flush phase, and the phase of defervescence have been described in detail by several authors [4,20]. Both the spontaneous crisis that terminates untreated attacks and the JHR induced by antibiotic treatment show pathophysiological features of a classic endotoxin reaction mediated by proinflammatory cytokines (tumor necrosis factor α [TNF-α], interleukin 6 (IL-6), IL-8) [18]. A recent review has addressed the current understanding of the pathogenesis of JHR, which seems to be of multifactorial genesis and is not fully understood yet [18]. A JHR occurs in up to 80% to 90% of treated patients [13], and symptoms usually resolve in a few hours. Even though JHR is rarely fatal, it does enhance the risk for complications and a fatal outcome (liver and renal function impairment, acute respiratory distress syndrome, myocardial injury, hypotensive shock, seizures, strokes, induction of uterine contractions in pregnancy) [13,18]. Besides supportive treatment, administration of antibodies against TNF-α have been proven effective in the management of the JHR [23–25].

A variety of antibiotic drugs, including tetracyclines, erythromycin, chloramphenicol, and penicillin, are effective as single-dose treatment (given orally or parenterally) in LBRF [13]. A meta-analysis of antibiotic treatment of LBRF published by Guerrier and Doherty in 2011 found no significant difference between tetracycline and penicillin with regard to mortality rate. Tetracycline use was found to be associated with faster resolution of fever and a lower risk of relapse compared to penicillin treatment. However, tetracycline use appears to be associated with a higher risk for JHR compared to penicillin treatment [26].

In a study on TBRF in pregnant women conducted in Rwanda, the risk of pregnancy loss was 33% and that of perinatal mortality 15% [27]. In another study on TBRF in pregnant women in Tanzania, the risk of pregnancy loss was 30% and that of perinatal mortality 15% [28]. In contrast to TBRF, data on the impact of LBRF on pregnancy is scarce. A publication on LBRF published in 1970 states: "*Abortion or miscarriage is the usual fate of pregnancy*. *That we had one live premature birth and three uninterrupted pregnancies is probably due to a standard of care not normally available in an epidemic*. *That congenital infection and abortion are the rule is clear from El Ramly’s post-mortem studies (1946)*" [4].

With this systematic review, we aim to summarize the available data and address the following main review questions: What is the mortality of untreated and treated LBRF? What are the reported frequencies of the JHR in LBRF? What is the impact of LBRF on pregnancy?

## Methods

A systematic review protocol established for this review is available in the Supporting information section (S1 Text). The electronic databases Biosis, CINAHL, Cochrane Library, Current Contents Connect, Elsevier, EMBASE ovid, Ovid MEDLINE, PMC, PUBMED, SCOPUS, and Web of Science were searched on 04/Oct/2017 using the search term: ((Relapsing AND fever AND (Louse OR Lice OR (Pediculus AND humanus))) OR (Borrelia AND recurrentis) OR LBRF). A second and third search, using the same search term on the same databases, was conducted on 07/Aug/2018 and 17/Jun/2019, respectively. After checking for and removing duplicates (using Endnote software and manually [29]), publications were prescreened by checking titles and abstracts if they concerned patient(s) with the diagnosis of LBRF. Publications not reporting patient(s) with the diagnosis of LBRF were excluded. The remaining publications were then assessed in full text for eligibility according to the inclusion criteria: reporting conclusively diagnosed case(s) of LBRF and published after 1907 (the year of the discovery of the disease’s transmission) and published in English, French, German, or Spanish. Publications not fulfilling these inclusion criteria were excluded. Publications that could neither be retrieved through their respective journals, nor by contacting libraries, or after contacting the authors, were classified as “not retrievable” and excluded. Additional relevant publications identified when reading the full-text articles or checking their reference lists were reviewed and included if they fulfilled the inclusion criteria (“snowball” search strategy). Finally, a subsearch was conducted on (i) outcome, (ii) JHR, and (iii) impact on pregnancies. Inclusion criteria for (i) outcome were: available conclusive data on number of patients treated/untreated plus treatment plus mortality. Inclusion criteria for (ii) JHR were: available conclusive data on number of patients treated/untreated plus number of patients with a JHR/JHR rate plus reaction after treatment described as JHR. In order to investigate a potential influence of the study objectives/endpoints on the reported incidence rates of JHR, we conducted a subgroup analysis. Thus, the 42 included studies were divided into 2 groups, one primarily focusing on JHR and the other primarily focusing on other aspects of LBRF. Inclusion criteria for (iii) impact on pregnancies were: available conclusive data on the number of cases plus pregnancy outcome.

A data extraction sheet for screening and selecting eligible publications was developed and is available in the Supporting information section (S2 Text). The following data were extracted from eligible publications using a standardized excel spreadsheet: patient characteristics (number of patients, age, gender, origin, occupation, social status, and way and duration of migration), diagnostic method (microscopy and molecular method), symptoms and signs (fever, chills, myalgia, headache, hepatomegaly, splenomegaly, signs of hemorrhage, and others), treatment (number of treated and untreated patients, drug, dosage, and duration and route of administration), and outcome (JHR, abortion/stillbirth, premature delivery, and mortality).

To minimize bias, the same reviewer conducted a second full data extraction ≥1 month after the first extraction. Discrepant results and unclear cases were resolved by consulting a second reviewer. In order to consider the probability of a correct diagnosis of LBRF, all reviewed cases were graded according to the used diagnostic method and respectively classified (Table 1).

**Table 1:** Diagnostic grading system to judge the certainty of the correct diagnosis of LBRF.

Diagnostic method	Grade of diagnostic certainty	Case classification	Comment
PCR-based method	A	Confirmed diagnosis	Highest level of evidence for correct diagnosis
Microscopy	B	Microscopy diagnosis	Second highest level of evidence for correct diagnosis; microscopic identification of spirochetes during LBRF epidemics or in countries with current endemic foci leaves little doubt of the certainty of the diagnosis and may be regarded with an almost equal level of certainty as grade A
Paired serology	C	Indirect evidence	Intermediate level of evidence for correct diagnosis due to limited sensitivity and specificity of the method; paired serology, demonstrating seroconversion or increment of titer, is considered superior to single titer serology
Single titer serology	D	Indirect evidence	See comment under C above
Clinical diagnosis	E	Clinical diagnosis	Lowest level of evidence for correct diagnosis

PCR, polymerase chain reaction.

Note: Animal inoculation, historically used as supportive diagnostic method in LBRF research, was not considered a means of conclusive diagnosis and was thus not included in the evaluation.

The review followed the Preferred Reporting Items for Systematic Reviews and Meta-Analyses (PRISMA) statement (S1 Checklist).

## Results

**Fig 1:**
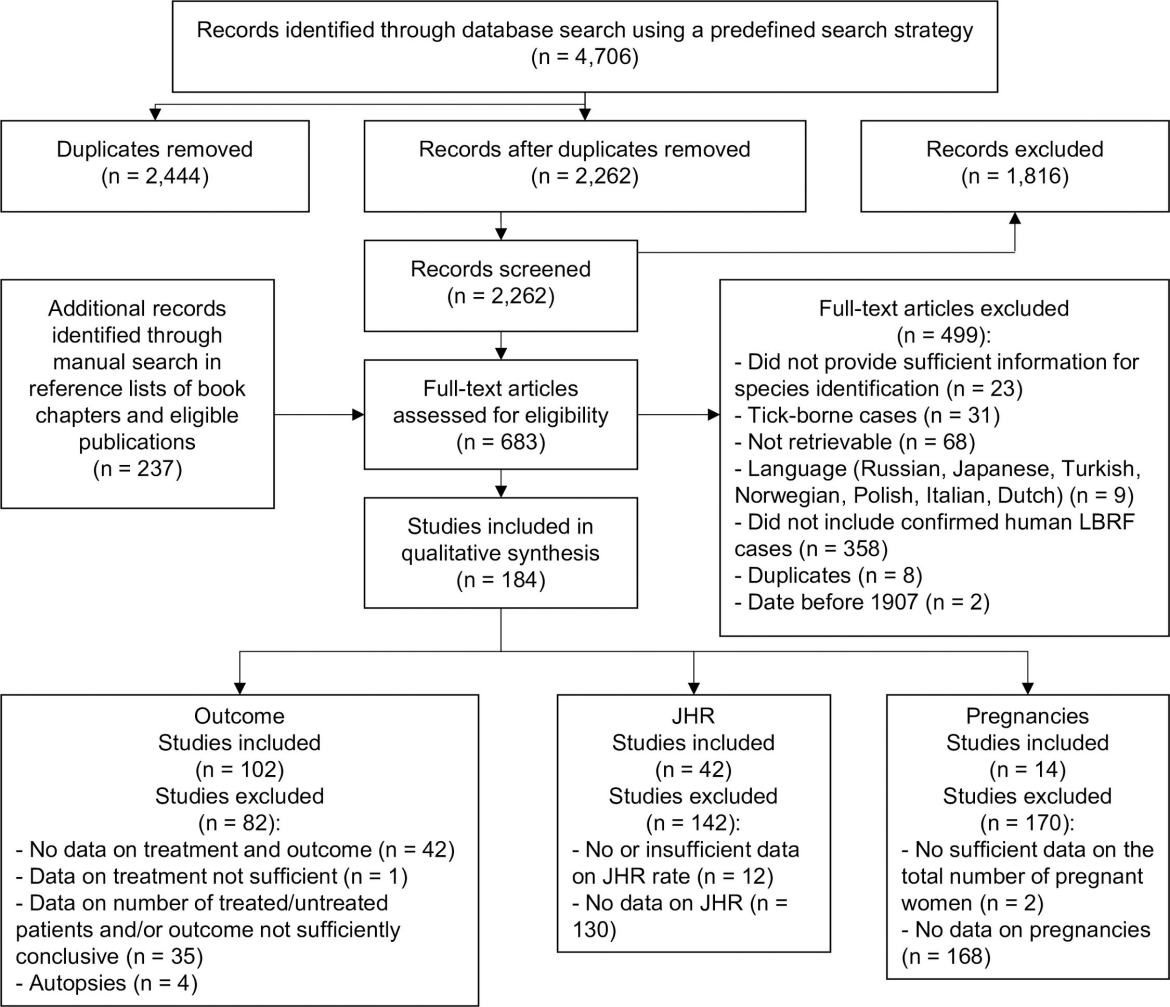
Flow diagram of search and selection of eligible publications.

Our search strategy identified 4,943 publications of which 184 proved eligible for being included and analysed (Fig 1; S1 Fig; S1 Data). Lists of included and excluded publications are available in the Supporting information section (S3–S6 Text).

### Mortality

After full-text assessment of the eligible 184 publications, 102 proved eligible after applying subsearch inclusion criteria for "outcome."

The identified treated cases (*n* = 5,969) were divided into 2 groups according to the treatments used: treated with arsenicals (*n* = 2,338) and treated with antibiotics (*n* = 3,631). The group treated with antibiotics was not divided further, since a recent systematic review has already compared the antibiotic treatments of LBRF [26]. Identified studies with data on untreated cases are listed in Table 2.

**Table 2:** Details of all identified studies that included untreated cases, in chronological order and according to diagnostic grading.

First Author	Year of pub.	Country	Grade of diagnostic certainty	Number of patients	Number of fatal cases	Case fatality rate in %	Ref.
**Duchamp**	1917	Serbia	B	68	0	0	[30]
**Porot**	1917	Algeria	E^†^	2	0	0	[31]
**Margolis**	1919	Poland	E^‡^	267	0	0	[32]
**Del Prado**	1920	Peru	B	2	0	0	[33]
**Fry**	1920	Iran	B	11	1	9.1	[34]
**Jouveau-Dubreuil**	1920	China	B	89	12	13.5	[35]
**Sinton**	1921	Iran	B	21	0	0	[36]
**Sergent**	1922	Algeria	E^‡^	145	0	0	[37]
**McCulloch**	1925	Nigeria	B	68	32	47.1	[38]
**McCulloch**^**§**^	1925	Nigeria	B	16	2	12.5	[38]
**Chu**	1931	China	B	19	0	0	[39]
**Chung**	1936	China	B	1	0	0	[40]
**Chung**	1939	China	B	56	8	14.3	[41]
**Wolman**	1944	Ethiopia	B	103	5	4.9	[42]
**Wolman**	1945	Egypt	B	80	1	1.3	[43]
**Ingraham**	1946	Egypt	B	53	0	0	[44]
**Perine**	1983	Ethiopia	B	6	0	0	[45]

Pub, publication; Ref, reference.

^†^ Report of treated cases, including 2 untreated cases which were diagnosed retrospectively based on clinical presentation.

^‡^ The use of microscopy is mentioned in the descriptions of a few cases. However, whether microscopy was systematically used or only in some cases is unclear. Thus, the studies were conservatively graded “clinically diagnosed.”

^§^ There is a striking difference in CFR among the untreated group, especially in McCulloch’s study [38], which contains a cohort of prisoners responsible for the high CFR. A further group was added to evaluate the impact when the cohort of prisoners is excluded.

An overview on all identified LBRF cases with the according case fatality rates (CFRs) is shown (Table 3).

**Table 3:** Numbers of diagnosed cases and respective CFR.

Cases diagnosed by microscopy and/or PCR			Overall^†^	
	Number of cases	Case fatality rate	Number of cases	Case fatality rate
**Untreated**	*n* = 577	10.2%	*n* = 991	6%
**Untreated**^‡^	*n* = 525	5.5%	*n* = 939	3.1%
**Treated**	*n* = 5,893	4%	*n* = 5,969	4%
**Arsenicals**	*n* = 2,262	5.2%	*n* = 2,338	5%
**Antibiotics**	*n* = 3,631	3.3%	*n* = 3,631	3.3%

^†^ Includes cases that were categorised as clinically diagnosed according to diagnostic grading E.

^‡^ Cohort of prisoners is excluded.

PCR, polymerase chain reaction.

Evaluated antibiotics: Ampicillin, Amikacin, Cefotaxime, Ceftriaxone, Cefuroxime, Chloramphenicol, Chlortetracycline, Clarithromycin, Doxycycline, Erythromycin, Meropenem, Metronidazole, Penicillin, Tetracycline-HCl; evaluated arsenicals: Acetylarsan, Arrhenal, Arsalyt, Arsphenamine *(*Arsenobenzol, Salvarsan), Arsenobillon, Galyl, Ludyl, Mepharsen, Neoarsphenamine (Neosalvarsan, Novarsenobenzol), Neoiacol, Olarsol

**Fig 2:**
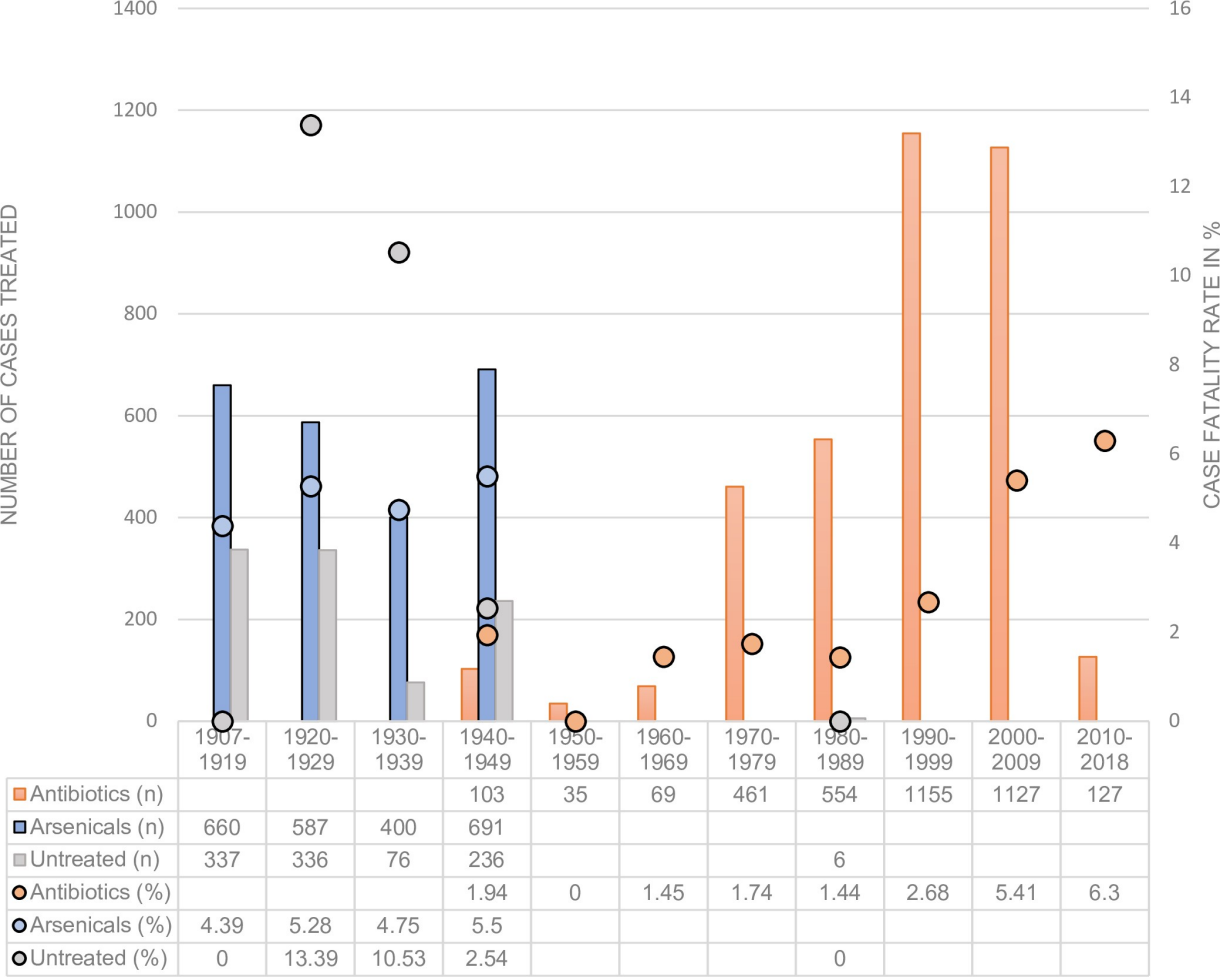
CFR of LBRF cases from the reviewed studies according to treatment modality over time. The dots correspond to the CFR of the respective group. The columns represent the absolute number of cases. The table gives detailed information on the data for each segment of time. Note: Since the data set is compiled from published literature reporting on cases, outbreaks and clinical studies from different regions, different populations as well as different seasons, no trend in annual incidence of LBRF can be inferred.

Fig 2 depicts the CFR of LBRF cases from the reviewed studies according to treatment modality over time.

**Fig 3:**
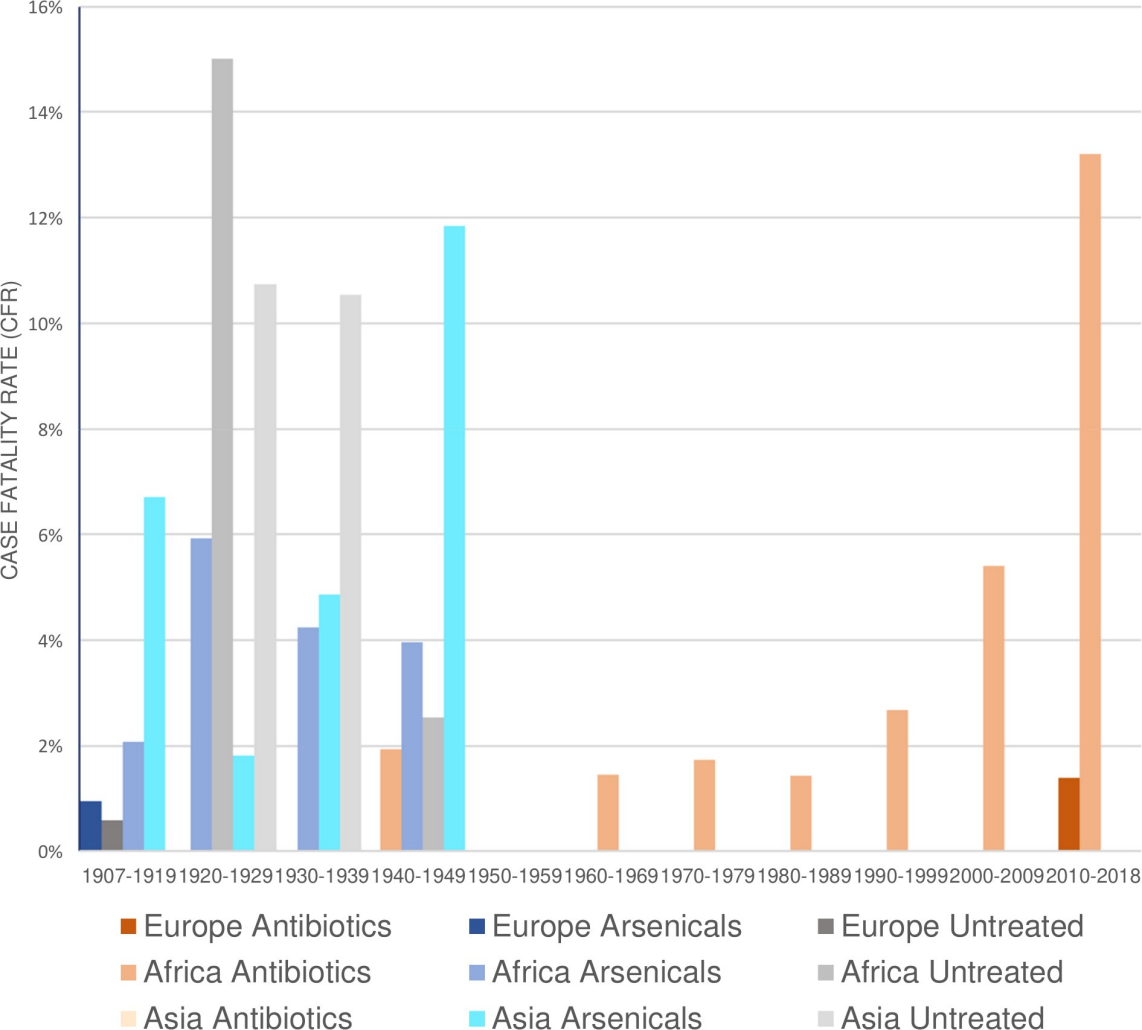
CFR in relation to treatment modality, time, and geographical region.

Between 2010 and 2019, 127 patients were treated with an overall CFR of 6.3%. Of these, 53 were treated in the frame of a study conducted in Ethiopia which reported a CFR of 13.21%. The other patients were treated in Europe (*n* = 72), Saudi Arabia (*n* = 1), and Israel (*n* = 1). Among these, 1 fatal case occurred in Europe, which corresponds to a CFR of 1.35%. An overview on CFR in relation to treatment and geographical region is shown in Fig 3. Reported signs, symptoms, and factors associated with mortality and poor prognosis are summarized in Table 4.

**Table 4:** Risk factors, symptoms, and signs for mortality and poor prognosis reported in literature.

First Author	Year of pub.	Country	Symptoms/Signs/Factors associated with high mortality and poor prognosis	Ref.
**Jouveau-Dubreuil**	1920	China	Poor health condition prior to infection	[35]
**Russell**	1931	Ghana	Jaundice	[46]
**Chang**	1938	China	Jaundice, enlargement of liver	[47]
**Chung**	1939	China	Jaundice, pneumonia	[41]
**El Ramley**	1946	Egypt	Critical health condition due to late admission, jaundice, pneumonic signs	[48]
**Gaud**	1948	North Africa	Poor health condition prior to infection, epistaxis, hemoptysis, intestinal hemorrhages	[49]
**Salih**	1977	Ethiopia	Epistaxis, jaundice	[50]
**Borgnolo**	1993	Ethiopia	Abdominal guarding, jaundice, disturbed consciousness, high spirochetemia	[51]
**Seboxa**	1995	Ethiopia	JHR	[7]
**Mitiku**	2002	Ethiopia	JHR	[52]
**Ramos**	2004	Ethiopia	Vomiting, admission more than 4 days after the onset of symptoms	[53]
**Nordmann**	2018	Ethiopia	Critical health condition due to late admission	[54]

JHR, Jarisch–Herxheimer reaction; Pub, publication; Ref, reference.

### Jarisch–Herxheimer reaction (JHR)

After full-text assessment, 184 publications were eligible. After applying subsearch inclusion criteria for "JHR," 42 publications were included and are summarized in Table 5.

**Table 5:** Details of all the included studies that published data on the JHR, in chronological order and according to diagnostic grading.

First Author	Year of pub.	Country	Diagnostic Grade	Number of patients	JHR *n* (%)	Study focus	Ref.
Schofield	1968	Ethiopia	B	10	10 (100)	JHR	[19]
Bryceson	1970	Ethiopia	B	62	62 (100)	JHR	[4]
Warrell	1970	Ethiopia	B	19	19 (100)	JHR	[20]
Bryceson	1972	Ethiopia	B	9	9 (100)	JHR	[55]
Knaack	1972	Ethiopia	B	25	14 (56)	Treatment	[56]
Perine	1974	Ethiopia	B	26	26 (100)	Treatment	[57]
Galloway	1977	Ethiopia	B	15	15 (100)	JHR	[58]
Salih	1977	Sudan	B	160	23 (14.4)	Treatment	[59]
Butler	1979	Ethiopia	B	90	78 (86.7)	JHR, endotoxins	[60]
Butler	1980	Ethiopia	B	11	9 (81.8)	JHR, phagocytosis	[61]
Perine	1983	Ethiopia	B	377	377 (100)	Treatment, 6 untreated cases	[45]
Teklu	1983	Ethiopia	B	33	33 (100)	JHR	[62]
Warrell	1983	Ethiopia	B	12	12 (100)	JHR	[63]
Zein	1987	Ethiopia	B	132	28 (21.2)	JHR	[64]
Brown	1988	Somalia	B	37	0 (0)	Symptoms, Epidemiology	[65]
Daniel	1992	Ethiopia	B	80	26 (32.5)	Children	[66]
Gebrehiwot	1992	Ethiopia	B	120	48 (40)	Treatment	[67]
Mekasha	1992	Ethiopia	B	63	10 (15.9)	Children	[68]
Negussie	1992	Ethiopia	B	17	14 (82.4)	JHR	[69]
Borgnolo	1993	Ethiopia	B	103	63 (61.2)	Children	[51]
Borgnolo	1993	Ethiopia	B	389	168 (43.2)	Symptoms, epidemiology	[6]
Knox	1994	Ethiopia	E	51	26 (51)	JHR, anti-TNF	[25]
Cuevas	1995	Ethiopia	B	25	14 (56)	JHR, cytokines	[70]
De Jong	1995	Southern Sudan	B	22	4 (18.2)	Epidemiology	[71]
Seboxa	1995	Ethiopia	B	184	54 (29.4)	Treatment	[7]
Fekade	1996	Ethiopia	B	49	36 (73.5)	JHR, anti-TNF	[23]
Remick	1996	Ethiopia	B	19	19 (100)	JHR	[72]
Cooper	2000	Ethiopia	B	49	48 (98)	JHR	[73]
Mitiku	2002	Ethiopia	B	262	83 (31.7)	Epidemiology	[52]
Tewdros	2002	Ethiopia	B	106	80 (75.5)	Symptoms, epidemiology	[74]
Alfaifi	2014	Saudi Arabia	B	1	1 (100)	Case report	[75]
Hoch	2015	Germany	14A,1B	15	10 (66.7)	Epidemiology	[76]
Wilting	2015	Netherlands	A	2	2 (100)	Case series	[77]
Ciervo	2016	Italy	A	3	1 (33.3)	Case series	[78]
Costescu	2016	Belgium	B	2	1 (50)	Case series	[79]
Lucchini	2016	Italy	A	5	2 (40)	Case series	[80]
Osthoff	2016	Switzerland	A	4	1 (25)	Case series	[81]
Seilmaier	2016	Germany	A	25	22 (88)	Case series	[82]
Von Both	2016	Germany	A	1	1 (100)	Case report	[83]
Zammarchi	2016	Italy	A	1	1 (100)	Case report	[84]
Hytonen	2017	Finland	A	2	2 (100)	Case series	[85]
Nordmann	2018	Ethiopia	B	54	4 (7.4)	Symptoms, epidemiology	[54]

JHR, Jarisch–Herxheimer reaction; Pub, publication; Ref, reference; TNF, tumor necrosis factor.

We identified 3 studies [24,59,86] in which already previously published patient data were republished [4,23,50]. Data from these 3 studies were compiled (data of identical patients/cases).

Overall, JHR was observed in 1,452 of the 2,618 reported cases, corresponding to an incidence rate of 55.8%. Of note, the incidence rate of JHR rate in studies focusing on JHR (n_cases_ = 603) versus studies not focusing on JHR (n_cases_ = 2015) was 71.78% and 50.62%, respectively.

### Pregnancies

After full-text assessment of the eligible 184 publications, 14 proved eligible after applying subsearch inclusion criteria for "pregnancies." The characteristics of these 14 included studies are summarized in Table 6.

**Table 6:** Details of all the studies including data on LBRF infection during pregnancy, in chronological order and according to grade of diagnostic certainty.

First Author	Year of pub.	Country	Grade of diagnostic certainty	Number of pregnancies	Adverse pregnancy outcomes *n* (%)	Ref.
Jukes	1912	India	B	1	1 (100)	[87]
Sergent	1922	Algeria	E^†^	30	4 (13.3)	[37]
McCulloch	1925	Nigeria	B	2	2 (100)	[38]
Robertson	1932	China	B	5	3 (60)	[88]
Chung	1939	China	B	2	1 (50)	[41]
Benhamou	1945	Algeria	E^‡^	54	49 (90.7)	[89]
El Ramley	1946	Egypt	B	79	68 (86.1)	[48]
Ingraham	1946	Egypt	B	2	2 (100)	[44]
Garnham	1947	Kenya	B	3	3 (100)	[12]
Bryceson	1970	Ethiopia	B	6	3 (50)	[4]
Brown	1988	Somalia	B	4	2 (50)	[65]
Gebrehiwot	1992	Ethiopia	B	2	1 (50)	[67]
Borgnolo	1993	Ethiopia	B	15	6 (40)	[6]
De Jong	1995	Southern Sudan	B	1	1 (100)	[71]

LBRF, louse-borne relapsing fever; Pub, publication; Ref, reference.

^†^ The use of microscopy is mentioned in the descriptions of a few cases. However, whether microscopy was systematically used or only in some cases is unclear. Thus, the study was conservatively graded “clinically diagnosed.”

^‡^ No information on the use of microscopy. Thus, the study was graded “clinically diagnosed.”

**Fig 4:**
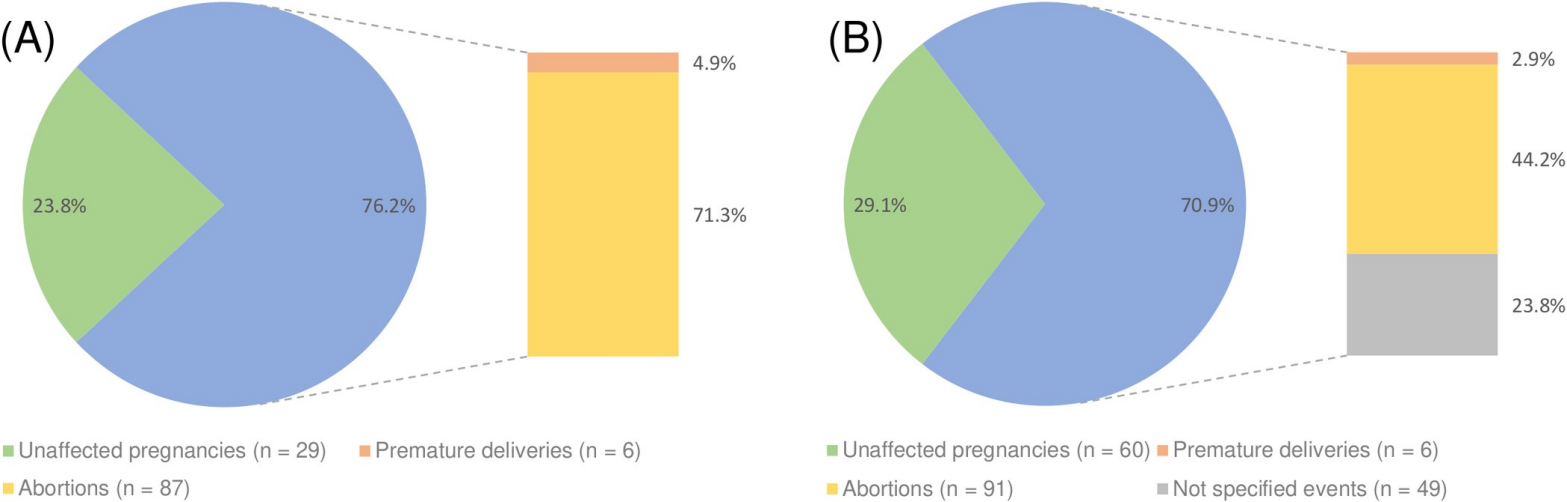
Adverse pregnancy outcomes in LBRF cases. (A) Microscopically diagnosed cases. (B) Microscopically and clinically diagnosed cases. One study reported adverse pregnancy outcomes without specifying them (gray color) [89].

In total, 206 pregnancies and their outcome were reported in the 14 included studies (Table 6): The overall incidence of an adverse pregnancy outcome was 70.9% (*n* = 146). When considering only cases reported by studies graded at least “B” (*n* = 122 pregnancies), adverse pregnancy outcomes were reported in 76.2% of the cases (*n* = 93) (Fig 4).

## Discussion

### Mortality

#### Observational factors potentially related to high case fatality

High mortality rates in untreated cases (30% to 80%) [4–12] were mostly traced back to reports of epidemics based either on observations by the authors or on renditions of observational reports by colleagues. In both cases, these are rough estimations without any diagnostic evidence [5,8,90]. In other instances, high rates trace back to coinfections with other diseases [38,90–93]. Additionally, some authors attribute the striking differences in reported mortality rates to the general poor health and nutritional condition of the affected population, the lack of medical and sanitary measures, and sociopolitical factors like war [10–12,35,49,92]. Exemplarily, observations from China noted the difference between an outbreak among “robust” soldiers and among poor villagers, where mortality had been near 0% and 50%, respectively [35]. CFRs and the course of the disease depend on the general condition and health of the population [94,95]. Additionally, a study found the disease to be more common and have an atypical course during famine years, compared to other years [94]. However, some authors noted that the disease is relatively benign, given appropriate treatment within a short time after the manifestation of symptoms [49], as Gaud and Morgan’s quote of Charles Nicolle shows: “No one need to die of relapsing fever provided that it is diagnosed in time” [49].

#### Untreated cases and analysis of CFR

Anecdotal CFR of up to 80% are described in literature [4–12]; however, evidence-based data only show CFR up to 47.1% (Table 2). One study stands out with a CFR of 47.1%, which is the only data reaching the reported high CFRs (Table 2). The study investigated relapsing fever in Nigeria, including 52 of the untreated cases which originated from a prison in Kano, among whom 30 died resulting in a mortality rate of 57.69%. The reasons for the strikingly high CFR remain unknown. The only information available is that the author conducted weekly inspections only on the inmates. Coinfections or poor nutritional, medical, and health conditions cannot be excluded. Only 2 patients died out of the remaining 16 reported untreated patients, accounting for a mortality rate of 12.5% [38]. The prison series may be regarded as an extreme scenario. Interestingly, CFR drops roughly by half, when the extreme scenario is excluded (Table 3). The overall CFR for untreated cases was only slightly higher than the overall CFR for patients treated with arsenicals. When excluding the prison series, the overall CFR in untreated patients is 3.1%, which is lower than the overall CFR for patients treated with antibiotics. This demonstrates the impact of an insufficiently investigated extraordinary scenario on reported CFRs. Still, the overall results strikingly contrast with the rates of 40% to 80% that have been reported earlier, and published available data suggest that untreated LBRF may not be as deadly as reported earlier. Since the mortality rate is influenced by many factors, the numbers given may not reflect every situation. However, they reflect the overall rates and tendencies from all confirmed LBRF cases that were identified through published literature research.

#### Benefit of therapy

Since antibiotic treatment of LBRF may trigger a strong and possibly even lethal JHR, the benefit of specific therapy has been questioned in certain situations. However, there is also no evidence to support a beneficial outcome when treatment is withheld. As suggested by Wolff and agreed to by Bryceson and colleagues, patients in a critical condition should primarily be treated symptomatically. Only after the attack should specific treatment be given [4,95]. Today, this approach of “resuscitate first—but then treat” appears to be the most widely accepted consensus regarding the management of LBRF patients.

#### Rise of CFR in treated cases

Figs 2 and 3 demonstrate that antibiotic treatment reduced the CFR of LBFR compared to untreated cases and treatment using arsenicals in mid-20th century. However, the figures also show that over the past few decades, the CFR reported in the literature seems to have increased in patients treated with antibiotics. Fig 3 demonstrates both the geographic distribution and the increased number of studies publishing cases from the African continent, primarily East Africa. The patients treated in Europe were immigrants and refugees that were taken care of in European hospital centers. The patients in Ethiopia were mostly men from poor socioeconomic status, who could not afford costs for diagnosis and treatment. The poverty and the lack of awareness of LBRF prevented or delayed admission to medical care [54]. Two factors may have been responsible for this striking difference in CFR: on the one hand, the difference in the general condition and nutritional status of the 2 populations; on the other hand, the availability of medical care to the people affected. Unknown factors such as additional coinfections in the population from Arsi (Ethiopia) may have contributed. The rise over the past few decades may indicate a decline in health status of the affected population in Ethiopia. Further studies investigating the reasons for these high CFR in Ethiopia are needed.

### The Jarisch–Herxheimer reaction

#### “Resolvement by crisis” in untreated cases

In LBRF, a JHR-like reaction, often called crisis, has been observed after treatment since the beginning of the last century [34,41,47, 88,95–98]. Even untreated cases have been reported to exhibit a spontaneous resolvement of fever through crisis, which has been recognized by researchers to resemble the features and severity of the JHR in certain cases [50,63,99]. A study comparing the effects of different antibiotic treatments followed 6 untreated cases that had been admitted during this spontaneous crisis. These observations showed that the JHR was indistinguishable from the spontaneous crisis [45]. Unfortunately, there are no published studies which have investigated the process in detail during the natural course of the disease. It would be of great academic interest to compare the clinical and laboratory changes during the spontaneous reaction and the reaction after antibiotic treatment.

#### Detection bias

Lack of standardized defining criteria: The observed incidence rate of JHR reported in studies range from 0% to 100% (Table 5). A reason for the discrepancies may be that there was neither a uniform protocol or guidelines for the classification of severity, nor a standardized definition or threshold from which point on the observations may be regarded as a JHR. Only 3 studies noted a predefined grading system [7,23,24,55]. A study stated that comparison is hampered due to this issue and noted further that some studies include critically ill patients, while others exclude this group of patients [7]. Another study reflected on its own results of a JHR rate of only 7.4% and states that these low results may have been caused by the lack of defining criteria for the occurrence of a JHR [54].

Influence of monitoring: Our data suggest that studies investigating JHR report higher incidence rates than studies which primarily focus on other aspects (Table 5). A review suggested that JHRs are often not recognized, thus underreported and easily overlooked [18]. Most studies investigating the pathophysiology after treatment included close monitoring of patients, often observing most of the parameters that are likely to change during JHR, hence increasing the likelihood of the reaction being detected. Mild reactions, on the other hand, may be easily overlooked without close monitoring, considering that they already peak 4 hours after treatment [18]. The issue may be demonstrated in the 37 cases studied by Brown and colleagues, who reported no JHR in the series. However, there was no information available about a threshold or definition used to identify a potential JHR. Intriguingly, rigors were observed in 30% of patients. The aims of the study were to survey a refugee camp and identify clinical criteria for diagnosing LBRF in the absence of laboratory facilities. As the resources in the given situation were not suitable for proper monitoring, the question may retrospectively be raised as to whether a certain number of JHRs could have been missed [65]. Another issue may be the degree of experience of the involved personnel and scientific researchers with diagnosing JHR. Perine, an author focusing on JHR [20,55,63,100], published JHR rates of 100% when studying the treatment of LBRF both in 1974 and 1983 (Table 5). Salih reported a JHR rate of 14.4% when researching treatment of LBRF in 1977. It seems likely that experience in detection influenced the striking difference. The pathophysiology of the reaction after treatment suggests a chance of missing the diagnosis when vital signs are monitored only 3 to 4 times a day, which may be assumed standard procedure in hospitalized stable patients.

Developing a grading system: There is a need to develop and widely apply a clear definition as to what should be regarded as a JHR. Until then, published data can hardly be compared to one another, since most studies do not report the process of monitoring and the threshold from which they consider a reaction as a JHR. Seboxa and Rahlenbeck defined a JHR as an increase in body temperature (>1°C), tachycardia, and a drop in systolic and diastolic blood pressure greater than 10 mmHg during 4 hours after treatment [7]. If further factors were to be added, the JHR might be objectified more precisely. An initial rise in body temperature, a rise in heart rate, rigors, an initial rise in blood pressure, tachypnoea, a late drop in arterial blood pressure, and a decline of body temperature could be easily measured and added to a protocol. If some of these changes were to be detected after treatment, a JHR might be diagnosed.

Redefining the reaction after treatment: The data suggest that defining the JHR as a reaction after antibiotic treatment should be reconsidered. It seems likely that there is always a reaction, which is altered by administering antimicrobial drugs, and thus more likely to be detected. Detection is favored by the circumstance of patients being monitored to various degrees in a medical facility.

### Pregnancies

#### Lack of data

To the best of our knowledge, this is the first review regarding the question of the influence of the *B*. *recurrentis* infection on pregnancy outcomes. Finding an explanation for these high rates is difficult due to scarce data. Most cases are simply noted in the reports without any further information [37,38,44,65,67,71].

#### Descriptions of cases and the question of congenital infection

However, there are a few cases of LBRF described in detail. In one case, a mother was admitted on the third day of fever with the pregnancy intact. On the fifth day of fever, labor began just as her body temperature began to fall, and she gave birth to a stillborn child. The second case was of a woman admitted in labor on the seventh day of fever, when birth was given to a premature infant. This infant developed a fever with spirochetemia on the sixth day and died on the seventh day after birth. The postmortem examination found jaundiced conjunctivae, a collapsed left lung, and a congested right lung. Spirochetes were found in sections of the spleen, kidney, liver, lung and, as noted, “suprarenal.” Additional direct films were taken from the umbilical cord, lungs, liver, kidneys, and spleen, which all revealed positive results. Regarding the route of transmission, the author found the evidence to be inconclusive. When examining the placenta, the maternal surface contained a large number of spirochetes in the maternal blood sinuses. However, no positive results were yielded from the fetal vili. Nevertheless, all possible sources of infection other than intra-utero were found to be unlikely, suggesting an intrauterine infection as a probable source of infection in this case [88]. A similar scenario was described by Chung and Chang, in which a premature infant died within a week after birth. This was supposed to be a case of congenital infection. The mother was admitted with relapsing fever during parturition [41]. Another study gave a detailed account on the adverse pregnancy outcomes. The report concludes that both age of the mother and age of gestation had no influence on adverse events. Adverse events were observed at any gestation week during pregnancy. Further, the study reported spirochetes in a stillborn infant’s blood, obtained from its heart, and intrauterine infection was suspected in 2 new born infants [48]. Bryceson and colleagues stated that abortion and miscarriage seem to be usual outcomes of pregnancies where the mother is infected with *B*. *recurrentis*. The authors further referenced a colleague, El Ramley A., who found congenital infection and abortion to be a standard in his studies [4].

#### Association between gestational progress and adverse outcome?

In one publication, all adverse events took place before the 20th week of gestation, and no stillbirths had been observed past that point [6]. By contrast, several studies reported adverse pregnancy outcomes past that time: 2 stillbirths in the ninth month of pregnancy [65], a premature delivery in the seventh month of gestation [41], a premature delivery at 7.5 months of gestation [44], a stillbirth in the fifth month, and in the case of a further 2 women, a stillbirth and a premature delivery in the seventh month of gestation [88]. The evidence suggests that adverse pregnancy outcomes may occur at any time, as another study has observed [89].

#### Need for further studies

Results of the analysis demonstrated an enormous rate of adverse pregnancy outcomes for pregnant women infected with *B*. *recurrentis* (Fig 4). While most aspects of LBRF were extensively researched, the effect on pregnant women and the unborn child has not been investigated yet. Factors that influence the risk of adverse pregnancy outcome during LBRF are currently unknown or not retrievable in published literature. The findings suggest a need for studies specifically looking at LBRF during pregnancy. Regarding adverse pregnancy outcomes during TBRF infection, rates between 30% and 44% were reported [27,28,101–103]. Larsson and colleagues recently demonstrated the effects of *Borrelia duttonii* infection in a mouse model, where spirochetes frequently caused congenital infection [102]. A treatable, neglected disease with a probability of up to 75% of adverse pregnancy outcomes should receive close attention, especially when dealing with women in, or from, endemic countries. Parallels can be drawn to similar neglected diseases, such as scrub typhus and murine typhus, in which a poor neonatal outcome (stillborn, premature, and/or small for gestation age) has been reported in 43.6% and 33.3%, respectively [104]. There is a need for further studies and increased awareness, especially among women in endemic countries.

**Key learning points**

- Reported CFRs in Africa have been rising throughout the past few decades.
- There is no standardized protocol on diagnosing JHR and reported JHR rates depend on the focus of the study, monitoring of patients, and awareness.
- Three out of 4 pregnancies are negatively affected by LBRF.

**Top five papers**

- Warrell DA. Louse-borne relapsing fever (Borrelia recurrentis infection). Epidemiol Infect. 2019;147:e106. doi: 10.1017/S0950268819000116
- Bryceson ADM, Parry EHO, Perine PL, Warrell DA, Vukotich D, Leithead CS. A clinical and laboratory study of 62 cases in ethiopia and a reconsideration of the literature1. QJM. 1970;39(1):129–70. doi: 10.1093/oxfordjournals.qjmed.a067198
- Felsenfeld O. Borrelia; Strains, Vectors, Human and Animal Borreliosis. St. Louis: Warren H. Green; 1971.
- Butler T. The Jarisch-Herxheimer Reaction After Antibiotic Treatment of Spirochetal Infections: A Review of Recent Cases and Our Understanding of Pathogenesis. Am J Trop Med Hyg. 2017;96(1):46–52. PubMed PMID: CCC:000397822900010.
- Guerrier G, Doherty T. Comparison of antibiotic regimens for treating louse-borne relapsing fever: A meta-analysis. Trans R Soc Trop Med Hyg. 2011;105(9):483–90. doi: http://dx.doi.org/10.1016/j.trstmh.2011.04.004. PubMed PMID: 51548792.

## Supporting information

S1 Checklist PRISMA checklist. 27-item checklist for systematic reviews.
(DOC)

S1 Text Review Protocol. Established to conduct this systematic review.
(DOCX)

S2 Text Data Extraction Sheet. Used for screening and selecting eligible publications.
(DOCX)

S3 Text References before sub-search. Included and excluded references for qualitative synthesis, before a subsearch was conducted on (i) outcome, (ii) JHR, and (iii) impact on pregnancies.
(DOCX)

S4 Text References–mortality. Included and excluded references in mortality section.
(DOCX)

S5 Text References–JHR. Included and excluded references in JHR section.
(DOCX)

S6 Text References–pregnancies. Included and excluded references in pregnancies section.
(DOCX)

S1 Fig PRISMA flow diagram. (PDF)

S1 Data Data extracted from included studies. Excel spreadsheet containing, in separate sheets, the underlying numerical data.
(XLSX)
