# Supplementary material for: Louse-borne relapsing fever—A systematic review and analysis of the literature: Part 2—Mortality, Jarisch–Herxheimer reaction, impact on pregnancy
Source: PLoS Negl Trop Dis. 2021 Mar 11;15(3):e0008656. doi: 10.1371/journal.pntd.0008656 (PMC7951929; doi:10.1371/journal.pntd.0008656)
Supplement: S3 Text — Included and excluded references for qualitative synthesis, before a subsearch was conducted on (i) outcome, (ii) JHR, and (iii) impact on pregnancies. (DOCX) [file pntd.0008656.s004.docx]

**S3 Text. Included and excluded references for qualitative synthesis, before a sub-search was conducted on (i) outcome, (ii) JHR and (iii) impact on pregnancies**

**Included references**

[1-184]

**Excluded references**

Did not provide sufficient information for species identification

[185-207]

Tick borne cases

[208-238]

Language

[239-247]

Did not include confirmed human LBRF cases

[248-610]

Duplicates

[611-618]

Date before 1907

[619, 620]

Not retrievable

[621-638] -683]

1. Nitzan O, Blum A, Marva E, Katz A, Tzadok BS, Nachum-Biala Y, et al. Case report: Infectious diseases in pilgrims visiting the holy land. American Journal of Tropical Medicine and Hygiene. 2017;97(2):611-4. doi: <http://dx.doi.org/10.4269/ajtmh.17-0097>. PubMed PMID: 617714405.

2. Jochum J, Tannich E, Tappe D, Schmiedel S. A Somali refugee with fever and abnormal blood smear. Internist. 2017;58(3):287-92. doi: 10.1007/s00108-016-0154-3.

3. Hytonen J, Khawaja T, Gronroos JO, Jalava A, Meri S, Oksi J. Louse-borne relapsing fever in Finland in two asylum seekers from Somalia. Apmis. 2017;125(1):59-62. doi: <http://dx.doi.org/10.1111/apm.12635>. PubMed PMID: 613575266.

4. Grecchi C, Zanotti P, Pontarelli A, Chiari E, Tomasoni LR, Gulletta M, et al. Louse-borne relapsing fever in a refugee from Mali. Infection. 2017;45(3):373-6. doi: <http://dx.doi.org/10.1007/s15010-017-0987-2>. PubMed PMID: 614317686.

5. Cutuli SL, De Pascale G, Spanu T, Dell'Anna AM, Bocci MG, Pallavicini F, et al. Lice, rodents, and many hopes: A rare disease in a young refugee. Critical Care. 2017;21 (1) (no pagination)(81). doi: <http://dx.doi.org/10.1186/s13054-017-1666-5>. PubMed PMID: 615072527.

6. Bloch-Infanger C, Battig V, Kremo J, Widmer AF, Egli A, Bingisser R, et al. Increasing prevalence of infectious diseases in asylum seekers at a tertiary care hospital in Switzerland. PLoS One. 2017;12(6):e0179537. doi: 10.1371/journal.pone.0179537. PubMed PMID: BIOSIS:PREV201700716182.

7. Zammarchi L, Antonelli A, Bartolini L, Pecile P, Trotta M, Rogasi PG, et al. Louse-Borne Relapsing Fever with Meningeal Involvement in an Immigrant from Somalia to Italy, October 2015. Vector-Borne and Zoonotic Diseases. 2016;16(5):352-5. doi: <http://dx.doi.org/10.1089/vbz.2015.1928>. PubMed PMID: 610464500.

8. Von Both U, Alberer M. Borrelia recurrentis infection. New England Journal of Medicine. 2016;375(5):e5. doi: <http://dx.doi.org/10.1056/NEJMicm1513366>. PubMed PMID: 611576321.

9. Seilmaier M, Guggemos W, Wieser A, Fingerle V, Balzer L, Fenzl T, et al. 25 Cases of Louse-borne Relapsing Fever in Refugees from East Africa. Deutsche Medizinische Wochenschrift. 2016;141(14):E133-E42. PubMed PMID: CCC:000381007800002.

10. Osthoff M, Schibli A, Fadini D, Lardelli P, Goldenberger D. Louse-borne relapsing fever - report of four cases in Switzerland, June-December 2015. BMC Infectious Diseases. 2016;16 (1) (no pagination)(210). doi: <http://dx.doi.org/10.1186/s12879-016-1541-z>. PubMed PMID: 610362799.

11. Lucchini A, Lipani F, Costa C, Scarvaglieri M, Balbiano R, Carosella S, et al. Louseborne relapsing fever among East African refugees, Italy, 2015. Emerging Infectious Diseases. 2016;22(2):298-301. doi: <http://dx.doi.org/10.3201/eid2202.151768>. PubMed PMID: 607873433.

12. Keller C, Zumblick M, Streubel K, Eickmann M, Muller D, Kerwat M, et al. Hemorrhagic diathesis in borrelia recurrentis infection imported to Germany. Emerging Infectious Diseases. 2016;22(5):917-9. doi: <http://dx.doi.org/10.32032/eid2205.151557>. PubMed PMID: 609995922.

13. Hertting O, Bennet R, Luthander J, Eriksson M. Infection-related hospitalisations in refugee children-a one year study from Northern Stockholm. Cogent Medicine Conference: 8th Excellence in Pediatrics Conference. 2016;3(1). doi: <http://dx.doi.org/10.1080/2331205X.2016.1265203>. PubMed PMID: 614265435.

14. Darcis G, Hayette MP, Bontems S, Sauvage AS, Meuris C, Van Esbroeck M, et al. Louse-borne relapsing fever in a refugee from Somalia arriving in Belgium. Journal of Travel Medicine. 2016;23(3). doi: <http://dx.doi.org/10.1093/jtm/taw009>. PubMed PMID: 614260542.

15. Costescu Strachinaru DI, Cambier J, Kandet-Yattara H, Konopnicki D. Relapsing fever in asylum seekers from Somalia arriving in Belgium in August 2015. Acta Clinica Belgica: International Journal of Clinical and Laboratory Medicine. 2016;71(5):353-5. doi: <http://dx.doi.org/10.1080/17843286.2016.1157942>. PubMed PMID: 612314718.

16. Colomba C, Scarlata F, Di Carlo P, Giammanco A, Fasciana T, Trizzino M, et al. Fourth case of louse-borne relapsing fever in Young Migrant, Sicily, Italy, December 2015 Mini Review Article. Public Health. 2016;139:(5p). doi: 10.1016/j.puhe.2016.05.019. PubMed PMID: 118802975.

17. Ciervo A, Mancini F, Di Bernardo F, Giammanco A, Vitale G, Dones P, et al. Louseborne relapsing fever in young migrants, sicily, Italy, july-september 2015. Emerging Infectious Diseases. 2016;22(1):152-3. doi: <http://dx.doi.org/10.3201/eid2201.151580>. PubMed PMID: 607341375.

18. Benz R, Majer S. Unexpected cause of high fever in the blood film. Blood. 2016;127(18):2264. doi: <http://dx.doi.org/10.1182/blood-2016-01-691980>. PubMed PMID: 610329290.

19. Antinori S, Mediannikov O, Corbellino M, Grande R, Parravicini C, Bestetti G, et al. Louse-Borne Relapsing Fever (Borrelia recurrentis) in a Somali Refugee Arriving in Italy: A Re-emerging Infection in Europe? PLoS Neglected Tropical Diseases. 2016;10 (5) (no pagination)(e0004522). doi: <http://dx.doi.org/10.1371/journal.pntd.0004522>. PubMed PMID: 610558073.

20. Wilting KR, Stienstra Y, Sinha B, Braks M, Cornish D, Grundmann H. Louse-borne relapsing fever (Borrelia recurrentis) in asylum seekers from Eritrea, The Netherlands, July 2015. Eurosurveillance. 2015;20(30):3. PubMed PMID: 605514444.

21. Hoch M, Wieser A, Loscher T, Margos G, Purner F, Zuhl J, et al. Louse-borne relapsing fever (Borrelia recurrentis) diagnosed in 15 refugees from northeast Africa: Epidemiology and preventive control measures, Bavaria, Germany, July to October 2015. Eurosurveillance. 2015;20(42). doi: <http://dx.doi.org/10.2807/1560-7917.ES.2015.20.42.30046>. PubMed PMID: 606659340.

22. Goldenberger D, Claas GJ, Bloch-Infanger C, Breidthardt T, Suter B, Martinez M, et al. Louse-borne relapsing fever (Borrelia Recurrentis) in an Eritrean refugee arriving in Switzerland, August 2015. Eurosurveillance. 2015;20(32). PubMed PMID: 605710959.

23. Gitta SN, Kamadjeu R, Mwesiga A. Proceedings of the 2013 AFENET Scientific Conference - Posters sessions. Pan Afr Med J. 2015;21. doi: 10.11604/pamj.2015.21.209.7259. PubMed Central PMCID: PMC4586171.

24. Yimer M, Mulu W, Ayalew W, Abera B. Louse-borne relapsing fever profile at Felegehiwot referral hospital, Bahir Dar city, Ethiopia: a retrospective study. BMC research notes. 2014;7:250. doi: <http://dx.doi.org/10.1186/1756-0500-7-250>. PubMed PMID: 602155400.

25. Yimer M, Abera B, Mulu W, Bezabih B, Mohammed J. Prevalence and risk factors of louse-borne relapsing fever in high risk populations in Bahir Dar city Northwest, Ethiopia. BMC research notes. 2014;7:615. doi: <http://dx.doi.org/10.1186/1756-0500-7-615>. PubMed PMID: 604824174.

26. Alfaifi AA, Masoodi I, Alzaidi O, Hussain S, Khurshid S, Sirwal IA. Spirocheatal shock syndrome. Indian Journal of Medical Microbiology. 2014;32(2):183-5. doi: <http://dx.doi.org/10.4103/0255-0857.129822>. PubMed PMID: 372815941.

27. Chandra EM, Mandefro Y, Yinges S, Chere BT. Is over-dependence on malaria RDTS disguising morbidity due to other deadly hemoparasites at peripheral health facilities? American Journal of Tropical Medicine and Hygiene. 2013;1):241. PubMed PMID: 71312626.

28. Aarsland SJ, Castellanos-Gonzalez A, Lockamy KP, Mulu-Droppers R, Mulu M, White AC, et al. Treatable Bacterial Infections Are Underrecognized Causes of Fever in Ethiopian Children. Am J Trop Med Hyg. 2012;87(1):128-33. doi: 10.4269/ajtmh.2012.12-0171. PubMed PMID: 22764303; PubMed Central PMCID: PMC3391037.

29. Ramos JM, Malmierca E, Reyes F, Tesfamariam A. Louse-borne relapsing fever in Ethiopian children: Experience of a rural hospital. Tropical Doctor. 2009;39(1):34-6. doi: <http://dx.doi.org/10.1258/td.2008.080157>. PubMed PMID: 354731406.

30. Ramos JM, Malmierca E, Reyes F, Tesfamariam A. Results of a 10-year survey of louse-borne relapsing fever in southern Ethiopia: A decline in endemicity. Annals of Tropical Medicine and Parasitology. 2008;102(5):467-9. doi: <http://dx.doi.org/10.1179/136485908X300887>. PubMed PMID: 352008728.

31. Ramos JM, Reyes F, Tesfamariam A, Malmierca E. Louse-borne relapsing fever and malaria co-infection in Ethiopia. Tropical Doctor. 2007;37(2):121-2. doi: <http://dx.doi.org/10.1258/004947507780609419>. PubMed PMID: 354729852.

32. Melaku Z, Alemayehu M, Oli K, Tizazu G. Pattern of admissions to the Medical Intensive Care Unit of Addis Ababa University Teaching Hospital. Ethiopian Medical Journal. 2006;44(1):33-42.

33. Ramos JM, Malmierca E, Reyes F, Wolde W, Galata A, Tesfamariam A, et al. Characteristics of louse-borne relapsing fever in Ethiopian children and adults. Annals of Tropical Medicine and Parasitology. 2004;98(2):191-6. doi: <http://dx.doi.org/10.1179/000349804225003136>. PubMed PMID: 38388887.

34. Mitiku K, Mengistu G. Relapsing fever in Gondar, Ethiopia. East African Medical Journal. 2002;79(2):85-7.

35. Cobey FC, Goldbarg SH, Levine RA, Patton CL. Short report: Detection of Borrelia (relapsing fever) in rural Ethiopia by means of the quantitative buffy coat technique. American Journal of Tropical Medicine and Hygiene. 2001;65(2):164-5. PubMed PMID: 32744225.

36. Porcella SF, Raffel SJ, Schrumpf ME, Schriefer ME, Dennis DT, Schwan TG. Serodiagnosis of louse-borne relapsing fever with glycerophosphodiester phosphodiesterase (GlpQ) from Borrelia recurrentis. Journal of Clinical Microbiology. 2000;38(10):3561-71. PubMed PMID: 30768416.

37. Cooper PJ, Fekade D, Remick DG, Grint P, Wherry J, Griffin GE. Recombinant human interleukin-10 fails to alter proinflammatory cytokine production or physiologic changes associated with the Jarisch-Herxheimer reaction. Journal of Infectious Diseases. 2000;181(1):203-9. doi: <http://dx.doi.org/10.1086/315183>. PubMed PMID: 30049050.

38. Orloski K, Tharmaphornpilas P, O'Leary D, Ryan M, Schriefer M, Shoo R, et al. Epidemic louse-borne relapsing fever, Rumbek County, Sudan, 1998-1999. American Journal of Tropical Medicine and Hygiene. 1999;61(3 SUPPL.):223-4. PubMed PMID: BIOSIS:PREV199900470785.

39. Cutler SJ, Moss J, Fukunaga M, Wright DJM, Fekade D, Warrell D. Borrelia recurrentis characterization and comparison with relapsing- fever, lyme-associated, and other Borrelia spp. International Journal of Systematic Bacteriology. 1997;47(4):958-68. PubMed PMID: 27441217.

40. Remick DG, Negussie Y, Fekade D, Griffin G. Pentoxifylline fails to prevent the Jarisch-Herxheimer reaction or associated cytokine release. Journal of Infectious Diseases. 1996;174(3):627-30. PubMed PMID: 26299701.

41. Mekasha A, Meharie S. Outbreak of louse-borne relapsing fever in Jimma, south western Ethiopia. East African medical journal. 1996;73(1):54-8. PubMed PMID: 126225733.

42. Fekade D, Knox K, Hussein K, Melka A, Lalloo DG, Coxon RE, et al. Prevention of Jarisch-Herxheimer reactions by treatment with antibodies against tumor necrosis factor α. New England Journal of Medicine. 1996;335(5):311-5. doi: 10.1056/NEJM199608013350503 FULL TEXT LINK <http://dx.doi.org/10.1056/NEJM199608013350503>. PubMed PMID: 1996331136; PubMed Central PMCID: PMC8663853.

43. Seboxa T, Rahlenbeck S. Treatment of louse-borne relapsing fever with low dose penicillin or tetracycline: a clinical trial. Scandinavian journal of infectious diseases [Internet]. 1995; 27(1):[29-31 pp.]. Available from: <http://onlinelibrary.wiley.com/o/cochrane/clcentral/articles/105/CN-00115105/frame.html>.

44. De Jong J, Wilkinson RJ, Schaeffers P, Sondorp HE, Davidson RN. Louse-borne relapsing fever in southern Sudan. Transactions of the Royal Society of Tropical Medicine and Hygiene. 1995;89(6):621. PubMed PMID: 26016342.

45. Cuevas LE, Borgnolo G, Hailu B, Smith G, Almaviva M, Hart CA. Tumour necrosis factor, interleukin-6 and C-reactive protein in patients with louse-borne relapsing fever in Ethiopia. Annals of Tropical Medicine and Parasitology. 1995;89(1):49-54. PubMed PMID: 25151119.

46. Knox K, Fekade D, Hussein K, Melka A, Coxon R, Smith D, et al. Ovine polyclonal anti-TNF fab antibody suppresses Jarische-Herxheimer reaction of Louse-Borne relapsing fever. Abstracts of the Interscience Conference on Antimicrobial Agents and Chemotherapy. 1994;34(0):143. PubMed PMID: BIOSIS:PREV199598025505.

47. De Beer PAM. Louse-borne relapsing fever. Giulio Borgnolo et al. Tropical and Geographical Medicine 1993;45(2):66-9. Tropical and Geographical Medicine. 1994;46(3):192. PubMed PMID: 24220492.

48. Sundnes KO, Haimanot AT. Epidemic of louse-borne relapsing fever in Ethiopia.[Erratum appears in Lancet 1994 Jan 22;334(8891):244]. Lancet. 1993;342(8881):1213-5. PubMed PMID: 7901534.

49. Borgnolo G, Hailu B, Ciancarelli A, Almaviva M, Woldemariam T. Louse-borne relapsing fever. A clinical and an epidemiological study of 389 patients in Asella Hospital, Ethiopia. Trop Geogr Med. 1993;45(2):66-9. PubMed PMID: 8511813.

50. Borgnolo G, Denku B, Chiabrera F, Hailu B. Louse-borne relapsing fever in Ethiopian children: A clinical study. Annals of Tropical Paediatrics. 1993;13(2):165-71. PubMed PMID: 23193344.

51. Aknaviva M, Hailu B, Borgnolo G, Chiabrera F, Tolesse G, Gebre B. Louse-borne relapsing fever epidemic in Arssi Region, Ethiopia: A six months survey. Transactions of the Royal Society of Tropical Medicine and Hygiene. 1993;87(2):153. doi: <http://dx.doi.org/10.1016/0035-9203%2893%2990466-4>. PubMed PMID: 23136331.

52. Negussie Y, Remick DG, DeForge LE, Kunkel SL, Eynon A, Griffin GE. Detection of plasma tumor necrosis factor, interleuklns 6, and 8 during the jarisch-herxheimer reaction of relapsing fever. Journal of Experimental Medicine. 1992;175(5):1207-12. doi: 10.1084/jem.175.5.1207.

53. Mekasha A. Louse-borne relapsing fever in children. Journal of Tropical Medicine and Hygiene. 1992;95(3):206-9. PubMed PMID: 22183251.

54. Gebrehiwot T, Fiseha A. Tetracycline versus penicillin in the treatment of louse-borne relapsing fever. Ethiopian medical journal [Internet]. 1992; 30(3):[175-81 pp.]. Available from: <http://onlinelibrary.wiley.com/o/cochrane/clcentral/articles/444/CN-00087444/frame.html>.

55. Daniel E, Beyene H, Tessema T. Relapsing fever in children--demographic, social and clinical features. Ethiopian Medical Journal. 1992;30(4):207-14. PubMed PMID: 1459120.

56. Borgnolo G, Hailu B, Chiabrera F. Louse-borne relapsing fever in Ethiopia. Lancet. 1991;338(8770):827. PubMed PMID: 1681201.

57. Haider MJ, Zafar F, Khan KR. A SURVEY OF BORRELIA-RECURRENTIS IN HUMAN BLOOD IN KARACHI PAKISTAN. Karachi University Journal of Science. 1990;18(1-2):89-94. PubMed PMID: BIOSIS:PREV199293018028.

58. Brown V, Larouze B, Desve G, Rousset JJ, Thibon M, Fourrier A, et al. Clinical presentation of louse-borne relapsing fever among Ethiopian refugees in northern Somalia. Ann Trop Med Parasitol. 1988;82(5):499-502. PubMed PMID: 3257078.

59. Zein ZA. Louse borne relapsing fever (LBRF): Mortality and frequency of Jarisch-Herxheimer reaction. Journal of the Royal Society of Health. 1987;107(4):146-7. PubMed PMID: 17127609.

60. Isacsohn M, Gondard G, Yaoul E, Gindacu A, Kadosh D, Greenberg Z. LOUSE-BORNE RELAPSING FEVER IN ETHIOPIAN IMMIGRANTS. Israel Journal of Medical Sciences. 1985;21(8):710. PubMed PMID: BIOSIS:PREV198630047889.

61. Warrell DA, Perine PL, Krause DW, Bing DH, MacDougal SJ. Pathophysiology and immunology of the Jarisch-Herxheimer-like reaction in louse-borne relapsing fever: Comparison of tetracycline and slow-release penicillin. Journal of Infectious Diseases. 1983;147(5):898-909. PubMed PMID: 13094242.

62. Teklu B, Habte-Michael A, Warrell DA, White NJ, Wright DJM. MEPTAZINOL DIMINISHES THE JARISCH HERXHEIMER REACTION OF RELAPSING FEVER. Lancet. 1983;1(8329):836-9. PubMed PMID: BIOSIS:PREV198376081118.

63. Perine PL, Teklu B. Antibiotic treatment of louse-borne relapsing fever in Ethiopia: A report of 377 cases. American Journal of Tropical Medicine and Hygiene. 1983;32(5):1096-100. PubMed PMID: 13012101.

64. Butler T, Aikawa M, Habte-Michael A, Wallace C. Phagocytosis of Borrelia recurrentis by blood polymorphonuclear leukocytes is enhanced by antibiotic treatment. Infection and Immunity. 1980;28(3):1009-13. PubMed PMID: 10122535.

65. Ahmed MAM, Wahab SMA, Malik MOA. Louse-borne relapsing fever in the Sudan. A historical review and a clinico-pathological study. Tropical and Geographical Medicine. 1980;32(2):106-11. PubMed PMID: 11250204.

66. Butler T, Hazen P, Wallace CK. Infection with Borrelia recurrentis: Pathogenesis of fever and petechiae. Journal of Infectious Diseases. 1979;140(5):665-75. PubMed PMID: 10214189.

67. Butler T, Jones PK, Wallace CK. Borrelia recurrentis infection: single-dose antibiotic regimens and management of the Jarisch-Herxheimer reaction. Journal of Infectious Diseases. 1978;137(5):573-7. PubMed PMID: 8331814.

68. Salih SY, Mustafa D, Abdel Wahab SM, Ahmed MA, Omer A. Louse-borne relapsing fever: I. A clinical and laboratory study of 363 cases in the Sudan. Trans R Soc Trop Med Hyg. 1977;71(1):43-8. PubMed PMID: 871032.

69. Ormsbee R, Peacock M, Philip R, Casper E, Plorde J, Gabre-Kidan T, et al. Serologic diagnosis of epidemic typhus fever. American journal of epidemiology. 1977;105(3):261-71. Epub 1977/03/01. PubMed PMID: 403761.

70. Galloway RE, Levin J, Butler T, Naff GB, Goldsmith GH, Saito H, et al. Activation of protein mediators of inflammation and evidence for endotoxemia in Borrelia recurrentis infection. American Journal of Medicine. 1977;63(6):933-8. PubMed PMID: 8251835.

71. Dennis DT, Awoke S, Doberstyn EB, Fresh JW. Bleeding in louse borne relapsing fever in Ethopia. Clinical and laboratory features in 29 patients. East African Medical Journal. 1976;53(4):220-5. PubMed PMID: 7139090.

72. Perine PL, Reynolds DF. RELAPSING-FEVER EPIDEMIC IN THE SUDAN AND ETHIOPIA. Lancet. 1974;304(7892):1324-5. doi: 10.1016/S0140-6736(74)90189-5.

73. Perine PL, Krause DW, Awoke S, McDade JE. Single dose doxycycline treatment of louse borne relapsing fever and epidemic typhus. Lancet. 1974;2(7883):742-4. PubMed PMID: 5111608.

74. Knaack RH, Wright LJ, Leithead CS, Kidan TG, Plorde JJ. Penicillin vs. tetracycline in the treatment of louse borne relapsing fever. A preliminary report. Ethiopian Medical Journal. 1972;10(1):15-22. PubMed PMID: 4035835.

75. Bryceson AD, Cooper KE, Warrell DA, Perine PL, Parry EH. Studies of the mechanism of the Jarisch-Herxheimer reaction in louse-borne relapsing fever: evidence for the presence of circulating Borrelia endotoxin. Clinical science. 1972;43(3):343-54. PubMed PMID: 92522463.

76. Rijkels DF, Author A, Correspondence A, Rijkels DF. Louse-borne relapsing fever in Ethiopia. Tropical and Geographical Medicine. 1971;23(4):335-40.

77. Perine PL, Parry EH, Vukotich D, Warrell DA, Bryceson AD. Bleeding in louse-borne relapsing fever. I. Clinical studies in 37 patients. Transactions of the Royal Society of Tropical Medicine and Hygiene. 1971;65(6):776-81. PubMed PMID: 92372178.

78. Perine PL, Kidan TG, Warrell DA, Bryceson AD, Parry EH. Bleeding in louse-borne relapsing fever. II. Fibrinolysis following treatment. Transactions of the Royal Society of Tropical Medicine and Hygiene. 1971;65(6):782-7. PubMed PMID: 92372179.

79. Warrell DA, Pope HM, Parry EH, Perine PL, Bryceson AD. Cardiorespiratory disturbances associated with infective fever in man: studies of Ethiopian louse-borne relapsing fever. Clinical science. 1970;39(1):123-45. PubMed PMID: 90429807.

80. Bryceson ADM, Parry EHO, Perine PL, Warrell DA, Vukotich D, Leithead CS. A clinical and laboratory study of 62 cases in ethiopia and a reconsideration of the literature1. QJM. 1970;39(1):129-70. doi: 10.1093/oxfordjournals.qjmed.a067198.

81. Abdalla RE. Some studies on relapsing fever in the Sudan. The Journal of tropical medicine and hygiene. 1969;72(5):125-8. PubMed PMID: 89111553.

82. Schofield TP, Talbot JM, Bryceson AD, Parry EH. Leucopenia and fever in the "Jarisch-Herxheimer" reaction of louse-borne relapsing fever. Lancet. 1968;1(7533):58-62. PubMed PMID: 88019294.

83. Ombati DG, Ojiambo HP. Louse-borne relapsing fever. East African medical journal. 1968;45(9):630-1. PubMed PMID: 89018323.

84. Parry EH, Bryceson AD, Leithead CS. Acute hemodynamic changes during treatment of louse-borne relapsing fever. Lancet. 1967;1(7481):81-3. PubMed PMID: 87050972.

85. Cochran S. The Story of Hope Hospital*. Bull N Y Acad Med. 1961;37(1):47-65. PubMed PMID: 13694213; PubMed Central PMCID: PMC1804639.

86. Anderson TR, Zimmerman LE. RELAPSING FEVER IN KOREA - A CLINICOPATHOLOGIC STUDY OF 11 FATAL CASES WITH SPECIAL ATTENTION TO ASSOCIATION WITH SALMONELLA INFECTIONS. American Journal of Pathology. 1955;31(6):1083-109. PubMed PMID: WOS:A1955WD46500007.

87. Hirschboeck MM. THE USE OF CHLORAMPHENICOL IN RELAPSING FEVER. American Journal of Tropical Medicine and Hygiene. 1954;3(4):712-3. PubMed PMID: WOS:A1954UY65900016.

88. Zimmerman LE. Some Experiences with Enteric Diseases in Korea *†: Perforation of Paratyphoid Ulcers and Salmonella Septicemia Complicating Relapsing Fever. Am J Public Health Nations Health. 1953;43(3):279-84. PubMed PMID: 13030878; PubMed Central PMCID: PMC1620059.

89. Tadić RM. An epidemiological investigation of a rural district in Yugoslavia*. Bull World Health Organ. 1952;7(4):431-44. PubMed PMID: 13032787; PubMed Central PMCID: PMC2554137.

90. Harrison IB, Whittington RM. Antibiotics in the treatment of relapsing fever. United States Armed Forces medical journal. 1951;2(12):1859-62. PubMed PMID: MEDLINE:14884341.

91. Legerton CW, Chambers WL. Spontaneous rupture of the spleen in relapsing fever. United States Armed Forces medical journal. 1950;1(1):88-90. PubMed PMID: MEDLINE:15404480.

92. Gaud M, Bey MK, Vaucel M. The Evolution of the Epidemic of Relapsing Fever, 1942-1946. Bull World Health Organ. 1948;1(1):93-101. PubMed PMID: 20603923; PubMed Central PMCID: PMC2556134.

93. Bodman R, Steward I. Louse-borne relapsing fever in Persia. Brit Med J [Internet]. 1948; 4545:[291-3 pp.]. Available from: <http://onlinelibrary.wiley.com/o/cochrane/clcentral/articles/384/CN-00608384/frame.html>.

94. Garnham PC, Davies CW. An epidemic of louse-borne relapsing fever in Kenya. Transactions of the Royal Society of Tropical Medicine and Hygiene. 1947;41(1):141-70. PubMed PMID: 608181014.

95. Ingraham HS, Lapenta RG, Author A, Correspondence A, Ingraham HS. Penicillin in the treatment of louse-borne relapsing fever. United States naval medical bulletin. 1946;46(11):1719-23. PubMed Central PMCID: PMC21002713.

96. Chafic H, Atallah S, Author A, Correspondence A, Chafic H. Louse born relapsing fever in Haifa. Journal Palestine Arab Medical Association. 1946;2(1):8-13. PubMed Central PMCID: PMC20250218.

97. Wolman M. Louse-borne relapsing fever treated with calcium gold keratinate. Lancet (London, England). 1945;2(6381):775-7. PubMed PMID: 608087033.

98. Greaves FC, Gezon HM, Alston WF, Author A, Correspondence A, Greaves FC. Studies on louse-borne relapsing fever in Tunisia. United States naval medical bulletin. 1945;45:1029-48. PubMed Central PMCID: PMC21004211.

99. Robinson P. Relapsing Fever in Addis Ababa. Br Med J. 1942;2(4259):216-7. PubMed PMID: 20784396; PubMed Central PMCID: PMC2164011.

100. Charters AD. Relapsing fever in Abyssinia. Transactions of the Royal Society of Tropical Medicine and Hygiene. 1942;35(5):271-9. doi: 10.1016/S0035-9203(42)90045-2.

101. Mallannah S. Relapsing Fever in Raichur. Ind Med Gaz. 1923;58(4):168. PubMed Central PMCID: PMC5178439.

102. Sinton JA. Relapsing Fever at Meshed, North-East Persia. Ind Med Gaz. 1921;56(7):241-50. PubMed Central PMCID: PMC5166278.

103. Newcomb C. On an Outbreak of Relapsing Fever in Turkey in 1918. Ind Med Gaz. 1920;55(6):208-17. PubMed Central PMCID: PMC5180621.

104. Fry AS. An Epidemic of Fifty-Four Cases of Relapsing Fever Observed in Birjand, East Persia. Ind Med Gaz. 1920;55(1):2-8. PubMed Central PMCID: PMC5180769.

105. Stott H. On Two Varieties of Relapsing Fever Spirochætal Infection in India. Ind Med Gaz. 1911;46(8):292-8. PubMed Central PMCID: PMC5171664.

106. Mackie FP. THE PART PLAYED BY PEDICULUS CORPORIS IN THE TRANSMISSION OF RELAPSING FEVER. Br Med J. 1907;2(2450):1706-9. PubMed PMID: 20763589; PubMed Central PMCID: PMC2358735.

107. Amanzougaghene N, Akiana J, Mongo Ndombe G, Davoust B, Nsana NS, Parra HJ, et al. Head Lice of Pygmies Reveal the Presence of Relapsing Fever Borreliae in the Republic of Congo. PLoS Neglected Tropical Diseases. 2016;10 (12) (no pagination)(e0005142). doi: <http://dx.doi.org/10.1371/journal.pntd.0005142>. PubMed PMID: 613987217.

108. Boutellis A, Mediannikov O, Bilcha KD, Ali J, Campelo D, Barker SC, et al. Borrelia recurrentis in head lice, Ethiopia. Emerging Infectious Diseases. 2013;19(5):796-8. doi: <http://dx.doi.org/10.3201/eid1905.121480>. PubMed PMID: 368809123.

109. Brouqui P, Stein A, Dupont HT, Gallian P, Badiaga S, Rolain JM, et al. Ectoparasitism and vector-borne diseases in 930 homeless people from Marseilles. Medicine. 2005;84(1):61-8. doi: <http://dx.doi.org/10.1097/01.md.0000152373.07500.6e>. PubMed PMID: 40130185.

110. Raoult D, Birtles RJ, Montoya M, Perez E, Tissot-Dupont H, Roux V, et al. Survey of three bacterial louse-associated diseases among rural Andean communities in Peru: Prevalence of epidemic typhus, trench fever, and relapsing fever. Clinical Infectious Diseases. 1999;29(2):434-6. PubMed PMID: 29380213.

111. Ali M. Relapsing Fever *Paper read at the All-India Sub-Assistant Surgeons' onference at Agra. Ind Med Gaz. 1918;53(5):178-80. PubMed Central PMCID: PMC5201680.

112. Conseil E, Bienassis E. Traitement de la fièvre récurrente par le Néosalvarsan d'Ehrlich. Bull Soc Pathol Exot. 1912;7:476.

113. Hermant. Note sur la fièvre récurrente dans la province de Nghê-An. Bull Soc Med Chir Indochine. 1912;3:427.

114. Jukes AM. Preliminary note on some cases of Spirillar Fever in the Darjeeling district. Indian Med Gaz. 1912;47:476-7.

115. Mouzels P, Nguyen XM. Note sur 373 cas du fièvre récurrente traités au Lazaret de Hanoi par le 606 au cours de l'année 1912 de 1er janvier au 1er juin. Bulletin de la Société Médico-Chirurgicale de l'Indochine. 1912;3:427.

116. Sterling-Okuniewski S. Der Blutdruck im Verlaufe von Rückfallfieber. Cbl Bact Abt I Orig. 1918;82:456.

117. Jouveau-Dubreuil H. Formule leukocytaire et diminution des éosinophiles dans la fièvre récurrente. Bull Soc Pathol Exot. 1919;12:621.

118. Margolis A. Beobachtungen über Rückfallfieber. Beitr Klin Infektkrk. 1919;7:254.

119. Toyoda H. Studien über die Recurrensspirochäten in Mandschurien. Archives of Experimental Medicine. 1919;3:42.

120. Jouveau-Dubreuil H. Etude clinique sur la fièvre récurrente du Setchouen (Chine occidentale). Bull Soc Pathol Exot. 1920;13:38.

121. Prado Ed. Estudio del tifus recurrente en el Perú. Ann Fac Med Lima. 1920;3:26-45 and 134-52.

122. Sergent E, Foley H. Fièvre récurrente et ictère. Bull Soc Pathol Exot. 1921;14:632.

123. Oettinger J, Halbreich J. Ueber das Vorkommen einer ephemeren Roseola beim Rückfallfieber. Münch Med Woch. 1922;69:788.

124. Levy GS. Typhus recurrens im Kindesalter. Zeitschr Kinderh. 1926;42:627.

125. Chung HL. Presence of spirochetes in Urine and Prostatic Fluid of patients with Relapsing Fever. Proceedings of the Society for Experimental Biology and Medicine. 1938;38:97-8.

126. Böger A. Die latente Rekurrensinfektion und das Rekurrensrheumatoid. Münch Med Woch. 1943;90:549.

127. Robinson P. Typhus Fever in Addis Ababa. Ann Trop Med Parasit. 1943;37:38.

128. Chakrabarty A. Relapsing Fever (Louse borne) in north-east Bengal. J Indian Med Assoc. 1948;18:352-3.

129. Darwish AE. The Effect of Penicillin on the Behaviour of Spirochaetes in Natural and Inoculated Relapsing Fever. J Egypt Med Assoc. 1949;32:605-9.

130. Ravina A, Pêcher Y, Avril J. Typhus récurrente contracté à Paris. Bull et Mém Soc Med Hopt de Paris. 1950;11-12:508-10.

131. Smirnoff PP. Die Anwendung des Salvarsans bei Febris recurrens. Dtsch med Wochenschr. 1912;38(16):748-9. doi: 10.1055/s-0029-1189447.

132. Millous. Les Epidémies Fébriles non classées et la Fièvre Récurrente dans le Thanh-hoa en 1910, 1911, et 1912. . Bulletin de la Societe Medico-Chirurgicale de l'Indochine. 1913;4(1):14-8 pp.

133. Conseil E. Le Galyl et le Ludyl dans le Traitement de la Fièvre Récurrente. Bulletin de la Société de Pathologie Exotique. 1914;7(2):101-5 pp.

134. Foley H, Vialatte C. Traitement de la Fièvre Récurrente Nord-Africaine par le Néosalvarsan et I'Olarsol. . Bulletin de la Société de Pathologie Exotique. 1914;7(7):569-71 pp.

135. Babes V. Hémorragies méningées et autres manifestations hémorragiques dans la fièvre récurrente. OR Sianc Soc Biol (Paris). 1916;79:855.

136. Duchamp JC. Contribution to the Pathology of the Balkans: Serbian Spirochaeto-plasmodial Fever. Bulletin de la Société de Pathologie Exotique. 1917;10(9):827-34 pp.

137. Duchamp JC. La fievre recurrente chez les Serbes. Le progrès médical, Paris. 1917:10-3.

138. Dudgeon L. EXAMINATION OF THE URINE IN CASES OF RELAPSING FEVER OCCURRING IN MACEDONIA. The Lancet. 1917;190(4918):823-5. doi: <https://doi.org/10.1016/S0140-6736(01)56862-2>.

139. Hoesslin HV. Clinical picture of relapsing fever. Munchener Medizinische Wochenschrift. 1917;64(34):1106-9.

140. Kostoff KH. Arsal treatment in cases of relapsing fever. Dtsch Med Wochenschr. 1917;43(27-52):1168-9. doi: 10.1055/s-0028-1144668. PubMed PMID: WOS:000201475900124.

141. Porot A. Délire et réactions psychomotrices dans la fièvre récurrente de l'indigène. Bulletin de la Société de Pathologie Exotique. 1917;10(7):532-6 pp.

142. Portocalis A. Un cas d'insuffisance surrénale aiguë au cours de ia fièvre récurrente ; lésions préexistantes des capsules surrénales. Bulletin et Memoires de la Societe Medicale des Hopitaux de Paris. 1917;33(11-12):545-50 pp.

143. Tausig, Jurinac. A Case of Ruptured Spleen in R. F. Wiener Klinische Wochenschrift. 1917;30(52):1651 p.

144. Dumitresco M. Injections intraveineuses d'arrhénal dans la fièvre récurrente. Presse Medicale. 1918;26(17):155-6 pp.

145. Loewy R. The clinical picture and therapy of relapsing fever. Medizinische Klinik. 1918;14(3):62-3.

146. Portocalis A. The treatment of recurring fever. Comptes Rendus Seances Soc Biol Fil. 1918;81:273-4. PubMed PMID: WOS:000200953700111.

147. Sterling-Okuniewski S. Der Blutdruck im Verlaufe von Rückfallfieber. Dtsch med Wochenschr. 1918;44(10):265-6. doi: 10.1055/s-0028-1134308.

148. Calwell WK. RELAPSING FEVER:: AN ACCOUNT OF A SERIES OF 125 CASES, WITH SPECIAL REFERENCE TO THE PALESTINE TYPE. The Lancet. 1920;196(5068):785-8. doi: <https://doi.org/10.1016/S0140-6736(01)19765-5>.

149. Roy SC. Relapsing Fever Epidemic in Seoni District (Central Provinces), February to May, 1920. Indian Medical Gazette. 1921;56(1):7-9 pp.

150. Kerrest J, Gambier A, Bouron A. Recurrent fever in [French] Sudan. Bulletin de la Société de Pathologie Exotique. 1922;15(5):320-31.

151. Sergent E, Foley H. L'épidémiologie de la fiévre récurrente dans l'afrique du nord. Transactions of the Royal Society of Tropical Medicine and Hygiene. 1922;16(3):170-87. doi: <https://doi.org/10.1016/S0035-9203(22)90496-1>.

152. Selwyn-Clarke PS, Le Fanu GH, Ingram A. Relapsing Fever in. the Gold Coast. Annals of Tropical Medicine and Parasitology. 1923;17(3):389-426 pp.

153. Cunningham J. Serological Observations on Relapsing Fever in Madras. Transactions of the Royal Society of Tropical Medicine and Hygiene. 1925;19(1 and 2):11-33 pp.

154. McCuLloch WE. Relapsing Fever in Northern Nigeria-A Study of 300 Cases. Journal of Tropical Medicine and Hygiene. 1925;28(18):332-41 pp.

155. Beveridge GEG. The Louse-Borne Type of Relapsing Fever as Prevalent in the Anglo-Egyptian Sudan, 1926-and 1927. Medical Journal of Australia. 1928;1(4):110-2 pp.

156. Belezky WK, Umanskaja RM. Die Recurrensspirochätose des zentralen Nervensystems des Menschen. Z f d g Neur u Psych. 1930;129(1):21-41. doi: 10.1007/BF02865045.

157. Chu F-Ta, Deitrick S, Chung S-F. Relapsing Fever in Children. A Study of Twenty-Six Epidemic Cases. National Medical Journal of China. 1931;17(2):224-32 pp.

158. Russell H. Human and Experimental Relapsing Fever, Accra, Gold Coast, 1929-1930. West African Medical Journal. 1931;4(3):59-66 pp.

159. Robertson RC. Relapsing Fever in Shanghai (First Report). Chinese Medical Journal. 1932;46(9):853-85 pp.

160. Cunningham J, Fraser AGL. Further Observations on Indian Relapsing Fever. Part II. The Serology of Relapsing Fever in Human Beings. Indian Journal of Medical Research. 1935;22(4):595-616 pp.

161. Chung H-L. Studies on the Transmission of Relapsing Fever in North China. Preliminary Observations. Chinese Medical Journal. 1936;50(12):1723-34 pp.

162. Bruns A. On Relapsing Fever in Abyssinia. Archiv fur Schiffs- und Tropenhygiene. 1937;41(3):343-8 pp.

163. Chang SL. Relapsing fever in Changsha. A report of 41 cases. Chinese Med J. 1938;54:163-72.

164. Chung H-L. The Cerebrospinal Fluid of Patients suffering from the Chinese Strain of Relapsing Fever. Transactions of the Royal Society of Tropical Medicine and Hygiene. 1938;31(6):625-34 pp. doi: 10.1016/S0035-9203(38)90155-5.

165. Chung H-L, Wei Y-L. Studies on the Transmission of Relapsing Fever in North China II. Observations on the Mechanism of Transmission of Relapsing Fever in Man. The American Journal of Tropical Medicine and Hygiene. 1938;s1-18(6):661-74. doi: doi:<https://doi.org/10.4269/ajtmh.1938.s1-18.661>.

166. Chung H-L, Chang FC. Relapsing Fever. Clinical and Statistical Study of 337 Cases. Chinese Medical Journal. 1939;55(1):6-33 pp.

167. Wolman M. Observations on the Value of Treatment in Louse-borne Relapsing Fever. East African Medical Journal. 1944;21(11):336-40.

168. Benhamou E. Aspects actuels de la fièvre recurrente épidémique en Afrique du Nord. Bulletin de l'Academie de medecine. 1945;129(25-29):530-2. Epub 1945/01/01. PubMed PMID: 21008569.

169. El Ramley AH. Relapsing fever. Relapsing Fever Journal of the Egyptian Public Health Association. 1946;21:1-48.

170. Wolff BP. ASiatic relapsing fever; report of 134 cases treated with mapharsen*. Annals of Internal Medicine. 1946;24(2):203-16. doi: 10.7326/0003-4819-24-2-203.

171. Gaud M, Morgan M. Etude épidémiologique sur la fièvre récurrente en afrique du nord (1943-1945). Bull Wrld Hlth Org. 1947;1:75-98.

172. Shaul JF, Saferstein TH. Penicillin therapy in relapsing fever; report of four cases. United States naval medical bulletin. 1947;47(2):238-43. Epub 1947/03/01. PubMed PMID: 20288517.

173. Corkill NL. Activation of Latent Kala-Azar by Malaria and Relapsing Fever. Annals of Tropical Medicine & Parasitology. 1948;42(2):230-5. doi: 10.1080/00034983.1948.11685367.

174. Nasr LA. STERILE SPLENIC ABSCESS AFTER RELAPSING FEVER. Lancet. 1948;254(APR10):555-8. PubMed PMID: WOS:A1948UB99100004.

175. Senecal J, Ahmad A. [Treatment of recurrent fever with penicillin]. La semaine des hopitaux : organe fonde par l'Association d'enseignement medical des hopitaux de Paris. 1950;26(35):1634-8. Epub 1950/05/10. PubMed PMID: 15418271.

176. Vukotic D. Clinical and electrocardiographic findings of the heart in patients with louse-borne relapsing fever at the " day of crisis " and seven days later. Ethiopian medical journal. 1968;6(5):167-70.

177. Bolton BH, Anderson H, R K. Imported louse-borne relapsing fever -Ohio. Morbidity Mortality Weekly Rep. 1976.

178. Tewdros Eguale, Getahun Abate, Balcha F. Relapsing Fever in Hossana, Ethiopia: A Clinical and Epidemiological Study. Ethiopian Journal of Health Sciences. 2002;12(2):103-8.

179. Legesse MW, Gebre-Selassie S. Louse-Borne Relapsing Fever Profile at Jimma Hospital, Ethiopia: a retrospective study. Ethiopian Journal of Education and Sciences. 2005;1(1):60-4. doi: 10.4314/ejesc.v1i1.41987.

180. Ludlow AI. Relapsing fever in Korea. Ohio State Medical Journal. 1943;39:1011-3.

181. Nordmann T, Feldt T, Bosselmann M, Tufa TB, Lemma G, Holtfreter M, et al. Outbreak of Louse-Borne Relapsing Fever among Urban Dwellers in Arsi Zone, Central Ethiopia, from July to November 2016. American Journal of Tropical Medicine and Hygiene. 2018;98(6):1599-602. doi: 10.4269/ajtmh.17-0470. PubMed PMID: BIOSIS:PREV201800626499.

182. Alberer M, Malinowski S, Sanftenberg L, Schelling J. Notifiable infectious diseases in refugees and asylum seekers: experience from a major reception center in Munich, Germany. Infection. 2018;46(3):375-83. doi: 10.1007/s15010-018-1134-4.

183. Fasciana T, Calà C, Colomba C, Mascarella C, Scarlata F, Capra G, et al. A new case of louse-borne relapsing fever in sicily: Case report and mini review. Pharmacologyonline. 2017;1(Special Issue):62-6.

184. Antinori S, Tonello C, Edouard S, Parravicini C, Gastaldi D, Grande R, et al. Diagnosis of louse-borne relapsing fever despite negative microscopy in two asylum seekers from Eastern Africa. American Journal of Tropical Medicine and Hygiene. 2017;97(6):1669-72. doi: 10.4269/ajtmh.17-0320.

185. Mourembou G, Fenollar F, Socolovschi C, Lemamy GJ, Nzoughe H, Kouna LC, et al. Molecular Detection of Fastidious and Common Bacteria as well as Plasmodium spp. in Febrile and Afebrile Children in Franceville, Gabon. Am J Trop Med Hyg. 2015;92(5):926-32. doi: 10.4269/ajtmh.14-0699. PubMed PMID: 25802432; PubMed Central PMCID: PMC4426579.

186. Veena S, Seema V, Babu R. Borreliosis: Recurrent fever due to spirochetes. Annals of Tropical Medicine and Public Health. 2013;6(4):482-4. doi: <http://dx.doi.org/10.4103/1755-6783.127806>. PubMed PMID: 372572237.

187. Cruz NS, Mayoral PV. Borreliosis: Recurrent fever due to spirochetes. Case report. Boletin Medico del Hospital Infantil de Mexico. 2012;69(2):114-8. PubMed PMID: 365462124.

188. Clemens EG, Schachterle S, Reller ME, Mtove G, Sullivan D, Dumler J. Multiplex 5 ' Nuclease qPCR for the Diagnosis of Relapsing Fever in Large Clinical Cohorts. Abstracts of the General Meeting of the American Society for Microbiology. 2010;110:Y-1494. PubMed PMID: BIOSIS:PREV201500674284.

189. Animut A, Mekonnen Y, Shimelis D, Ephraim E. Febrile Illnesses of Different Etiology among Outpatients in Four Health Centers in Northwestern Ethiopia. Japanese Journal of Infectious Diseases. 2009;62(2):107-10. PubMed PMID: WOS:000265074600004.

190. Aher A, Shah H, Rastogi V, Tukaram P, Choudhury R. A case report of relapsing fever. Indian Journal of Pathology and Microbiology. 2008;51(2):292-3. doi: <http://dx.doi.org/10.4103/0377-4929.41703>. PubMed PMID: 351961390.

191. Gallien S, Sarfati C, Haas L, Lagrange-Xelot M, Molina JM. Borreliosis: A rare and alternative diagnosis in travellers' febrile illness. Travel Medicine and Infectious Disease. 2007;5(4):247-50. doi: 10.1016/j.tmaid.2007.01.002.

192. Okwori EE. Spirochaetemia in a HIV positive patient. Nigerian journal of medicine : journal of the National Association of Resident Doctors of Nigeria. 2006;15(4):455-6. PubMed PMID: 44985704.

193. Niren AS, Sundararaj T, Jayapal V, Subramanian S. Studies on the reemerging Borrelial infection in man. Biomedicine. 2000;20(4):236-42.

194. Westenfeld FW. Clinical pathology rounds: Relapsing fever in a recent visitor to Africa. LAB MED. 1997;28(7):436-8.

195. Poulsen LW, Iversen G. Relapsing fever: A differential diagnosis to Malaria. Scandinavian Journal of Infectious Diseases. 1996;28(4):419-20. doi: 10.3109/00365549609037932. PubMed PMID: WOS:A1996VH12100019.

196. Raz R, Sharir R, Bennett M. Prevention of Borrelia recurrentis infection with tetracycline. Israel journal of medical sciences. 1987;23(3):221. PubMed PMID: 17749343.

197. Galun E, Ben-Chetrit E. Possible prevention of tick-borne relapsing fever in patients infected with Borrelia recurrentis. The Journal of infectious diseases. 1984;150(4):617. PubMed PMID: 14807260.

198. Rosenthal E. Relapsing fever in Cape Town. A case report. South African Medical Journal. 1982;61(21):801-2. PubMed PMID: 12086365.

199. Karimi Y, Hannoun C, Ardoin P, Ameli M, Mohallati HB. Sur le purpura hémorragique observé dans l'Azarbaidjan-Est de l'Iran. Med Mal Infect. 1976;6(10 PART 2):399-404. doi: 10.1016/S0399-077X(76)80124-2.

200. Klein M. Relapsing Fever—Successful Treatment with Demethylchlortetracycline (Declomycin®). Calif Med. 1964;100(4):283-5. PubMed PMID: 14165878; PubMed Central PMCID: PMC1515525.

201. Coghill NF, Lawrence J, Ballantine ID. Relapsing Fever in Cyrenaica. Br Med J. 1947;1(4505):637-40. PubMed PMID: 20248076; PubMed Central PMCID: PMC2053195.

202. Hamilton JB. OCULAR COMPLICATIONS IN RELAPSING FEVER*. Br J Ophthalmol. 1943;27(2):68-80. PubMed PMID: 18169904; PubMed Central PMCID: PMC510307.

203. L. VH. Note préliminaire sur la fièvre récurrente parmi les troupes belges dans l'Est Africain Allemand. Bull Soc Path Exot Paris. 1917:786-91.

204. B. CA. Notes from Kordofan on two cases of fever associated with spirochaetes in the blood. Roy Army Med Corps. 1910;14:99.

205. E. RH. The Wassermann Reaction in Relapsing Fever. Brit J Exper Path. 1922;59:3.

206. J.M. G. Tratamiento de la fiebre recurrente por medio de la penicilina. Rev Facul de Med. 1946;16:264-5.

207. R. S, D. M, I. R. Estudio clinico, hematologico y terapeutico de un brote de fiebre recurrente. Med Colon. 1947;9:207.

208. Fotso AF, Angelakis E, Mouffok N, Drancourt M, Raoult D. Blood-borne candidatus borrelia algerica in a patient with prolonged fever in oran, Algeria. American Journal of Tropical Medicine and Hygiene. 2015;93(5):1070-3. doi: <http://dx.doi.org/10.4269/ajtmh.15-0124>. PubMed PMID: 606855824.

209. Halperin T, Orr N, Cohen R, Hasin T, Davidovitch N, Klement E, et al. Detection of relapsing fever in human blood samples from Israel using PCR targeting the glycerophosphodiester phosphodiesterase (GlpQ) gene. Acta Tropica. 2006;98(2):189-95. doi: <http://dx.doi.org/10.1016/j.actatropica.2006.04.004>. PubMed PMID: 43796265.

210. Dworkin MS, Schwan TG, Anderson Jr DE. Tick-borne relapsing fever in North America. Medical Clinics of North America. 2002;86(2):417-33. doi: <http://dx.doi.org/10.1016/S0025-7125%2803%2900095-6>. PubMed PMID: 34327095.

211. Melkert PWJ. Relapsing fever in pregnancy: analysis of high‐risk factors. BJOG Int J Obstet Gynaecol. 1988;95(10):1070-2. doi: 10.1111/j.1471-0528.1988.tb06516.x.

212. Smith L, Brown TG. Relapsing fever--a case history. Calif Med. 1969;110(4):322-4. PubMed PMID: 5798503; PubMed Central PMCID: PMC1503490.

213. van Tongeren HA, Koetsier JC, Author A, Correspondence A, Tongeren HAv. Encephalitis following an infection with Borrelia recurentis var. Duttoni ORIGINAL (NON-ENGLISH) TITLE Encephalitis ten gevolge van een besmetting met Borrelia recurrentis var. Duttoni. Nederlands tijdschrift voor geneeskunde. 1968;112(40):1778-81. PubMed Central PMCID: PMC5680834.

214. Mazzotti L, Author A, Correspondence A, Mazzotti L. Presence of Borrelia recurrentis in Ornithodoros talaje in Mexico

Presencia en México, de espiroquetas de la fiebre recurrente en Ornithodoros talaje. Revista del Instituto de Salubridad y Enfermedades Tropicales. 1953;13(4):285-7. PubMed Central PMCID: PMC13178235.

215. Parsons L. RELAPSING FEVER AT LAKE TAHOE, CALIFORNIA-NEVADA. American Journal of Clinical Pathology. 1947;17(5):388-92. PubMed PMID: WOS:A1947UE17400005.

216. H. HCH. Tick Fever in East Persia. J Roy Army Med Corps. 1922;38:398.

217. R. PM. Caractéristiques d'une souche de Borrelia Recurrentis isolée au Portugal. Arq Inst Bacteriol Camara Pestana. 1945;9:224.

218. K. MEM. Penicillin in treatment of Relapsing Fever. East African Med J. 1946;23:55-64.

219. Merskey. Relapsing fever in Cullinan (Tansvaal) with a short reference to penicillin therapy. Clin Proc Capetown. 1947;6:113.

220. W.S. G. Aureomycin and Relapsing Fever. Lancet. 1950;268:515.

221. I. I. Aureomycin in Relapsing Fever. N Y State J Med. 1951;61:1057.

222. De R. La fièvre récurrente spirillaire et son traitement aux troupes de l'Est Africain Allemand. Arch Med Belges. 1917;70(8):710-3 pp.

223. Manson JK, Thornton LHD. East African Relapsing Fever. Journal of the Royal Army Medical Corps. 1919;33(3):193-216.

224. Bergsma S. Relapsing Fever in Abyssinia. Journal of Tropical Medicine and Hygiene. 1928;31(22):289-90 pp.

225. Bodley Scott R. NEUROLOGICAL COMPLICATIONS OF RELAPSING FEVER. The Lancet. 1944;244(6318):436-8. doi: <https://doi.org/10.1016/S0140-6736(00)45267-0>.

226. Taft WC, Pike JB. Relapsing Fever. Report of a Sporadic Outbreak, including Treatment with Penicillin. Journal of the American Medical Association. 1945;129(15):1002-5.

227. Calabi O. The presence of plasma inhibitors during the crisis phenomenon in experimental relapsing fever (Borrelia novyi). The Journal of experimental medicine. 1959;110:811-25. Epub 1959/11/01. PubMed PMID: 13848161; PubMed Central PMCID: PMCPMC2137020.

228. Coffey EM, Eveland WC. Experimental relapsing fever initiated by Borella hermsi. I. Identification of major serotypes by immunofluorescence. The Journal of infectious diseases. 1967;117(1):23-8. Epub 1967/02/01. PubMed PMID: 5338693.

229. Melkert PW. Fatal-Jarisch Herxheimer reaction in a case of relapsing fever misdiagnosed as lobar pneumonia. Tropical and geographical medicine. 1987;39(1):92-3. Epub 1987/01/01. PubMed PMID: 3603698.

230. Melkert PW. Relapsing fever in pregnancy: analysis of high-risk factors. British journal of obstetrics and gynaecology. 1988;95(10):1070-2. Epub 1988/10/01. PubMed PMID: 3191046.

231. Melkert PW, Stel HV. Neonatal Borrelia infections (relapsing fever): report of 5 cases and review of the literature. East Afr Med J. 1991;68(12):999-1005. Epub 1991/12/01. PubMed PMID: 1800101.

232. Hamoud B, Jean David P-G-C, Danielle P, Guy B, Ronan J. Identifying Relapsing Fever <em>Borrelia</em>, Senegal. Emerging Infectious Disease journal. 2005;11(3):474. doi: 10.3201/eid1103.040506.

233. Cadavid D, Sondey M, Garcia E, L Lawson C. Residual Brain Infection in Relapsing‐Fever Borreliosis. J Infect Dis 2006;193:1451-8. doi: 10.1086/503367.

234. Halperin T, Orr N, Cohen R, Hasin T, Davidovitch N, Klement E, et al. Detection of relapsing fever in human blood samples from Israel using PCR targeting the glycerophosphodiester phosphodiesterase (GlpQ) gene. Acta tropica. 2006;98(2):189-95. Epub 2006/05/30. doi: 10.1016/j.actatropica.2006.04.004. PubMed PMID: 16729949.

235. Larsson C, Andersson M, Guo BP, Nordstrand A, Hägerstrand I, Carlsson S, et al. Complications of Pregnancy and Transplacental Transmission of Relapsing-Fever Borreliosis. The Journal of infectious diseases. 2006;194(10):1367-74. doi: 10.1086/508425.

236. Larsson C, Andersson M, Pelkonen J, Guo BP, Nordstrand A, Bergström S. Persistent brain infection and disease reactivation in relapsing fever borreliosis. Microbes Infect. 2006;8(8):2213-9. doi: 10.1016/j.micinf.2006.04.007.

237. Andersson M, Nordstrand A, Shamaei-Tousi A, Jansson A, Bergström S, Guo BP. In situ immune response in brain and kidney during early relapsing fever borreliosis. Journal of Neuroimmunology. 2007;183(1):26-32. doi: <https://doi.org/10.1016/j.jneuroim.2006.11.004>.

238. Naddaf SR, Ghazinezhad B, Kazemirad E, Cutler SJ. Relapsing fever causative agent in Southern Iran is a closely related species to East African borreliae. Ticks and Tick-borne Diseases. 2017;8(6):882-6. doi: <http://dx.doi.org/10.1016/j.ttbdis.2017.07.006>.

239. Masuda G. Relapsing fever. [Japanese]. Nippon rinsho. 2007;Japanese journal of clinical medicine. 65 Suppl 3:192-5. PubMed PMID: 46914557.

240. Sirnes KE, Author A, Med. Avd SSS, Correspondence A, Med. Avd SSS. Relapsing fever. Tidsskrift for den Norske Laegeforening. 1982;102(32):1710-1+21. PubMed PMID: 1983042645; PubMed Central PMCID: PMC7167942.

241. van der Heide RM. [A case of recurrent fever imported into the Netherlands]. Nederlands tijdschrift voor geneeskunde. 1971;115(14):607-8. Epub 1971/04/03. PubMed PMID: 5563048.

242. Levin MI. [Nonspecific forms of European recurrent typhus]. Klin Med (Mosk). 1950;28(7):79-80. PubMed PMID: 15437696; PubMed Central PMCID: PMCSource: CLML. 5019:28361:205.

243. Tylewska-Wierzbanowska S, Fiecek B, Chmielewski T. Relapsing fevers. Postepy Mikrobiologii. 2018;51(1):41-6.

244. Avanessov. in Russian. Med Parazit Parazit Bolez. 1938.

245. A. L, G. T. Febbre Ricorrente in Eritrea. Arch Ital Sci Med Trop. 1961;42:434.

246. Van der Heide RM. A Case of Recurrent Fever Imported into the Netherlands. Ned Tijdschr Geneeskd 1971;14:607-8.

247. D'Ignazio C, Codeleoncini E. Pneumonic Form of Relapsing Fever and of Typhus. Bollettino della Societa Italiana di Medicina e Igiene Tropicale. 1945;5(5/6):211-20.

248. Wang G. Borrelia burgdorferi and Other Borrelia Species. Molecular Medical Microbiology: Second Edition. 3: Elsevier Ltd; 2014. p. 1867-909.

249. Olmo Montes FJ, Sojo Dorado J, Peñas Espinar C, Muniáin Ezcurra MA. Borrelia species: Lyme disease and relapsing fever. Medicine. 2014;11(51):3009-17. doi: 10.1016/S0304-5412(14)70731-0.

250. Horton JM. Relapsing Fever Caused by Borrelia Species. Mandell, Douglas, and Bennett's Principles and Practice of Infectious Diseases. 2: Elsevier Inc.; 2014. p. 2721-4.

251. Halperin JJ, García-Moncó JC. The human borreliosis: Lyme neuroborreliosis and relapsing fever. CNS Infections: A Clinical Approach: Springer-Verlag London Ltd; 2014. p. 211-26.

252. Diaz JH. Lice (Pediculosis). Mandell, Douglas, and Bennett's Principles and Practice of Infectious Diseases. 2: Elsevier Inc.; 2014. p. 3246-9.e1.

253. Stanke C, Kerac M, Prudhomme C, Medlock J, Murray V. Health Effects of Drought: a Systematic Review of the Evidence. PLoS Curr. 2013;5. doi: 10.1371/currents.dis.7a2cee9e980f91ad7697b570bcc4b004. PubMed PMID: 23787891; PubMed Central PMCID: PMC3682759.

254. Sassera D, Epis S, Pajoro M, Bandi C. Microbial symbiosis and the control of vectorborne pathogens in tsetse flies, human lice, and triatomine bugs. Pathog Global Health. 2013;107(6):285-92. doi: 10.1179/2047773213Y.0000000109.

255. Ncbi. Borrelia recurrentis overview. European Nucleotide Archive. 2013. PubMed PMID: DRCI:DATA2013106003824898.

256. Izri A, Guiguen C. Pediculosis and laboratory role. Rev Fr Lab. 2013;2013(454):33-9. doi: 10.1016/S1773-035X(13)72129-4.

257. Inci A, Yazar S, Tuncbilek AS, Canhilal R, Doganay M, Aydin L, et al. Vectors and Vector-Borne Diseases in Turkey. Ankara Universitesi Veteriner Fakultesi Dergisi. 2013;60(4):281-96. doi: 10.1501/Vetfak_0000002593. PubMed PMID: WOS:000334986500010.

258. Elbir H, Raoult D, Drancourt M. Review article: Relapsing fever borreliae in Africa. American Journal of Tropical Medicine and Hygiene. 2013;89(2):288-92. doi: <http://dx.doi.org/10.4269/ajtmh.12-0691>. PubMed PMID: 369561715.

259. Elbir H, Henry M, Diatta G, Mediannikov O, Sokhna C, Tall A, et al. Multiplex Real-Time PCR Diagnostic of Relapsing Fevers in Africa. PLoS Neglected Tropical Diseases. 2013;7 (1) (no pagination)(e2042). doi: <http://dx.doi.org/10.1371/journal.pntd.0002042>. PubMed PMID: 368294238.

260. Bonilla DL, Durden LA, Eremeeva ME, Dasch GA. The Biology and Taxonomy of Head and Body Lice-Implications for Louse-Borne Disease Prevention. PLoS Pathogens. 2013;9 (11) (no pagination)(e1003724). doi: <http://dx.doi.org/10.1371/journal.ppat.1003724>. PubMed PMID: 370341873.

261. Anonymous. The human body louse is a vector for bacterial agents that cause louse-borne relapsing fever, trench fever, and epidemic typhus. European Nucleotide Archive. 2013. PubMed PMID: DRCI:DATA2013106003815019.

262. Schaub GA, Kollien AH, Balczun C. Lice as Vectors of Bacterial Diseases. Mehlhorn H, editor2012. 255-74 p.

263. Irwin JF. The Great White Train: typhus, sanitation, and U.S. International Development during the Russian Civil War. Endeavour. 2012;36(3):89-96. doi: 10.1016/j.endeavour.2012.03.001. PubMed PMID: BIOSIS:PREV201200713029.

264. Embers ME, Lopez JE. Immune resistance by relapsing fever spirochetes. The Pathogenic Spirochetes: Strategies for Evasion of Host Immunity and Persistence. 9781461454045: Springer US; 2012. p. 173-91.

265. Elbir H, Gimenez G, Sokhna C, Bilcha KD, Ali J, Barker SC, et al. Multispacer sequence typing relapsing fever Borreliae in Africa.[Erratum appears in PLoS Negl Trop Dis. 2012 Jun;6(6). doi:10.1371/annotation/5b575a3d-79c6-4450-9410-225e554da42d Note: Haitham, Elbir [corrected to Elbir, Haitham]]. PLoS Negl Trop Dis. 2012;6(6):e1652. doi: <https://dx.doi.org/10.1371/journal.pntd.0001652>. PubMed PMID: 22679518.

266. El-Bahnsawy MM, Labib NA, Abdel-Fattah MAH, Ibrahim AM, Morsy TA. Louse and tick borne relapsing fevers. Journal of the Egyptian Society of Parasitology. 2012;42(3):625-38. PubMed PMID: 603752645.

267. El-Bahnasawy MM, Abdel FE, Morsy TA. Human pediculosis: a critical health problem and what about nursing policy? Journal of the Egyptian Society of Parasitology. 2012;42(3):541-62. PubMed PMID: 603752455.

268. Drancourt M. Relapsing Fever and Borrelioses. Hunter's Tropical Medicine and Emerging Infectious Disease: Ninth Edition: Elsevier Inc.; 2012. p. 602-6.

269. Badiaga S, Brouqui P. Human louse-transmitted infectious diseases. Clinical Microbiology and Infection. 2012;18(4):332-7. doi: <http://dx.doi.org/10.1111/j.1469-0691.2012.03778.x>. PubMed PMID: 51882344.

270. Norris SJ, Lin T. Out of the Woods: The remarkable genomes of the genus Borrelia. Journal of Bacteriology. 2011;193(24):6812-4. doi: <http://dx.doi.org/10.1128/JB.06317-11>. PubMed PMID: 364093906.

271. Guerrier G, Doherty T. Comparison of antibiotic regimens for treating louse-borne relapsing fever: A meta-analysis. Transactions of the Royal Society of Tropical Medicine and Hygiene. 2011;105(9):483-90. doi: <http://dx.doi.org/10.1016/j.trstmh.2011.04.004>. PubMed PMID: 51548792.

272. Desenclos JC, Laporte A, Brouqui P. [Louse-borne infections in humans]. Med Mal Infect. 2011;41(6):295-300. doi: <https://dx.doi.org/10.1016/j.medmal.2011.02.003>. PubMed PMID: 21450425.

273. Cutler SJ, Rinky IJ, Bonilla EM. Does RecA have a role in Borrelia recurrentis? Clinical Microbiology and Infection. 2011;17(2):195-7. doi: <http://dx.doi.org/10.1111/j.1469-0691.2010.03249.x>. PubMed PMID: 361148138.

274. Cutler S. Spirochaetes: Past lessons to future directions. Clinical Microbiology and Infection. 2011;17(4):481-3. doi: 10.1111/j.1469-0691.2011.03485.x.

275. Brouqui P. Arthropod-borne diseases associated with political and social disorder. Annual Review of Entomology2011. p. 357-74.

276. Barbour AG. Relapsing Fever and other Borrelia Diseases. Tropical Infectious Diseases: Elsevier Inc.; 2011. p. 295-302.

277. Warrell DA. Relapsing fevers. Infectious Diseases: Third Edition. 2: Elsevier Inc.; 2010. p. 1243-6.

278. Stanek G. Borrelioses. Denisia. 2010;(30):605-24. PubMed PMID: BIOSIS:PREV201100601409.

279. Sidi G, Schwartz E. Relapsing Fever. Tropical Diseases in Travelers: Wiley-Blackwell; 2010. p. 169-74.

280. Schwan TG. Investigations of Relapsing Fever at Home and Abroad. St Georgiev V, Zoon KC, editors: Humana Press Inc, 999 Riverview Dr, Ste 208, Totowa, Nj 07512-1165 USA; 2010. 101-6 p.

281. Pages F, Faulde M, Orlandi-Pradines E, Parola P. The past and present threat of vector-borne diseases in deployed troops. Clinical Microbiology and Infection. 2010;16(3):209-24. doi: 10.1111/j.1469-0691.2009.03132.x.

282. Habedank B. Lice - Biology, medical importance and control. Denisia. 2010;(30):191-212. PubMed PMID: BIOSIS:PREV201100601387.

283. Grosskinsky S, Schott M, Brenner C, Cutler SJ, Simon MM, Wallich R. Human complement regulators C4b-binding protein and C1 esterase inhibitor interact with a novel outer surface protein of Borrelia recurrentis. PLoS Neglected Tropical Diseases. 2010;4 (6) (no pagination)(e698). doi: <http://dx.doi.org/10.1371/journal.pntd.0000698>. PubMed PMID: 359899958.

284. Cutler SJ, Margarita Bonilla E, Singh RJ. Population structure of East African relapsing fever Borrelia spp. Emerging Infectious Diseases. 2010;16(7):1076-80. doi: <http://dx.doi.org/10.3201/eid1607.091085>. PubMed PMID: 359145851.

285. Cutler SJ. Relapsing fever--a forgotten disease revealed. Journal of Applied Microbiology. 2010;108(4):1115-22. doi: <https://dx.doi.org/10.1111/j.1365-2672.2009.04598.x>. PubMed PMID: 19886891.

286. Anonymous. 4th International Conference on Phthiraptera, Urgup, TURKEY, June 13 -18, 2010. Turkiye Parazitoloji Dergisi. 2010;34(Suppl. 1):5. PubMed PMID: BIOSIS:PREV201000458388.

287. Larsson C, Lundqvist J, Van Rooijen N, Bergstrom S. A novel animal model of Borrelia recurrentis Louse-Borne relapsing fever borreliosis using immunodeficient mice. PLoS Neglected Tropical Diseases. 2009;3 (9) (no pagination)(E522). doi: <http://dx.doi.org/10.1371/journal.pntd.0000522>. PubMed PMID: 355629308.

288. Larsson C, Andersson M, Bergstrom S. Current issues in relapsing fever. Curr Opin Infect Dis. 2009;22(5):443-9. PubMed PMID: CCC:000270052300004.

289. Grosskinsky S, Schott M, Simon MM, Zipfel PF, Wallich R. Borrelia recurrentis interferes with human innate immune defenses through acquisition of complement regulators CFH, CFHR-1, C4b-binding protein and C1-INH. Molecular Immunology. 2009;46 (14):2833. doi: <http://dx.doi.org/10.1016/j.molimm.2009.05.229>. PubMed PMID: 70365528.

290. Grosskinsky S, Schott M, Brenner C, Cutler SJ, Kraiczy P, Zipfel PF, et al. Borrelia recurrentis employs a novel multifunctional surface protein with anti-complement, anti-opsonic and invasive potential to escape innate immunity. PLoS ONE. 2009;4 (3) (no pagination)(e4858). doi: <http://dx.doi.org/10.1371/journal.pone.0004858>. PubMed PMID: 354401001.

291. Cutler SJ, Abdissa A, Trape JF. New concepts for the old challenge of African relapsing fever borreliosis. Clinical Microbiology and Infection. 2009;15(5):400-6. doi: <http://dx.doi.org/10.1111/j.1469-0691.2009.02819.x>. PubMed PMID: 354616244.

292. Cutler SJ. Myths, legends and realities of relapsing fever borreliosis. Clinical Microbiology and Infection. 2009;15(5):395-6. doi: 10.1111/j.1469-0691.2009.02817.x.

293. Cutler S. Uncovering the secrets of East African relapsing fever. Clinical Microbiology and Infection. 2009;15:S261. doi: <http://dx.doi.org/10.1111/j.1469-0691.2009.02858.x>. PubMed PMID: 70070986.

294. Thein M, Bunikis I, Denker K, Larsson C, Cutler S, Drancourt M, et al. Oms38 is the first identified pore-forming protein in the outer membrane of relapsing fever spirochetes. Journal of Bacteriology. 2008;190(21):7035-42. doi: <http://dx.doi.org/10.1128/JB.00818-08>. PubMed PMID: 352643472.

295. Muñiz AE. Infestations. Pediatric Emergency Medicine: Elsevier Inc.; 2008. p. 871-9.

296. Marseille-Nice G. Causes louse-borne relapsing fever. European Nucleotide Archive. 2008. PubMed PMID: DRCI:DATA2015255007310032.

297. Lescot M, Audic S, Robert C, Nguyen TT, Blanc G, Cutler SJ, et al. The genome of Borrelia recurrentis, the agent of deadly louse-borne relapsing fever, is a degraded subset of tick-borne Borrelia duttonii. PLoS Genetics. 2008;4 (9) (no pagination)(e1000185). doi: <http://dx.doi.org/10.1371/journal.pgen.1000185>. PubMed PMID: 352440609.

298. Larsson C, Bergström S. A Novel and Simple Method for Laboratory Diagnosis of Relapsing Fever Borreliosis. Open Microbiol J. 2008;2:10-2. doi: 10.2174/1874285800802010010. PubMed PMID: 19088905; PubMed Central PMCID: PMC2593045.

299. LaRocca TJ, Katona LI, Thanassi DG, Benach JL. Bactericidal action of a complement-independent antibody against relapsing fever Borrelia resides in its variable region. J Immunol. 2008;180(9):6222-8.

300. Cutler SJ, Scott JC, Wright DJM. Phylogenetic origins of Borrelia recurrentis. International Journal of Medical Microbiology. 2008;298(SUPPL. 1):193-202. doi: <http://dx.doi.org/10.1016/j.ijmm.2007.05.005>. PubMed PMID: 50060781.

301. Couloux A, Nguyen TT, Cutler SJ, Drancourt M, Wincker P, Lescot M, et al. Borrelia gene families. Figshare. 2008. doi: <http://dx.doi.org/10.1371/journal.pgen.1000185.t002>. PubMed PMID: DRCI:DATA2013073003305449.

302. Badiaga S, Raoult D, Brouqui P. Preventing and Controlling Emerging and Reemerging Transmissible Diseases in the Homeless. Emerg Infect Dis. 2008;14(9):1353-9. doi: 10.3201/eid1409.082042. PubMed PMID: 18760000; PubMed Central PMCID: PMC2603102.

303. Argenbright R. Lethal mobilities: Bodies and lice on Soviet railroads, 1918-1922. J Transp Hist. 2008;29(2):259-76.

304. Infectious Diseases and Anthropods, 2nd Edition. Goddard J, editor: Humana Press Inc, 999 Riverview Dr, Ste 208, Totowa, Nj 07512-1165 USA; 2008.

305. Meri T, Pusa E, Cutler S, Jokiranta TS. A novel classical pathway evasion mechanism by relapsing fever Borreliae. Molecular Immunology. 2007;44(1-3, Sp. Iss. SI):215. doi: 10.1016/j.molimm.2006.07.159. PubMed PMID: BIOSIS:PREV200700146238.

306. Meng Y-f, Guo X-g, Wu D. Primary report on the checklist of sucking lice (Phthiraptera) in Yunnan Province. Entomotaxonomia. 2007;29(4):259-64. PubMed PMID: BIOSIS:PREV200800343235.

307. Reeves WK, Szumlas DE, Moriarity JR, Loftis AD, Abbassy MM, Helmy IM, et al. Louse-borne bacterial pathogens in lice (Phthiraptera) of rodents and cattle from Egypt. The Journal of parasitology. 2006;92(2):313-8. doi: <http://dx.doi.org/10.1645/GE-717R.1>. PubMed PMID: 43819074.

308. Meri T, Cutler SJ, Blom AM, Meri S, Jokiranta TS. Relapsing fever spirochetes Borrelia recurrentis and B. duttonii acquire complement regulators C4b-binding protein and factor H. Infection and Immunity. 2006;74(7):4157-63. doi: <http://dx.doi.org/10.1128/IAI.00007-06>. PubMed PMID: 43993349.

309. Humphreys M. A stranger to our camps: Typhus in American history. Bulletin of the History of Medicine. 2006;80(2):269-90. PubMed PMID: 44022289.

310. Gratz NG. The vector- and rodent-borne diseases of Europe and North America: Their distribution and public health burden: Cambridge University Press; 2006. 1-393 p.

311. Cutler SJ. Possibilities for relapsing fever reemergence. Emerging Infectious Diseases. 2006;12(3):369-74. PubMed PMID: 43306259.

312. Brouqui P, Raoult D. Arthropod-borne diseases in homeless. In: Hechemy KE, Blanco JR, Silverman DJ, Raoult DA, Oteo JA, editors. Annals of the New York Academy of Sciences2006. p. 223-35.

313. Scott JC, Wright DJM, Cutler SJ. Typing African relapsing fever spirochetes. Emerging Infectious Diseases. 2005;11(11):1722-9. PubMed PMID: 41628350.

314. Pollack RJ, Marcus LC. A travel medicine guide to arthropods of medical importance. Infect Dis Clin North Am. 2005;19(1):169-83. doi: 10.1016/j.idc.2004.10.003.

315. Houhamdi L, Parola P, Raoult D, Author A, L 'Unité des Rickettsies CU, Marseille F, et al. Lice and lice-borne diseases in humans ORIGINAL (NON-ENGLISH) TITLE Les poux et les maladies transmises á l'homme. Médecine tropicale : revue du Corps de santé colonial. 2005;65(1):13-23. PubMed Central PMCID: PMC15903070.

316. Barbour AG. Relapsing fever. Tick-Borne Diseases of Humans. 2005:268-91. PubMed PMID: CCC:000231096800016.

317. Zhang P, Chomel BB, Schau MK, Goo JS, Droz S, Kelminson KL, et al. A family of variably expressed outer-membrane proteins (Vomp) mediates adhesion and autoaggregation in Bartonella quintana. Proc Natl Acad Sci U S A. 2004;101(37):13630-5. doi: 10.1073/pnas.0405284101. PubMed PMID: 15347808; PubMed Central PMCID: PMC518805.

318. Parola P, Raoult D. Global climate change and infectious disease. Archives de Pediatrie. 2004;11(8):1018-25. doi: 10.1016/j.arcped.2003.12.020. PubMed PMID: BIOSIS:PREV200500030924.

319. Meri T, Cutler SJ, Meri S, Jokiranta TS. Relapsing fever Borrelia bind complement regulators factor H, FHL-1, and C4b-binding protein. Molecular Immunology. 2004;41(2-3):276. PubMed PMID: BIOSIS:PREV200500085297.

320. Gangaidzo IT. Forgotten diseases: relapsing fever. The Central African journal of medicine. 2004;50(7-8):73-5. PubMed PMID: 40722630.

321. Burgess IF. Human Lice and Their Control. Annual Review of Entomology2004. p. 457-81.

322. Meri T, Cutler SJ, Hellwage J, Zipfel PF, Meri S, Jokiranta TS. Borrelia recurrentis and B. duttonii evade complement alternative pathway by binding factor H and FHL-1. Molecular Immunology. 2003;40(2-4):178. PubMed PMID: BIOSIS:PREV200300457159.

323. Masuda G, Author A, Tokyo Metropolitan Kiyose Children's H, Correspondence A, G. Masuda TMKCsH. Relapsing fever. Nippon rinsho Japanese journal of clinical medicine. 2003;61 Suppl 2:547-50. PubMed Central PMCID: PMC12722278.

324. Jiang J, Temenak JJ, Richards AL. Real-time PCR duplex assay for Rickettsia prowazekii and Borrelia recurrentis. Annals of the New York Academy of Sciences2003. p. 302-10.

325. Watanabe M. [An outbreak of epidemic louse-borne typhus in Tokyo 1914: a study on the prevention of epidemics]. Nihon ishigaku zasshi [Journal of Japanese history of medicine]. 2002;48(4):597-616. Epub 2003/04/12. PubMed PMID: 12680427.

326. Vidal V, Cutler S, Scragg IG, Wright DJM, Kwiatkowski D. Characterisation of silent and active genes for a variable large protein of Borrelia recurrentis. BMC Infectious Diseases. 2002;2 (no pagination)(25). doi: <http://dx.doi.org/10.1186/1471-2334-2-25>. PubMed PMID: 38750892.

327. Meri T, Cutler SJ, Hellwage J, Zipfel PF, Meri S, Jokiranta TS. Borrelia recurrentis evades complement alternative pathway activation by binding factor H and FHL-1. International Immunopharmacology. 2002;2(9):1345. PubMed PMID: BIOSIS:PREV200200609285.

328. Fournier PE, Ndihokubwayo JB, Guidran J, Kelly PJ, Raoult D. Human pathogens in body and head lice. Emerging Infectious Diseases. 2002;8(12):1515-8. PubMed PMID: 36008797.

329. Outram Q. The socio-economic relations of warfare and the military mortality crises of the Thirty Years' War. Med Hist. 2001;45(2):151-84. PubMed PMID: 11373858; PubMed Central PMCID: PMC1044352.

330. Burgdorfer W. Arthropod-borne spirochetoses: A historical perspective. European Journal of Clinical Microbiology and Infectious Diseases. 2001;20(1):1-5. doi: 10.1007/s10096-001-8034-7. PubMed PMID: BIOSIS:PREV200100154025.

331. Shibeshi D. Pattern of skin disease at the Ethio-Swedish Pediatric Hospital Addis Ababa, Ethiopia. Pediatr Dermatol. 2000;17(5):357-9. doi: 10.1046/j.1525-1470.2000.017005357.x.

332. Scragg IG, Kwiatkowski D, Vidal V, Reason A, Paxton T, Panico M, et al. Structural characterization of the inflammatory moiety of a variable major lipoprotein of Borrelia recurrentis. Journal of Biological Chemistry. 2000;275(2):937-41. doi: <http://dx.doi.org/10.1074/jbc.275.2.937>. PubMed PMID: 30051138.

333. Hartmann P, Schulz H, Romer K, Engert A, Salzberger B. Relapsing fever (Borrelia spec.): An important differential diagnosis in patients returning from Africa. Onkologie. 2000;23(Sonderheft 7):186. PubMed PMID: BIOSIS:PREV200100129784.

334. Goddard J. Human lice and disease. Infections in Medicine. 2000;17(10):660-+. PubMed PMID: WOS:000089967200009.

335. Foucault C, Raoult D. Louse-associated bacterial infections. Infectious Diseases in Clinical Practice. 2000;9(7):281-91. PubMed PMID: 30696217.

336. Cutler SJ, Jones SE, Wright DJM, Zhang H. Cultivation of East African relapsing fever Borrelia and review of preceding events. Journal of Spirochetal and Tick-borne Diseases. 2000;7(FALL):52-8. PubMed PMID: 32448690.

337. Casjens S. Borrelia genomes in the year 2000. J Mol Microbiol Biotechnol. 2000;2(4):401-10.

338. Roux V, Raoult D. Body lice as tools for diagnosis and surveillance of reemerging diseases. Journal of Clinical Microbiology. 1999;37(3):596-9. PubMed PMID: 29091270.

339. Raoult D, Roux V. The body louse as a vector of reemerging human diseases. Clinical Infectious Diseases. 1999;29(4):888-911. PubMed PMID: 30256387.

340. Estanislao LB, Pachner AR. Spirochetal infection of the nervous system. Neurologic Clinics. 1999;17(4):783-+. doi: 10.1016/s0733-8619(05)70166-3. PubMed PMID: WOS:000083789500007.

341. Cutler SJ, Akintunde COK, Moss J, Fukunaga M, Kurtenbach K, Talbert A, et al. Successful in vitro cultivation of Borrelia duttonii and its comparison with Borrelia recurrentis. International Journal of Systematic Bacteriology. 1999;49(4):1793-9. PubMed PMID: 29519112.

342. Cobey FC, Goldbarg SH, Levine RA, Charette L, Patton CL, Tiruha D. Borreliosis relapsing fever, diagnosed by quantitative buffy coat (QBC) fluorescent microscopy. American Journal of Tropical Medicine and Hygiene. 1999;61(3 SUPPL.):419-20. PubMed PMID: BIOSIS:PREV199900476405.

343. Vidal V, Scragg IG, Cutler SJ, Rockett KA, Fekade D, Warrell DA, et al. Variable major lipoprotein is a principal TNF-inducing factor of louse- borne relapsing fever. Nature Medicine. 1998;4(12):1416-20. doi: <http://dx.doi.org/10.1038/4007>. PubMed PMID: 28553465.

344. Jones D. The neglected saliva: medically important toxins in the saliva of human lice. Parasitology. 1998;116 Suppl:S73-81. PubMed PMID: 9695112.

345. Drotman DP. Emerging infectious diseases: a brief biographical heritage. Emerg Infect Dis. 1998;4(3):372-3. PubMed PMID: 9716948; PubMed Central PMCID: PMC2640308.

346. Cutler SJ, Moss J, Fukunaga M, Wright DJM, Fekade D, Warrell D. Borrelia recurrentis initial characterisation and comparison with other borreliae. Journal of Medical Microbiology. 1998;47(5):464. PubMed PMID: BIOSIS:PREV199800378893.

347. Coosemans M, Van Gompel A. Arthropods vectors of diseases. What is the risk for travellers of being bitten? Of being contaminated? Bull Soc Pathol Exot. 1998;91(5BIS):467-73. PubMed PMID: CCC:000078528400023.

348. Winker MA. A global theme issue: bibliography of references. Emerg Infect Dis. 1996;2(4):365-72. PubMed PMID: 9011382; PubMed Central PMCID: PMC2639924.

349. Van Der Laan JR, Smit RBJ, Author A, Gemeentelijke Geneeskundige G P, Netherlands GKU, Correspondence A, et al. Back again: The clothes louse (Pediculus humanus var. corporis) ORIGINAL (NON-ENGLISH) TITLE Terug van weggeweest: De kleerluis (Pediculus humanus var. corporis). Nederlands Tijdschrift voor Geneeskunde. 1996;140(38):1912-5. PubMed PMID: 1996283469; PubMed Central PMCID: PMC8927169.

350. Oliver MR, Liles WC, Spach DW. Relapsing fever. Wilderness and Environmental Medicine. 1996;7(1):46-55. PubMed PMID: 26116126.

351. Doury P. [Henry Foley and the discovery in 1908 of the role played by the louse in the transmission of relapsing fever]. Hist Sci Med. 1996;30(3):363-9. PubMed PMID: 11624987.

352. Stanek G. Borreliosis and Travel Medicine. Journal of travel medicine. 1995;2(4):244-51. Epub 1995/12/01. PubMed PMID: 9815401.

353. Rahlenbeck SI, Gebre-Yohannes A. Louse-borne relapsing fever and its treatment. Tropical and Geographical Medicine. 1995;47(2):49-52. PubMed PMID: 25152657.

354. Mara DD, Alabaster GP. AN ENVIRONMENTAL CLASSIFICATION OF HOUSING-RELATED DISEASES IN DEVELOPING-COUNTRIES. Journal of Tropical Medicine and Hygiene. 1995;98(1):41-51. PubMed PMID: WOS:A1995QG82300007.

355. Litvinjenko S. [How the epidemics of typhus and relapsing fever were stopped in Serbia in 1915 year]. Srp Arh Celok Lek. 1995;123(11-12):328-30. PubMed PMID: 16296250.

356. Franc M, Author A, Ecole nationale vétérinaire de Toulouse F, Correspondence A, M. Franc EnvdTF. Lice and methods of control ORIGINAL (NON-ENGLISH) TITLE Poux et méthodes de lutte. Revue scientifique et technique. 1994;(1994) 13(4):1039-51. PubMed Central PMCID: PMC7711304.

357. World MJ. Pestilence, war, and lice. Lancet. 1993;342(8881):1192. PubMed PMID: 7901526.

358. Patterson KD. Typhus and its control in Russia, 1870-1940. Med Hist. 1993;37(4):361-81. PubMed PMID: 8246643; PubMed Central PMCID: PMC1036775.

359. Oldfield EC, III, Rodier GR, Gray GC. The Endemic Infectious Diseases of Somalia. Clinical Infectious Diseases. 1993;16:S132-S57. doi: 10.1093/clinids/16.Supplement_3.S132.

360. Hardy A. Relapsing fever. Kiple KF, editor: Cambridge University Press, The Pitt Building, Trumpington Street, Cambridge CB2 1RP, England 40 W. 20th Street, New York, New York 10011-4211, USA; 1993. 967-70 p.

361. Grubhoffer L, Uhlir J, Volf P. Functional and structural identification of a new lectin activity of Borrelia recurrentis spirochetes. Comparative Biochemistry and Physiology - B Biochemistry and Molecular Biology. 1993;105(3-4):535-40. PubMed PMID: 23200102.

362. Sinnott JT, Oehler RL, Baran DA, Holt DA. Relapsing fever: Ticked off by a lousy illness. Infections in Medicine. 1992;9(5):18-24. PubMed PMID: 22209744.

363. Grossman M. RELAPSING FEVER1991. 600 p.

364. Rodhain F. Relapsing fevers in the work of charles nicolle. Med Mal Infect. 1989;19(12):768-72. doi: 10.1016/S0399-077X(89)80285-9.

365. Johnson RC. RELAPSING FEVER1989. 1596-8 p.

366. Warrell DA. RELAPSING FEVERS1988. 90-2 p.

367. Eyckmans L, Author A, Institut de Medecine Tropicale A, Correspondence A, Institut de Medecine Tropicale A. Relapsing fevers ORIGINAL (NON-ENGLISH) TITLE LES FIEVRES RECURRENTES. Revue Medicale de Liege. 1988;43(15 16):530-3. PubMed PMID: 1988249435; PubMed Central PMCID: PMC3187258.

368. Barbour AG, Hayes SF. Biology of Borrelia species. Microbiol Rev. 1986;50(4):381-400. PubMed PMID: 3540570; PubMed Central PMCID: PMC373079.

369. Kehl KS. Relapsing fever: Role of borrelial antigens. Clin Microbiol Newsl. 1985;7(4):25-7. doi: 10.1016/S0196-4399(85)80050-7.

370. Horton JM, Blaser MJ. The Spectrum of Relapsing Fever in the Rocky Mountains. Arch Intern Med. 1985;145(5):871-5. doi: 10.1001/archinte.1985.00360050127022.

371. Caldwell JP, Kain BF, McDonald RC. A Canadian Medical Team in Ethiopia. Can Fam Physician. 1985;31:2115-7. PubMed PMID: 21274128; PubMed Central PMCID: PMC2327743.

372. Goubau PF. RELAPSING FEVERS - A REVIEW. Annales De La Societe Belge De Medecine Tropicale. 1984;64(4):335-64. PubMed PMID: WOS:A1984ABX2000002.

373. Teklu B, Habte-Michael A, White NJ, Warrell DA, Wright DJM. OPIATE ANTAGONISTS IN THE JARISCH HERXHEIMER REACTION IN BORRELIAL INFECTIONS. Postgraduate Medical Journal Supplement. 1983;59(1):67. PubMed PMID: BIOSIS:PREV198426058963.

374. Southern PMJ. RELAPSING FEVER1982. P953-62 p.

375. Smith DC. Medical science, medical practice, and the emerging concept of typhus in mid-eighteenth-century Britain. Med Hist Suppl. 1981;(1):121-34. PubMed PMID: 11612143; PubMed Central PMCID: PMC2557366.

376. Kelly RT. THE GENUS BORRELIA1981. P578-81 p.

377. Gutman LT. BORRELIA1980. P878-83 p.

378. Chin J. DISEASES TRANSMITTED PRIMARILY BY ARTHROPOD VECTORS BACTERIAL INFECTIONS RELAPSING FEVER1980. P367-402 p.

379. Patterson KD. Health in urban Ghana: The case of Accra 1900-1940 [1]. Soc Sci Med Part B Med Anthropol. 1979;13(4):251-68. doi: 10.1016/0160-7987(79)90023-1.

380. Malison MD. Relapsing fever. Journal of the American Medical Association. 1979;241(26):2819-20. doi: <http://dx.doi.org/10.1001/jama.241.26.2819>. PubMed PMID: 9228034.

381. Butler T, Wallace CK. EVIDENCE FOR A NON-ENDOTOXIN PYROGEN IN BORRELIA-RECURRENTIS INFECTION. Clinical Research. 1977;25(3):A373-A. PubMed PMID: WOS:A1977DB15801037.

382. Rodhain F. BORRELIA AND RELAPSING FEVERS - TODAY EPIDEMIOLOGICAL FEATURES. Bulletin De L Institut Pasteur. 1976;74(2):173-218. PubMed PMID: WOS:A1976BL56300002.

383. Bryceson AD. Clinical pathology of the Jarisch-Herxheimer reaction. Journal of Infectious Diseases. 1976;133(6):696-704. PubMed PMID: 932495.

384. Perine PL, Reynolds DF. Letter: Relapsing-fever epidemic in the Sudan and Ethiopia. Lancet. 1974;2(7892):1324-5. PubMed PMID: 5443513.

385. Judge DM, Samuel I, Perine PL, Vukotic D. Louse borne relapsing fever in man. Archives of Pathology and Laboratory Medicine. 1974;97(3):136-40. PubMed PMID: 5007461.

386. Judge DM, La Croix JT, Perine PL. Experimental louse borne relapsing fever in the grivet monkey, Cercopithecus aethiops. III. Crisis following therapy. American Journal of Tropical Medicine and Hygiene. 1974;23(5):969-73. PubMed PMID: 5133065.

387. Judge DM, La Croix JT, Perine PL. Experimental louse borne relapsing fever in the grivet monkey, Cercopithecus aethiops. II. Pathology. American Journal of Tropical Medicine and Hygiene. 1974;23(5):962-8. PubMed PMID: 5133064.

388. Judge DM, La Croix JT, Perine PL. Experimental louse borne relapsing fever in the grivet monkey, Cercopithecus aethiops. I. Clinical course. American Journal of Tropical Medicine and Hygiene. 1974;23(5):957-61. PubMed PMID: 5133063.

389. Hovind Hougen K. Electron microscopy of Borrelia merionesi and Borrelia recurrentis. Acta Pathologica et Microbiologica Scandinavica - Section B Microbiology and Immunology. 1974;82 B(6):799-809. PubMed PMID: 6007111.

390. Felsenfeld O. Borrelia. Methods in Microbiology1973. p. 75-94.

391. Dodge RW. Human serological response to louse-borne relapsing fever. Infect Immun. 1973;8(6):891-5. PubMed PMID: 4784888.

392. Dodge RW. Culture of Ethiopian strains of Borrelia recurrentis. Journal of Applied Microbiology. 1973;25(6):935-9. PubMed PMID: 4051497.

393. Bruce-Chwatt LJ. Global Problems of Imported Disease. Advances in Parasitology1973. p. 75-114.

394. Rosen G. Tenements and typhus in New York City, 1840-1875. Am J Public Health. 1972;62(4):590-3. PubMed PMID: 4553573; PubMed Central PMCID: PMC1530136.

395. Warrell DA, Perine PL, Bryceson AD, Parry EH, Pope HM. Physiologic changes during the Jarisch-Herxheimer reaction in early syphilis. A comparison with louse-borne relapsing fever. The American journal of medicine. 1971;51(2):176-85. PubMed PMID: 91484010.

396. Favorova LA, Chernyshova TF, Mikhailov AK. [Results of inoculation of volunteers with Borrelia passaged through lice. VII]. Med Parazitol (Mosk). 1971;40(4):443-6. PubMed PMID: 5134381.

397. Ito K. Studies on comparison of staining of Treponema pallidum with that of Borrelia recurrentis as seen from long-term preservation of the specimen. II. Negative staining. Bulletin of Pharmaceutical Research Institute. 1970;84:1-6. PubMed PMID: 90464254.

398. Ito K. Studies on comparison of staining of Treponema pallidum with that of Borrelia recurrentis as seen from long-term preservation of the specimen. I. Positive staining. Bulletin of Pharmaceutical Research Institute. 1969;78:1-5. PubMed PMID: 90446129.

399. Weyer F, Author A, Correspondence A, Weyer F. Studies on the maintenance and preservation of strains of relapsing fever in the laboratory ORIGINAL (NON-ENGLISH) TITLE Zur Frage der Haltung und Konservierung von Rückfallfieber-Stämmen im Laboratorium. Z Tropenmed Parasitol. 1968;19(3):344-51. PubMed Central PMCID: PMC4976229.

400. Schuster NH. English doctors in Russia in the early nineteenth century. Proc R Soc Med. 1968;61(2):185-90. PubMed PMID: 4870536; PubMed Central PMCID: PMC1902240.

401. Grosser PJ. [Contribution to the epidemiology of quarrantine diseases]. Z Arztl Fortbild (Jena). 1968;62(14):791-7. PubMed PMID: 5728375.

402. Dorolle P. Old plagues in the jet age. International aspects of present and future control of communicable disease. Br Med J. 1968;4(5634):789-92. PubMed PMID: 5702293; PubMed Central PMCID: PMC1912964.

403. Logan JS. The yellow fever of Ireland. Ulster Med J. 1966;35(1):49. PubMed PMID: 5332162; PubMed Central PMCID: PMC2384941.

404. Lippelt H. [Development of world epidemiology]. Z Tropenmed Parasitol. 1966;17(3):245-51. PubMed PMID: 4867186.

405. Felsenfeld O. Borreliae, Human Relapsing Fever, and Parasite-Vector-Host Relationships. Bacteriol Rev. 1965;29(1):46-74. PubMed PMID: 14295985; PubMed Central PMCID: PMC441260.

406. Ludvik J. ELECTRON-MICROSCOPIC STUDY OF BORRELIA RECURRENTIS. Folia Microbiologica. 1962;7(3):200-&. PubMed PMID: WOS:A19621694B00022.

407. Reiss-Gutfreund RJ. [Culture of Borellia recurrentis (Ethiopan strains) in fertilized hen egg]. [French]. Annales de l'Institut Pasteur. 1960;98:131-6. PubMed PMID: 81782721.

408. Heisch RB, Sparrow H, Harvey AE, Author A, Correspondence A, Heisch RB. The behavior of Spirochaeta recurrentis Lebert in lice. Bull Soc Pathol Exot Filiales. 1960;53:140-3. PubMed Central PMCID: PMC13713008.

409. Fulton JD, Smith PJC. Carbohydrate metabolism in Spirochaeta recurrentis. 1. The metabolism of spirochaetes in vivo and in vitro*. Biochem J. 1960;76(3):491-9. PubMed PMID: 13702570; PubMed Central PMCID: PMC1204824.

410. Scheff GJ, Kutner FR. Dehydrogenase activity of Borrelia recurrentis. Experientia. 1959;15(9):342. doi: 10.1007/BF02159820.

411. Galliard H, Lapierre J, Rousset JJ, Author A, Correspondence A, Galliard H. Attenuation to the note on the test for the identification of Spirochaeta recurrentis. Bull Soc Pathol Exot Filiales. 1959;52:269-71. PubMed Central PMCID: PMC13826145.

412. Sparrow H. Etude du foyer éthiopien de fièvre récurrente. Bull World Health Organ. 1958;19(4):673-710 4. PubMed PMID: 13596890; PubMed Central PMCID: PMC2537725.

413. Von Knorre G. [Recurrent typhus fever]. Medizinische. 1957;10(2):85-7. PubMed PMID: 13399734; PubMed Central PMCID: PMCSource: CLML. 5731:46363.

414. Babudieri B. RELAPSING FEVER IN JORDAN. Bull World Health Organ. 1957;16(5):911-28. PubMed PMID: 13472437; PubMed Central PMCID: PMC2538252.

415. Quarantinable Diseases. Br Med J. 1957;1(5032):1406-7. PubMed PMID: 20788678; PubMed Central PMCID: PMC1973680.

416. Sparrow H, Author A, Correspondence A, Sparrow H. Maintenance of Borrelia recurrentis (Ethiopian strains) by passages on newborn mice. ORIGINAL (NON-ENGLISH) TITLE Entretien de Borrelia recurrentis (souches éthiopiennes) par passages sur souriceaux nouveau-nés. Bull Soc Pathol Exot Filiales. 1956;49(2):250-4. PubMed Central PMCID: PMC13342842.

417. Sparrow H. [Use of newborn rats for maintenance of Borrelia recurrentis]. Bull Soc Pathol Exot Filiales. 1956;49(4):630-1. PubMed PMID: 13396512; PubMed Central PMCID: PMCSource: CLML. 5731:43134.

418. Sparrow H. [Recall of observations concerning behavior of spirochetes of recurrent fever in the louse]. Bull Soc Pathol Exot Filiales. 1956;49(2):246-50. PubMed PMID: 13342841; PubMed Central PMCID: PMCSource: CLML. 5630:51420.

419. Heisch RB. Zoonoses as a Study in Ecology. Br Med J. 1956;2(4994):669-73. PubMed PMID: 13356043; PubMed Central PMCID: PMC2035211.

420. Sparrow H. [Focus of relapsing fever transmitted by lice in Ethiopia]. C R Hebd Seances Acad Sci. 1955;241(22):1636-8. PubMed PMID: 13284980; PubMed Central PMCID: PMCSource: CLML. 5629:37202.

421. Kuroda T, Amawashi M, Author A, Correspondence A, Kuroda T. Serological and biological activities of the extract prepared by the mechanical disintegration (Matsumoto-Nagao method) of spirochaeta recurrentis. Annals of tuberculosis. 1955;6(1):19-23. PubMed Central PMCID: PMC13283520.

422. Sparrow H, Author A. Louse as a natural vector of experimental recurrent fever ORIGINAL (NON-ENGLISH) TITLE Le pou agent vecteur naturel et expérimental des fièvres récurrentes. C R Hebd Seances Acad Sci. 1954;239(1):131-3. PubMed Central PMCID: PMC13199981.

423. Serstnev E. [Some characteristics of epidemic of louse-borne relapsing fever in Bosnia in 1946-1949]. Hig Cas Hig Mikrobiol Epidemiol Sanit Teh. 1953;5(2):106-16. PubMed PMID: 13128631; PubMed Central PMCID: PMCSource: CLML. 5425:50088:401.

424. MacArthur W. A Medical Survey of the Irish Famine of 1846: A Robert Campbell Memorial Oration. Ulster Med J. 1951;20(1):1-15. PubMed PMID: 14855826; PubMed Central PMCID: PMC2479424.

425. Beet EA. Transmission of T. Duttoni. Br Med J. 1949;1(4602):501-2. PubMed Central PMCID: PMC2049717.

426. Willcox PH. Louse-borne relapsing fever in Persia. Brit Med J. 1948;1(4548):473. PubMed PMID: 81756242.

427. Pack GT, Grant FR. The Influence of Disease on History*. Bull N Y Acad Med. 1948;24(8):523-40. PubMed PMID: 18875550; PubMed Central PMCID: PMC1871307.

428. Heisch RB, Garnham PC. The transmission of Spirochaeta duttoni Novy & Knapp by Pediculus humanus corporis de Geer. Parasitology. 1948;38(4):247-52. PubMed PMID: 18907587; PubMed Central PMCID: PMCSource: CLML. 4814:731d.

429. Davis WA. The Louse. An account of the lice which infest man, their medical importance and control. Am J Public Health Nations Health. 1948;38(4):578-9. PubMed Central PMCID: PMC1624434.

430. Coghill NF, Gambles RM. Discussion of methods for differentiating tick- from louse-borne relapsing fever spirochaetes. Ann Trop Med Parasitol. 1948;42(1):113-7. PubMed PMID: 18915552; PubMed Central PMCID: PMCSource: CLML. 4814:1070f1.

431. Herms WB. VECTOR POTENTIALITIES WITH RESPECT TO THE SPREAD OF INSECT-BORNE DISEASES OF MAN IN CALIFORNIA. Calif Med. 1947;67(2):95-9. PubMed PMID: 18731281; PubMed Central PMCID: PMC1643004.

432. Baltazard, Author A. Not Available ORIGINAL (NON-ENGLISH) TITLE Identification des spirochétes récurrents; individualité de l'espèce Spirochaeta recurrents. Bull Soc Pathol Exot Filiales. 1947;40(3 4):77-82. PubMed Central PMCID: PMC20270417.

433. Williamson J. Therapeutic action of different penicillins on spirochaeta recurrentis inifecitions in mice. Brit Med J. 1946;1(4456):828-9. doi: 10.1136/bmj.1.4456.828.

434. Preston TW. The Investigation of Prolonged Pyrexia. Postgrad Med J. 1946;22(254):405-8. PubMed PMID: 20287294; PubMed Central PMCID: PMC2478465.

435. Cullinan ER. MEDICAL DISORDERS IN EAST AFRICA. Transactions of the Royal Society of Tropical Medicine and Hygiene. 1946;39(5):353-68. doi: 10.1016/0035-9203(46)90014-4. PubMed PMID: WOS:A1946XT83400001.

436. Chalke HD. Typhus in the Central Mediterranean Force—I. Br Med J. 1946;1(4460):977-80. PubMed PMID: 20786779; PubMed Central PMCID: PMC2059269.

437. Wolman B, Wolman M. STUDIES OF THE BIOLOGICAL PROPERTIES OF SPIROCHAETA-RECURRENTIS IN THE ETHIOPIAN HIGH PLATEAU. Annals of Tropical Medicine and Parasitology. 1945;39(2):82-93. PubMed PMID: WOS:A1945XV68100003.

438. Chen YP, Zia SH, Anderson HH. IMMUNITY REACTIONS IN EXPERIMENTAL RELAPSING FEVER. American Journal of Tropical Medicine. 1945;25(2):115-6. PubMed PMID: WOS:A1945YB51900005.

439. Stein GJ. THE SEROLOGICAL DIAGNOSIS OF RELAPSING FEVER. Journal of Experimental Medicine. 1944;79(1):115-28. doi: 10.1084/jem.79.1.115. PubMed PMID: WOS:000206716900011.

440. Hurst A. Trench Fever. Br Med J. 1942;2(4262):318-20. PubMed PMID: 20784435; PubMed Central PMCID: PMC2164211.

441. Chen KC. Growth of Louse-Borne Relapsing Fever Spirochetes in Chick Embryo. Proc Soc Exp Biol Med. 1941;46(4):638-9. doi: 10.3181/00379727-46-12089P.

442. Kirk R. The non-transmission of abyssinian louse-borne relapsing fever by the tick ornithodorus savignyi and certain other blood-sucking arthropods. Annals of Tropical Medicine and Parasitology. 1938;32(4):357-65. doi: 10.1080/00034983.1938.11685039.

443. Kirk R. A laboratory study of abyssinian louse-borne relapsing fever. Annals of Tropical Medicine and Parasitology. 1938;32(4):339-56. doi: 10.1080/00034983.1938.11685038.

444. Yacob M. A Note on a Circumscribed Outbreak of a Typhus-Like Fever in Muzaffargarh District, South-Western Punjab. Ind Med Gaz. 1937;72(10):585-6. PubMed Central PMCID: PMC5173905.

445. Reynolds FC. Relapsing Fever: Comments on Its Incidence in Nevada. Cal West Med. 1937;47(3):170-4. PubMed PMID: 18744208; PubMed Central PMCID: PMC1753518.

446. Burns GC. Relapsing Fever in California*. Cal West Med. 1936;44(1):29-33. PubMed PMID: 18743503; PubMed Central PMCID: PMC1760192.

447. Wynns HL, Beck MD. Epidemiological Studies on Relapsing Fever in California *. Am J Public Health Nations Health. 1935;25(3):270-6. PubMed PMID: 18014170; PubMed Central PMCID: PMC1559060.

448. O'Connor FW. Concern of the United States with Tropical Diseases *. Am J Public Health Nations Health. 1935;25(1):1-10. PubMed PMID: 18014129; PubMed Central PMCID: PMC1558918.

449. Briggs LH. Relapsing Fever*. Cal West Med. 1935;42(5):350-4. PubMed PMID: 18743245; PubMed Central PMCID: PMC1752157.

450. Hearle E. VECTORS OF RELAPSING FEVER IN RELATION TO AN OUTBREAK OF THE DISEASE IN BRITISH COLUMBIA. Can Med Assoc J. 1934;30(5):494-7. PubMed PMID: 20319493; PubMed Central PMCID: PMC403345.

451. Coleman GE. Relapsing Fever Problem of California *. Am J Public Health Nations Health. 1934;24(10):1056-61. PubMed PMID: 18014066; PubMed Central PMCID: PMC1558867.

452. Palmer JH, Crawford DJM. RELAPSING FEVER IN NORTH AMERICA, WITH REPORT OF AN OUTBREAK IN BRITISH COLUMBIA. Can Med Assoc J. 1933;28(6):643-7. PubMed PMID: 20319139; PubMed Central PMCID: PMC402883.

453. Megaw J. A Note on Professor Nicolle's Views on the Typhus and Relapsing Fevers. Ind Med Gaz. 1933;68(8):462-4. PubMed Central PMCID: PMC5163858.

454. Legge RT. Relapsing Fever — A New Etiological Observation. Cal West Med. 1933;38(5):380. PubMed PMID: 18742522; PubMed Central PMCID: PMC1658625.

455. Varden AE. Relapsing Fever: Report of Case. Cal West Med. 1932;36(5):344-6. PubMed PMID: 18742134; PubMed Central PMCID: PMC1658200.

456. Meleney HE. RELAPSE PHENOMENA OF SPIRONEMA RECURRENTIS. J Exp Med. 1928;48(1):65-82. PubMed PMID: 19869472; PubMed Central PMCID: PMC2131462.

457. Megaw JWD, Gupta JC. The Geographical Distribution of Some of the Diseases of India. Ind Med Gaz. 1927;62(6):299-313. PubMed Central PMCID: PMC5197588.

458. Nicolle C, Anderson C. Recurrent fever transmitted by both ornithodores and lice. Comptes Rendus Hebdomadaires Des Seances De L Academie Des Sciences. 1926;182:1450-1. PubMed PMID: WOS:000200918700252.

459. Reece RJ. President's Address: Progress and Problems in Epidemiology. Proc R Soc Med. 1923;16(Sect Epidemiol State Med):35-48. PubMed PMID: 19983229; PubMed Central PMCID: PMC2103588.

460. History of the Great War. Diseases of the War, Vol. I. Ind Med Gaz. 1923;58(1):43-5. PubMed Central PMCID: PMC5178710.

461. Willcox WH. Typhus and Relapsing Fever in Mesopotamia and Northern Persia 1. Proc R Soc Med. 1920;13(Med Sect):59-81. PubMed PMID: 19981140; PubMed Central PMCID: PMC2152263.

462. Mackie FP. THE TRANSMISSION OF RELAPSING FEVER. Br Med J. 1920;1(3089):380-1. PubMed Central PMCID: PMC2337742.

463. Hunter W. The Serbian Epidemics of Typhus and Relapsing Fever in 1915: Their Origin, Course, and Preventive Measures employed for their Arrest 1: (An Ætiological and Preventive Study based on Records of British Military Sanitary Mission to Serbia, 1915.). Proc R Soc Med. 1920;13(Sect Epidemiol State Med):29-158. PubMed PMID: 19981291; PubMed Central PMCID: PMC2152681.

464. Byam W, Lloyd L. Trench Fever: Its Epidemiology and Endemiology 1. Proc R Soc Med. 1920;13(Sect Epidemiol State Med):1-27. PubMed PMID: 19981284; PubMed Central PMCID: PMC2152678.

465. Mackie FP. Disease in Mesopotamia. Bristol Med Chir J (1883). 1919;36(137):118-31. PubMed PMID: 28897495; PubMed Central PMCID: PMC5057828.

466. Wiese O. The propagation of recurrent fevers. Deutsche Medizinische Wochenschrift. 1918;44:60-2. PubMed PMID: WOS:000201476000024.

467. Buchanan GS. President's Address: Epidemics of the Eastern Campaigns. Proc R Soc Med. 1918;11(Sect Epidemiol State Med):1-30. PubMed PMID: 19980269; PubMed Central PMCID: PMC2066451.

468. Plotz H. CULTIVATION OF SPIROCHAETA OBERMEIERI. The Journal of experimental medicine. 1917;26(1):37-9. doi: 10.1084/jem.26.1.37. PubMed PMID: MEDLINE:19868132.

469. McCarrison R. INDIA AND MEDICAL PROGRESS. Br Med J. 1917;2(2952):109-12. PubMed Central PMCID: PMC2348827.

470. Castellani A. Tropical Diseases in the Balkans. Proc R Soc Med. 1917;10(Med Sect):31-58. PubMed PMID: 19979744; PubMed Central PMCID: PMC2017590.

471. Shipley AE. INSECTS AND WAR: LICE. Br Med J. 1914;2(2803):497-9. PubMed PMID: 20767301; PubMed Central PMCID: PMC2299779.

472. Nicolle C, Blanc G. Are recurring fever spirochaetes virulent in successive phases of their evolution in the louse? Demonstration of their virulence at an invisible stage. Comptes Rendus Hebdomadaires Des Seances De L Academie Des Sciences. 1914;158:1815-7. PubMed PMID: WOS:000200946700647.

473. Steen R, Townsend RS. Relapsing Fever in Bulandshahr District. Ind Med Gaz. 1913;48(9):338-41. PubMed Central PMCID: PMC5177298.

474. Crawfurd R. Contributions from the History of Medicine to the Problem of the Transmission of Typhus. Proc R Soc Med. 1913;6(Sect Hist Med):6-17. PubMed PMID: 19977255; PubMed Central PMCID: PMC2006242.

475. Bhandarkar PR. Relapsing Fever and the Louse. Ind Med Gaz. 1909;44(11):433. PubMed Central PMCID: PMC5132623.

476. Drancourt M, Raoult D, inventors; Inodiag; Inodiag Sa, assignee. In vitro serological diagnosis of infection by spirochete bacteria e.g. Borrelia, comprises contacting serum sample with detection substances and bacterial antigens, and detecting and quantifying serological reactions of immunoglobulins patent WO2009004056-A1; FR2918459-A1; EP2167968-A1; US2010159488-A1.

477. ; Jirous J, assignee. Borreliosis serological diagnosis|using antigen from blood of mice infected by Borrelia recurrentis patent CS8806933-A.

478. Seilmaier M, Guggemos W, Alberer M, Wendtner CM, Spinner CD. Infections among refugees. [German]. Notfall und Rettungsmedizin. 2017;20(3):216-27. doi: <http://dx.doi.org/10.1007/s10049-016-0252-8>. PubMed PMID: 613789999.

479. Marosevic D, Margos G, Wallich R, Wieser A, Sing A, Fingerle V. First insights in the variability of Borrelia recurrentis genomes. PLoS neglected tropical diseases. 2017;11(9):e0005865. Epub 2017/09/14. doi: 10.1371/journal.pntd.0005865. PubMed PMID: 28902847.

480. Margos G, Marosevic D, Cutler S, Derdakova M, Diuk-Wasser M, Emler S, et al. There is inadequate evidence to support the division of the genus Borrelia. International Journal of Systematic and Evolutionary Microbiology. 2017;67(4):1081-4. doi: <http://dx.doi.org/10.1099/ijsem.0.001717>. PubMed PMID: 616071996.

481. Marcinkiewicz AL, Kraiczy P, Lin YP. There is a method to the madness: Strategies to study host complement evasion by lyme disease and relapsing fever spirochetes. Frontiers in Microbiology. 2017;8 (MAR) (no pagination)(328). doi: <http://dx.doi.org/10.3389/fmicb.2017.00328>. PubMed PMID: 615077146.

482. Kortas AZ, Polenz J, von Hayek J, Rüdiger S, Rottbauer W, Storr U, et al. Screening for infectious diseases among asylum seekers newly arrived in Germany in 2015: a systematic single-centre analysis. Public Health. 2017;153:1-8. doi: 10.1016/j.puhe.2017.07.011.

483. Eonomopoulou A, Pavli A, Stasinopoulou P, Giannopoulos LA, Tsiodras S. Migrant screening: Lessons learned from the migrant holding level at the Greek–Turkish borders. J Infect Public Health. 2017;10(2):177-84. doi: 10.1016/j.jiph.2016.04.012.

484. Eiset AH, Wejse C. Review of infectious diseases in refugees and asylum seekers-current status and going forward. Public Health Reviews. 2017;38. doi: 10.1186/s40985-017-0065-4. PubMed PMID: WOS:000411017900001.

485. Ehounoud CB, Fenollar F, Dahmani M, N'Guessan JD, Raoult D, Mediannikov O. Bacterial arthropod-borne diseases in West Africa. Acta Tropica. 2017;171:124-37. doi: <http://dx.doi.org/10.1016/j.actatropica.2017.03.029>. PubMed PMID: 615216203.

486. Cutuli SL, De Pascale G, Ciervo A, Antonelli M. Relapsing fever in young refugees from East Africa response. Critical Care. 2017;21:NIL_1-NIL_2. PubMed PMID: CCC:000406994600002.

487. Cutler SJ, Rudenko N, Golovchenko M, Cramaro WJ, Kirpach J, Savic S, et al. Diagnosing Borreliosis. Vector-Borne and Zoonotic Diseases. 2017;17(1):2-11. doi: 10.1089/vbz.2016.1962.

488. Castelli F, Sulis G. Migration and infectious diseases. Clinical Microbiology and Infection. 2017;23(5):283-9. doi: 10.1016/j.cmi.2017.03.012.

489. Butler T. The Jarisch-Herxheimer Reaction After Antibiotic Treatment of Spirochetal Infections: A Review of Recent Cases and Our Understanding of Pathogenesis. American Journal of Tropical Medicine and Hygiene. 2017;96(1):46-52. PubMed PMID: CCC:000397822900010.

490. Antinori S, Colombo V, Corbellino M. Relapsing fever in young refugees from East Africa. Critical Care. 2017;21 (1) (no pagination)(205). doi: <http://dx.doi.org/10.1186/s13054-017-1777-z>. PubMed PMID: 617618566.

491. Wieser A, Löscher T, Schunk M, Seilmaier M, Balzer L, Margos G, et al. Relapsing fever: An almost forgotten disease in focus again ORIGINAL (NON-ENGLISH) TITLE Rückfallfieber: Eine fast vergessene Erkrankung ist wieder aktuell. Deutsche Medizinische Wochenschrift. 2016;141(14):1009-13. doi: 10.1055/s-0041-110629 FULL TEXT LINK <http://dx.doi.org/10.1055/s-0041-110629>. PubMed PMID: 20160538031; PubMed Central PMCID: PMC27404930.

492. Torreggiani S, Filocamo G, Esposito S. Recurrent Fever in Children. Int J Mol Sci. 2016;17(4). doi: 10.3390/ijms17040448. PubMed PMID: 27023528; PubMed Central PMCID: PMC4848904.

493. Stich A. Frequent infectious diseases in migrants. Internist. 2016;57(5):409-15. doi: 10.1007/s00108-016-0057-3.

494. Sangaré AK, Doumbo OK, Raoult D. Management and Treatment of Human Lice. BioMed Research International. 2016;2016:(12p). doi: 10.1155/2016/8962685. PubMed PMID: 117043031.

495. Ravensbergen SJ, Lokate M, Cornish D, Kloeze E, Ott A, Friedrich AW, et al. High Prevalence of Infectious Diseases and Drug-Resistant Microorganisms in Asylum Seekers Admitted to Hospital; No Carbapenemase Producing Enterobacteriaceae until September 2015. PloS one. 2016;11(5):e0154791. doi: <http://dx.doi.org/10.1371/journal.pone.0154791>. PubMed PMID: 617358866.

496. Papan C, Hübner J, Von Both U. Infectious diseases in refugees and their minors arriving in Germany - What the GP needs to know. MMW-Fortschr Med. 2016;158(4):58-63.

497. Mafra C, Montandon CE. Borreliosis. Arthropod Borne Diseases: Springer International Publishing; 2016. p. 193-204.

498. Leibler JH, Zakhour CM, Gadhoke P, Gaeta JM. Zoonotic and Vector-Borne Infections among Urban Homeless and Marginalized People in the United States and Europe, 1990-2014. Vector-Borne and Zoonotic Diseases. 2016;16(7):435-44. doi: <http://dx.doi.org/10.1089/vbz.2015.1863>. PubMed PMID: 611039087.

499. Jaton L, Kritikos A, Bodenmann P, Greub G, Merz L. [European migrant crisis and reemergence of infections in Switzerland]. Rev Med Suisse. 2016;12(514):749-53. PubMed PMID: 27263151.

500. Fingerle V, Ackermann N, Belting A, Hoch M, Liebl B, Margos G, et al. Louse-borne relapsing fever revisited. Hyg Med. 2016;41(11):D174-D8.

501. Faccini-Martinez AA, Perez-Diaz CE, Botero-Garcia CA, Benitez-Baracaldo FC, Rodriguez-Lopez AE, Rodriguez-Morales AJ. Role of the blood smear in febrile returning travelers: Beyond malaria. Travel Medicine and Infectious Disease. 2016;14(5):515-6. doi: <http://dx.doi.org/10.1016/j.tmaid.2016.03.004>. PubMed PMID: 609610738.

502. Cutler SJ. Refugee crisis and re-emergence of forgotten infections in Europe. Clinical Microbiology and Infection. 2016;22(1):8-9. doi: <http://dx.doi.org/10.1016/j.cmi.2015.10.018>. PubMed PMID: 607170798.

503. Bourée P, Ensaf A. La fièvre récurrente mondiale revient en Europe. Option Bio. 2016;27(549-550):27-8. doi: 10.1016/S0992-5945(16)30235-5.

504. Antinori S, Mediannikov O, Corbellino M, Raoult D. Louse-borne relapsing fever among East African refugees in Europe. Travel Medicine and Infectious Disease. 2016;14(2):110-4. doi: <http://dx.doi.org/10.1016/j.tmaid.2016.01.004>. PubMed PMID: 608330800.

505. Alberer M, Wendeborn M, Löscher T, Seilmaier M. Spectrum of diseases occurring in refugees and asylum seekers: Data from three different medical institutions in the Munich area from 2014 and 2015. Deutsche Medizinische Wochenschrift. 2016;141(1):e8-e15. doi: 10.1055/s-0041-106907.

506. Iwamatsu T, Mitsuno H, Sakurai T, Kanzaki R. Behavioral responses of body lice, Pediculus humanus corporis, to human body-odor components. Chemical Senses. 2015;40 (8):599. doi: <http://dx.doi.org/10.1093/chemse/bjv044>. PubMed PMID: 617302412.

507. Cutler SJ. Relapsing fever borreliosis. Clinical Infectious Disease, Second Edition: Cambridge University Press; 2015. p. 1068-71.

508. Cutler SJ. Relapsing Fever Borreliae: A Global Review. Clinics in Laboratory Medicine. 2015;35(4):847-65. doi: <http://dx.doi.org/10.1016/j.cll.2015.07.001>. PubMed PMID: 605716470.

509. Chikeka I, Dumler JS. Neglected Bacterial Zoonoses. Clin Microbiol Infect. 2015;21(5):404-15. doi: 10.1016/j.cmi.2015.04.022. PubMed PMID: 25964152; PubMed Central PMCID: PMC4466158.

510. Catchpole M, Coulombier D. Refugee crisis demands European union-wide surveillance! Eurosurveillance. 2015;20(45). doi: 10.2807/1560-7917.ES.2015.20.45.30063.

511. Eiset AH, Wejse C. Review of infectious diseases in refugees and asylum seekers—current status and going forward. Public Health Reviews. 2017;38(1). doi: 10.1186/s40985-017-0065-4.

512. Stiefelhagen P. Could you have detected the louse-borne relapsing fever? MMW-Fortschritte der Medizin. 2016;158(20):11-2. doi: 10.1007/s15006-016-8980-8.

513. Hytönen J, Khawaja T, Grönroos JO, Jalava A, Meri S, Oksi J. Relapsing fever. Duodecim; laaketieteellinen aikakauskirja. 2016;132(21):1952-6.

514. Ulutasdemir N, Eroglu F, Tannverdi M, Dagli EI, Koltas IS. The epidemic typhus and trench fever are risk for public health due to increased migration in southeast of Turkey. Acta Tropica. 2018;178:115-8. PubMed PMID: CCC:000423644300018.

515. Palmer C, Landguth E, Stone E, Johnson T. The dynamics of vector-borne relapsing diseases. Mathematical Biosciences. 2018;297:32-42. doi: <http://dx.doi.org/10.1016/j.mbs.2018.01.001>.

516. Munoz-Leal S, Faccini-Martinez AA, Costa FB, Marcili A, Mesquita ETKC, Marques EP, Jr., et al. Isolation and molecular characterization of a relapsing fever Borrelia recovered from Ornithodoros rudis in Brazil. Ticks and Tick-Borne Diseases. 2018;9(4):864-71. doi: 10.1016/j.ttbdis.2018.03.008. PubMed PMID: WOS:000433129600018.

517. Louni M, Amanzougaghene N, Mana N, Fenollar F, Raoult D, Bitam I, et al. Detection of bacterial pathogens in clade E head lice collected from Niger's refugees in Algeria. Parasites & Vectors. 2018;11. doi: 10.1186/s13071-018-2930-5. PubMed PMID: WOS:000435376200002.

518. Isenring E, Fehr J, Gültekin N, Schlagenhauf P. Infectious disease profiles of Syrian and Eritrean migrants presenting in Europe: A systematic review. Travel Medicine and Infectious Disease. 2018. doi: 10.1016/j.tmaid.2018.04.014.

519. Gravinatti ML, Faccini-Martínez ÁA, Ruys SR, Timenetsky J, Biondo AW. Preliminary report of body lice infesting homeless people in Brazil. Revista do Instituto de Medicina Tropical de Sao Paulo. 2018;60. doi: 10.1590/s1678-9946201860009.

520. Alsheri A, Gourley SA. A mathematical model for the transmission of louse-borne relapsing fever. European Journal of Applied Mathematics. 2018;29(3):417-49. PubMed PMID: CCC:000430502200003.

521. Schöffel N, Braun M, Volante G, Bendels MHK, Groneberg DA. Relapsing fever borreliosis: A review of the literature. Zentralbl Arbeitsmed Arbeitssch Ergonomie. 2018;68(4):214-7. doi: 10.1007/s40664-017-0220-2.

522. Powers J, Badri T. Pediculosis Corporis. StatPearls. Treasure Island (FL): StatPearls Publishing

StatPearls Publishing LLC.; 2018.

523. Halperin JJ, García-Moncó JC. The human borreliosis: Lyme neuroborreliosis and relapsing fever. CNS Infections: A Clinical Approach: Second Edition: Springer International Publishing; 2018. p. 233-69.

524. Craigie D. Notice of a febrile disorder which has prevailed at Edinburgh during the summer of 1843. Edinburgh Med Surg J. 1843;60:410–8.

525. Henderson W. On some of the characters which distinguish the fever at present epidemic from typhus fever. Edinburgh Med Surg J 1844;61:201-25.

526. Obermeier O. Vorkommen feinster eine eigenbewegung zeigender faden im blute von rekurrenskranken. Zentralbl Med Wiss. 1873;11:145-7.

527. Carter HV. Spirillum fever : synonyms : famine or relapsing fever as seen in western India: Churchill, London; 1882.

528. Armand-Delille P, Gassin, Lemaire H. Les principaux caractères de la fièvre récurrente observée à l'armée d'Orient. Bulletin et Memoires de la Societe Medicale des Hopitaux de Paris. 1917;33(21-22):778-80 pp.

529. Muhlens P. Arsal treatment, especially in cases of relapsing fever. Dtsch Med Wochenschr. 1917;43(27-52):1167-8. PubMed PMID: WOS:000201475900123.

530. Cragg FW. The epidemiology of relapsing fever in India. Transactions of the Royal Society of Tropical Medicine and Hygiene. 1922;15(8):236-56. doi: <https://doi.org/10.1016/S0035-9203(22)90835-1>.

531. Kulescha GS, Titowa NA. The pathological anatomy and aetiology of complications of relapsing fever. Virchows Archiv für Pathologische Anatomie und Physiologie und für Klinische Medizin. 1923;241:319-551.

532. Nicolle C, Anderson C. Comparative study of some recurrent pathogenic viruses in humans. C R Hebd Seances Acad Sci. 1927;184:1225-6. PubMed PMID: WOS:000200919200167.

533. Kritschewski IL, Sinjuschina MN. Nature of Immunity in Relapsing Fever. XIII. Changes in the Humoral and Phagocytic Protective Mechanism of the Body in Relapsing Fever. Krankheitsforschung. 1931;9(2):139-66 pp.

534. Cunningham J, Theodore JH, Fraser AGL. Further Observations on Indian Relapsing Fever. Part I. Types of Splrochaetes found In Experimental Infections. Indian Journal of Medical Research. 1934;22(1):105-55 pp.

535. Nagelsbach E. A Physician's Experiences in the Highlands of Abyssinia. Archiv fur Schiffs- und Tropenhygiene. 1934;38(3 & 4):100-12; 47-55 pp.

536. Adler S, Ashbel R. Observations on spirochaeta sogdianum nicolle and anderson, 1928, in laboratory animals. Annals of Tropical Medicine and Parasitology. 1937;31(1):89-104. doi: 10.1080/00034983.1937.11684969.

537. Kirk R. The Epidemiology of Relapsing Fever in the Anglo-Egyptian Sudan. Annals of Tropical Medicine and Parasitology. 1939;33(2):125-40 pp.

538. Ishii N, Shimizu S, Tsuda K. The Effect of Sulfapyridine on Experimental Spiroehaetosis Recurrentis. Jikken Igaku Zasshi = Japanese Journal of Experimental Medicine. 1941;19(1-3):5-9 pp.

539. Scott HH. A History of Tropical Medicine. Baltimore: The Williams and Wilkins Co.; 1942.

540. Greig EDW. An Epidemic of Relapsing Fever in Edinburgh In 1843. Edinburgh Medical Journal. 1943;50(11):681-6.

541. Chen YP, Zia SH, Anderson HH. Immunity Reactions in Experimental Relapsing Fever1. The American Journal of Tropical Medicine and Hygiene. 1945;s1-25(2):115-6. doi: doi:<https://doi.org/10.4269/ajtmh.1945.s1-25.115>.

542. Martinez Baez M, Villasana A. Sobre la histopathología de la fiebre recurrente experimental. Revista del Instituto de Salubridad y Enfermedades Tropicales. 1945;6:185-94. Epub 1945/09/01. PubMed PMID: 21018607.

543. Grenoilleau G. L'epidemie de fievre recurrente en Algerie en 1944. Bulletin de l'Academie de medecine. 1946;130(6-8):144. PubMed PMID: MEDLINE:21023922.

544. Ballif L, Constantinesco N, Chelaresco M. Spirochétolysines et réactions de spirochétolyse dans la fièvre récurrente humaine. La Presse medicale. 1947;55(52):586. Epub 1947/09/06. PubMed PMID: 18897788.

545. Balteanu I, Russ M, Voiculescu M. Lytic Properties on Spirochaetes of Convalescent Serum from Cases of Relapsing Fever. Arch Roumaines Path Exper et Microbiol. 1948;15(1/2):310-12.

546. M. B, B. S, C. M, M. B. Donnés expérimentales nouvelles sur la fièvre récurrente épidémique humaine. Bull Acad natMed. 1949;133:284.

547. Heisch RB. Studies in East African Relapsing Fever. East African Medical Journal. 1950;27(1):1-58.

548. Hippocrates, Chadwick J, Mann WN. The Medical Works of Hippocrates. A new translation ... by John Chadwick ... and W.N. Mann. [With a portrait.]. Blackwell Scientific Publications: Oxford; 1950.

549. Guggenheim K, Buechlerczaczkes E, Halevy S. THE EFFECT OF PROTEIN DEFICIENCY ON THE RESISTANCE OF RATS TO INFECTION WITH SPIROCHETES OF RELAPSING FEVER. J Infect Dis. 1951;88(2):105-8. doi: 10.1093/infdis/88.2.105. PubMed PMID: WOS:A1951UM98400001.

550. Schuhardt VT. Treatment of relapsing fever with antibiotics. Annals of the New York Academy of Sciences. 1952;55(6):1209-21. Epub 1952/12/30. PubMed PMID: 13139204.

551. Martini E. ZUR ALTEREN GESCHICHTE DER RECURRENS IM EUROPAISCHEN RAUM. Ergebnisse Der Hygiene Bakteriologie Immunitatsforschung Und Experimentellen Therapie. 1955;29:213-47. PubMed PMID: WOS:A1955WS86300004.

552. Mooser H. DIE RUCKFALLFIEBER. Ergebnisse Der Mikrobiologie Immunitatsforschung Und Experimentellen Therapie. 1958;31:184-228. PubMed PMID: WOS:A1958WN23800004.

553. Sparrow H. [Study of the Ethiopian source of recurrent fever.]. Bull World Health Organ. 1958;19(4):673-710. PubMed PMID: 13596890.

554. Balthazard M. Relapsing fevers. In: National Academy of Sciences WDC, editor. Tropical Health; a Report on a Study of Needs and Resources1962. p. 497.

555. Fendall NRE, Grounds JG. The Incidence and Epidemiology of Disease in Kenya. Part I. Some Diseases of Social Significance. Journal of Tropical Medicine and Hygiene. 1965;68(4):77-84.

556. Fendall NRE, Grounds JG. The Incidence and Epidemiology of Disease in Kenya. Part II. Some Important Communicable Diseases. Journal of Tropical Medicine and Hygiene. 1965;68(5):113-20.

557. Fendall NRE, Grounds JG. The Incidence and Epidemiology of Disease in Kenya. Part III. Insect-Borne Diseases. Journal of Tropical Medicine and Hygiene. 1965;68(6):134-41.

558. Levaditi JC, Balouet G, Juminer B, Corcos A. [Experimental Borrelia infection in newborn rats. Histological study]. Bulletin de la Societe de pathologie exotique et de ses filiales. 1966;59(3):310-6. Epub 1966/05/01. PubMed PMID: 6014078.

559. Southern PM, Sanford JP. RELAPSING FEVER: A Clinical and Microbiological Review. Medicine. 1969;48(2):129-50. PubMed PMID: 00005792-196903000-00002.

560. Cahill KM. Studies in Somalia. Trans R Soc Trop Med Hyg. 1971;65(1):28-42. Epub 1971/01/01. PubMed PMID: 5092427.

561. Felsenfeld O. Borrelia; Strains, Vectors, Human and Animal Borreliosis. St. Louis: Warren H. Green; 1971.

562. Felsenfeld O. The problem of relapsing fever in the Americas. IMS, Industrial medicine and surgery. 1973;42(3):7-10. Epub 1973/03/01. PubMed PMID: 4511068.

563. Burgdorfer W. THE EPIDEMIOLOGY OF THE RELAPSING FEVERS A2 - Johnson, Russell C. Biology of Parasitic Spirochaetes: Academic Press; 1976. p. 191-200.

564. Burgdorfer W. THE DIAGNOSIS OF THE RELAPSING FEVERS A2 - Johnson, Russell C. Biology of Parasitic Spirochaetes: Academic Press; 1976. p. 225-34.

565. Sanford JP. RELAPSING FEVER–TREATMENT AND CONTROL A2 - Johnson, Russell C. Biology of Parasitic Spirochaetes: Academic Press; 1976. p. 389-94.

566. Sholdt LL, Holloway ML, Fronk WD. The epidemiology of human pediculosis in Ethiopia. Jacksonville, Florida: Navy Disease Vector Ecology and Control Center, Naval Air Station.; 1979. xii + 150 pp. p.

567. Butler TC. Relapsing fever: new lessons about antibiotic action. Ann Intern Med. 1985;102(3):397-9. Epub 1985/03/01. PubMed PMID: 3970476.

568. Tesfayohannes T. Prevalence of body lice in elementary school students in three Ethiopian towns at different altitudes. Ethiopian medical journal. 1989;27(4):201-7. Epub 1989/10/01. PubMed PMID: 2598908.

569. Barbour AG. Antigenic variation of a relapsing fever Borrelia species. Annual Review of Microbiology. 1990;44:155-71. doi: 10.1146/annurev.mi.44.100190.001103.

570. Doury P. [Henry Foley and the discovery in 1908 of the role played by the louse in the transmission of relapsing fever]. Histoire des sciences medicales. 1996;30(3):363-9. Epub 1996/01/01. PubMed PMID: 11624987.

571. Ras NM, Lascola B, Postic D, Cutler SJ, Rodhain F, Baranton G, et al. Phylogenesis of relapsing fever Borrelia spp. International journal of systematic bacteriology. 1996;46(4):859-65. Epub 1996/10/01. doi: 10.1099/00207713-46-4-859. PubMed PMID: 8863409.

572. Cadavid D, Barbour AG. Neuroborreliosis during relapsing fever: review of the clinical manifestations, pathology, and treatment of infections in humans and experimental animals. Clinical infectious diseases : an official publication of the Infectious Diseases Society of America. 1998;26(1):151-64. Epub 1998/02/10. PubMed PMID: 9455525.

573. Barrau K, Brouqui P, Jean P, Lafay V, Tissot-Dupont H, D R. Poux de corps, patients sans domicie fixe : les risques infectieux actuels. Bull Epidemiol Hebd. 2000;17:73-4.

574. Raoult D, Foucault C, Brouqui P. Infections in the homeless. The Lancet Infectious diseases. 2001;1(2):77-84. Epub 2002/03/02. doi: 10.1016/s1473-3099(01)00062-7. PubMed PMID: 11871479.

575. Alugupalli KR, Gerstein RM, Chen J, Szomolanyi-Tsuda E, Woodland RT, Leong JM. The Resolution of Relapsing Fever Borreliosis Requires IgM and Is Concurrent with Expansion of B1b Lymphocytes. The Journal of Immunology. 2003;170(7):3819-27. doi: 10.4049/jimmunol.170.7.3819.

576. Bailey AM, Prociv P, Petersen HP. Head lice and body lice: Shared traits invalidate assumptions about evolutionary and medical distinctions2003. 48-62 p.

577. Jonas B, Jean IT, Ulf G, Johan B, Durland F, Alan GB. Typing of <em>Borrelia</em> Relapsing Fever Group Strains. Emerging Infectious Disease journal. 2004;10(9):1661. doi: 10.3201/eid1009.040236.

578. Pound MW, May DB. Proposed mechanisms and preventative options of Jarisch-Herxheimer reactions. Journal of clinical pharmacy and therapeutics. 2005;30(3):291-5. Epub 2005/05/18. doi: 10.1111/j.1365-2710.2005.00631.x. PubMed PMID: 15896248.

579. Barbour AG, Dai Q, Restrepo BI, Stoenner HG, Frank SA. Pathogen escape from host immunity by a genome program for antigenic variation. Proceedings of the National Academy of Sciences of the United States of America. 2006;103(48):18290-5. Epub 2006/11/15. doi: 10.1073/pnas.0605302103. PubMed PMID: 17101971; PubMed Central PMCID: PMCPMC1635980.

580. Raoult D, Dutour O, Houhamdi L, Jankauskas R, Fournier PE, Ardagna Y, et al. Evidence for louse-transmitted diseases in soldiers of Napoleon's Grand Army in Vilnius. The Journal of infectious diseases. 2006;193(1):112-20. Epub 2005/12/03. doi: 10.1086/498534. PubMed PMID: 16323139.

581. Rebaudet S, Parola P. Epidemiology of relapsing fever borreliosis in Europe. FEMS Immunology & Medical Microbiology. 2006;48(1):11-5. doi: 10.1111/j.1574-695X.2006.00104.x.

582. Pettersson J, Schrumpf ME, Raffel SJ, Porcella SF, Guyard C, Lawrence K, et al. Purine salvage pathways among Borrelia species. Infection and immunity. 2007;75(8):3877-84. Epub 2007/05/16. doi: 10.1128/iai.00199-07. PubMed PMID: 17502392; PubMed Central PMCID: PMCPMC1952022.

583. Thein M, Bunikis I, Denker K, Larsson C, Cutler S, Drancourt M, et al. Oms38 is the first identified pore-forming protein in the outer membrane of relapsing fever spirochetes. Journal of bacteriology. 2008;190(21):7035-42. Epub 2008/09/02. doi: 10.1128/jb.00818-08. PubMed PMID: 18757545; PubMed Central PMCID: PMCPMC2580676.

584. Elbir H, Raoult D, Drancourt M. Relapsing Fever Borreliae in Africa. The American Journal of Tropical Medicine and Hygiene. 2013;89(2):288-92. doi: 10.4269/ajtmh.12-0691. PubMed PMID: PMC3741250.

585. Mulat YM, Abera B, Mulu W, Beyene B. Knowledge, Attitude and Practices of High Risk Populations on Louse- Borne Relapsing Fever in Bahir Dar City, North-West Ethiopia. 2014;2:15-22.

586. Sergent E., H. F. Fièvre récurrente du Sud-Oranais et Pediculus vestimenti. Bull Soc Pathol Exot. 1908;1:174.

587. Nicolle C, Blaizot L, Conseil E. Étiologie de la fièvre récurrente. Son mode de transmission par le pou. Comptes Rendus Hebdomadaires des Seances de l'Academie des Sciences. 1912;154:1636.

588. Nicolle C, Blaizot L, Conseil E. Conditions de transmission de la fièvre récurrente par le pou. Comptes Rendus Hebdomadaires des Seances de l'Academie des Sciences. 1912;155:481.

589. Prado Ed. Datos para el estudio de tifus recurrente en el Perú. Cronica Med Lima. 1919;36:408.

590. J.M. C. Primera descriptión del tifus recurrente en el Perú, particularmente observado en el Departemento de Huancavelica. Cronica Med Lima. 1919;36:127-32.

591. Toyoda H. Über die Serumfestigkeit der Recurrensspirochäten und die Heilung der durch sie verursachten Krankheit. Archives of Experimental Medicine. 1919;4:40.

592. K. RS. Zur Kenntnis seltener Hauteruptionen bei typhösen Erkrankungen. Dermatol Wochenschr. 1929;89:981.

593. E. H. Relapsing Fever: Some recent advances. Trop Dis Bull. 1935;32:309.

594. R. G. Relapsing Fever In: D. W, M. R, editors. Infectious Diseases of Man and Animals: Academic Press New York and London; 1968.

595. Di Meco E, Di Napoli A, Amato LM, Fortino A, Costanzo G, Rossi A, et al. Infectious and dermatological diseases among arriving migrants on the Italian coasts. European Journal of Public Health. 2018;28(5):910-6. doi: 10.1093/eurpub/cky126. PubMed PMID: WOS:000452911200025.

596. Guellil M, Kersten O, Namouchi A, Bauer EL, Derrick M, Jensen AO, et al. Genomic blueprint of a relapsing fever pathogen in 15th century Scandinavia. Proceedings of the National Academy of Sciences of the United States of America. 2018;115(41):10422-7. doi: 10.1073/pnas.1807266115.

597. Naddaf SR. Lice, Humans, and Microbes. Iranian Biomedical Journal. 2018;22(5):292-3. PubMed PMID: BIOSIS:PREV201801007126.

598. Barker SC, Barker D. Killing clothes lice by holding infested clothes away from hosts for 10 days to control louseborne relapsing fever, Bahir Dah, Ethiopia. Emerging Infectious Diseases. 2019;25(2):304-10. doi: 10.3201/eid2502.181226.

599. Boumbanda Koyo CS, Amanzougaghene N, Davoust B, Tshilolo L, Lekana-Douki JB, Raoult D, et al. Genetic diversity of human head lice and molecular detection of associated bacterial pathogens in Democratic Republic of Congo. Parasites & vectors. 2019;12(1):290. doi: 10.1186/s13071-019-3540-6. PubMed PMID: MEDLINE:31174587.

600. De Liberato C, Magliano A, Romiti F, Menegon M, Mancini F, Ciervo A, et al. Report of the human body louse (Pediculus humanus) from clothes sold in a market in central Italy. Parasites and Vectors. 2019;12(1). doi: 10.1186/s13071-019-3458-z.

601. El Hamzaoui B, Laroche M, Bechah Y, Berenger JM, Parola P. Testing the Competence of Cimex lectularius Bed Bugs for the Transmission of Borrelia recurrentis, the Agent of Relapsing Fever. The American journal of tropical medicine and hygiene. 2019. doi: <http://dx.doi.org/10.4269/ajtmh.18-0804>.

602. Powers J, Badri T. Pediculosis Corporis. StatPearls. Treasure Island (FL): StatPearls Publishing

StatPearls Publishing LLC.; 2019.

603. Warrell DA. Louse-borne relapsing fever (Borrelia recurrentis infection). Epidemiology and infection. 2019;147:e106. doi: 10.1017/S0950268819000116.

604. Ackermann N, Marosevic D, Hoermansdorfer S, Eberle U, Rieder G, Treis B, et al. Screening for infectious diseases among newly arrived asylum seekers, Bavaria, Germany, 2015. Eurosurveillance. 2018;23(10):2-12. doi: 10.2807/1560-7917.Es.2018.23.10.17-00176. PubMed PMID: WOS:000427079700001.

605. Atkey OPH. Relapsing Fever in the (Anglo-Egyptian) Sudan in 1930. Bulletin de l'Office International d'Hygiene Publique. 1931;23(11):2000-6 pp.

606. Jarisch A. Therapeutische Versuche bei Syphilis. Wien Med Wochenschr 1895;45:721-4.

607. Herxheimer K. Ueber eine bei Syphilitischen vorkommende Quecksilberreaktion. Dtsch Med Wochenschr. 1902;28:895-7. doi: 10.1055/s-0028-1139096.

608. Wright DJM. The relapsing fever spirochaete: Recognition of fulfillment of Koch's postulates. Journal of Medical Microbiology. 1998;47(5):463. PubMed PMID: BIOSIS:PREV199800378888.

609. Cutler SJ, Bottieau E. Migrant health—a cause for concern? Clinical Microbiology and Infection. 2017;23(5):281-2. doi: 10.1016/j.cmi.2017.04.014.

610. Houhamdi L, Raoult D. Excretion of living Borrelia recurrentis in feces of infected human body lice. Journal of Infectious Diseases. 2005;191(11):1898-906. doi: <http://dx.doi.org/10.1086/429920>. PubMed PMID: 40705398.

611. Seilmaier M, Guggemos W, Wieser A, Fingerle V, Balzer L, Fenzl T, et al. [Louse-borne-relapsing-fever in refugees from the Horn of Africa; a case series of 25 patients]. Deutsche Medizinische Wochenschrift. 2016;141(14):e133-42. doi: <https://dx.doi.org/10.1055/s-0042-108180>. PubMed PMID: 27404939.

612. Kesztyus B, Cornish D. Relapsing fever in refugee. International Journal of Medical Microbiology. 2016;306 (8 Supplement 1):134. doi: <http://dx.doi.org/10.1016/j.ijmm.2016.11.008>. PubMed PMID: 614104699.

613. Warrell DA, Parry EHO. CARDIORESPIRATORY COMPLICATIONS OF HIGH FEVER . A PHYSIOLOGICAL STUDY OF FEBRILE REACTION FOLLOWING TETRACYCLINE IN PATIENTS WITH LOUSE-BORNE RELAPSING FEVER. Quarterly Journal of Medicine. 1969;38(152):522-&. PubMed PMID: WOS:A1969E458700019.

614. Gaud M, Morgan MT. Epidemiological Study on Relapsing Fever in North Africa (1943-1945). Bull World Health Organ. 1948;1(1):69-92. PubMed PMID: 20603922; PubMed Central PMCID: PMC2556137.

615. H T. Studien ueber die recurrensspirochaeten in Mandschurien. Kitasato Arch Exp Med. 1919;3:42.

616. Coxon R, Fekade D, Knox K, Hussein K, Melka A, Daniel A, et al. The effect of antibody against TNF alpha on cytokine response in Jarisch-Herxheimer reactions of louse-borne relapsing fever. QJM : monthly journal of the association of physicians [Internet]. 1997; 90(3):[213-21 pp.]. Available from: <http://onlinelibrary.wiley.com/o/cochrane/clcentral/articles/331/CN-00138331/frame.html>.

617. Salih SY, Mustafa D. Louse-borne relapsing fever: II. Combined penicillin and tetracycline therapy in 160 Sudanese patients. Trans R Soc Trop Med Hyg. 1977;71(1):49-51. PubMed PMID: 871033.

618. Parry EH, Warrell DA, Perine PL, Vukotich D, Bryceson AD. Some effects of louse-borne relapsing fever on the function of the heart. The American journal of medicine. 1970;49(4):472-9. PubMed PMID: 90487948.

619. McCowen WT. Bilious Typhus Relapsing Fever. Ind Med Gaz. 1906;41(10):387-96. Epub 1906/10/01. PubMed PMID: 29006131; PubMed Central PMCID: PMCPMC5181027.

620. Walker EA. Spirillum Fever in India. The Indian Medical Gazette. 1905;40(8):320-. PubMed PMID: PMC5162931.

621. Cutler S, Fekade D, Cann K, Emilianus R, Warrell D, Wright D. Isolation and cultivation of Borrelia recurrentis from louse-borne relapsing fever patients in Ethiopia. Abstracts of the General Meeting of the American Society for Microbiology. 1994;94(0):125. PubMed PMID: BIOSIS:PREV199497343772.

622. D'Ignazio C. [Not Available]. Monogr Boll. 1945;5(4):125-38. PubMed PMID: 21028583; PubMed Central PMCID: PMCSource: CLML. 4610:1248l.

623. Perez-Tanoira R, Jado I, Martin-Martin I, Prieto-Perez L, Anda P, Gonzalez Martin-Nino R, et al. Arthropod-borne pathogens as cause of non-malarial fever in rural Ethiopia. Tropical Medicine and International Health. 2017;22(Supplement 1):41. doi: <http://dx.doi.org/10.1111/%28ISSN%291365-3156>.

624. P.B.M. M. Epidemic relapsing fever among the Chinese at Oroville. Pac Med Srug J. 1875;17:370-5.

625. Nicolle C, Blaizot L, Conseil E. Etiology of recurring fever. The means of transmission by lice. C R Hebd Seances Acad Sci. 1912;154:1636-8. PubMed PMID: WOS:000200951300632.

626. Parrot L. Du délire et des réactions psychomotrices dans la fièvre récurrente algérienne. Bulletin de la Société de Pathologie Exotique. 1917;10(8):692-4 pp.

627. Cleland JB. A Death from Relapsing Fever in Australia. Medical Journal. 1938;1(19):820-1 pp.

628. Stuart G. Relapsing Fever in North Africa and Europe, 1943-1945. Epidemio-logical Information Bull (UNRRA Health Division). 1945;1(11):453-64.

629. El-Ramly AH. Three Papers on Louse Borne Relapsing Fever. I. Treatment of Louse Borne Relapsing Fever. Journal of the Egyptian Public Health Association. 1946;21(8):125-49.

630. El-Ramly AH. Three Papers on Louse Borne Relapsing Fever. II. Splenic Infarctions. Journal of the Egyptian Public Health Association. 1946;21(8):150-65.

631. El-Ramly AH. Three Papers on Louse Borne Relapsing Fever. III. Report on 139 Fatal Cases of Louse Borne Relapsing Fever. Journal of the Egyptian Public Health Association. 1946;21(8):166-82.

632. Kamal AM, Anwar M, Abdel Messih G, Kolta Z. Louse-borne Relapsing Fever in Egypt. A Review of 9,977 Cases. Journal of the Egyptian Public Health Association. 1947;22(1):1-22.

633. Ling CC. A Preliminary Study of the Treatment of Chinese Louse-borne Relapsing Fever with Penicillin. Chinese Medical Journal. 1947;65(7/8):225-30.

634. Serstnev E. Some Aspects of Louse-Borne Relapsing Fever Epidemic In Bosnia 1946-1949. Higijena. 1953;5(2):106-16.

635. May JM. Studies in Disease Ecology: New York 3 : Hafner Publishing Co., Inc., 31, East 10th Street, N.Y., U.S.A.; 1961. xx + 613 pp. p.

636. Bucco G. L'organizzazione sanitaria in Africa Orientale: Istituto poligrafico dello stato; 1965.

637. Shrimpton EAG. A Survey of the Incidence of Relapsing Fever in China. Chinese Medical Journal. 1936;(Supp. No. 1):312-44 pp.

638. Stuart G. The typhus-relapsing fever association. Wkly Bull Epidemiol Inf Receiv. 1946;2(16):665-74. PubMed PMID: 20996920; PubMed Central PMCID: PMCSource: CLML. 4611:693h.

639. Peake E.C. (1912) Medical experience in south Hunan. China M.J. 26; 5

640. Zau F.D. (1935) Meningism of Helminthic origin Chinese Med. J. 49; 689

641. Skinner J. E. (1919) Relapsing fever in Fukien. Chinese Med. J. 33; 210

642. Chang C. S. (1936) the chemotherapy of relapsing fever. Nat med j china 22; 177

643. Toyoda H. (1931) Relapsing fever Nat med j china 17; 233

644. BERDUGO, J. MORENO. (1948) Sobre la fiebre recurrente mediteranea y su tratamiento con penicillina. Rev. Clin. Espafiola. 28 ; 262-267

645. Meleney H.E. (1928) Relapse phenomena of Spironema recurrentis J.exper.med. 48; 65

646. T’ung T. and Chung H.L. (1938) The Kolmer’s Wassermann, Kahn and Kline tests in relapsing fever. Chinese Med j supplement 2; 315-324

647. Inversen (1910) Ueber die Wirkung den neben Arsenopräparates Erlichs bei recurrens munch mediz woch n5

648. Paucot (1911) bull soc med chirurg de l’indochine n8

649. Conseil E. (1913) chimiotherapie de la fievre recurrente archives de l’institut pastuer de tunis fasc II

650. GERARD S (1945) Note clinique sur 857 cas de fievre recurrente mondiale Maroc med 24; 138

651. Bousfield L. (1911) Notes on human spirochaetosis. 4th rep. Wellcome trop. Res. Lab., A, 62.

652. Fan J. H. (1945) Communicable diseases in China during recent years. Epid Inform Bull UNRRA (Health Division) 1; 495

653. Chiao S. M. (1945) the epidemiological investigation and control of relapsing fever outbreak at Tung Chi, Kweichow 1940. Acta brev. Sinensia no. 9; 4

654. Forteza Bover J A, Garrigues Orellana y F Marco Orts (1949) fiebre recurrente cosmopolita en Valencia. Rev San e Hig publ 23; 3

655. Gimeno de Sande A. Brote epidemico de fievre recurrente cosmopolita en Motril. Rev San e Hi publ 23; 694

656. Fraser J. (1877) Chinese Imp customs med rep no14 p66

657. Graham A. (1910) Chinese Imp customs med rep no68/80 p97

658. Neil J. B. (1890) China med jour. 4; 245

659. Taylor H.B. (1913) China med jour. 27; 318

660. Logan O.T. (1913) China med jour. 27; 321

661. Scott A. V. (1931) Nat med jour china. 17; 782

662. LeGac J. (1931) Ann Med Pharm Colon. 29; 148

663. Atkey O.H.P. (1929) Bulletin de l'Office International d'Hygiene Publique 21; 1932

664. Franchini G. (1930) Arch Ital Sci Med Colon 11; 449

665. Schilling V. (1921) Beiheft Arch schiff Trop Hyg 25; 5

666. Wright H D and Harold C H H (1919) Brit Med J ii; 526

667. Roy S C (1920) Indian Med Gaz 56; 320

668. Gill C A (1922) Indian J Med Res 9; 747

669. Casaux J (1912) Rev Med Hyg Trop 9; 97

670. Wilcox C (1944) Trop Med Bull 41; 791

671. Miller P.B.M. (1875) Epidemic relapsing fever among the Chinese at Oroville; Pac Med Srug J 17;370

672. Connor R C (1917) Proc Med Assoc Isthmus Canal Zone 10; 67

673. Leon L A and Leon B C (1947) Rev Kuba 3; 1 45

674. Cantacuzène J (1929) Bull Soc Pathol Exot 13; 269

675. Lipinski W (1949) Polski Tygod Lek 5; 1465

676. Chiao S M (1945) Acta Brevia Sinensica 1; 4

677. Halawani A (1946) J Egypt Publ Hlth Asso 21; 183

678. Heisch R B (1947) East Afr Med J 24; 3

679. Saglam T (1947) Türk Tip Cemiyeti Mekmuasi 13; 26

680. Zaharia N I (1948) Rev Stiint Med Bucurest 57; 209

681. Petrilla A and Rudnay O (1949) Nepegeszsegügy 39; 439

682. Anonymous (1969) Rel Epidem Hebdomad Org Mond Sante 44; 425

683. Sibilia D (1937) Policlinico 44;722
