# Supplementary material for: Louse-borne relapsing fever—A systematic review and analysis of the literature: Part 2—Mortality, Jarisch–Herxheimer reaction, impact on pregnancy
Source: PLoS Negl Trop Dis. 2021 Mar 11;15(3):e0008656. doi: 10.1371/journal.pntd.0008656 (PMC7951929; doi:10.1371/journal.pntd.0008656)
Supplement: S6 Text — Included and excluded references in pregnancies section. (DOCX) [file pntd.0008656.s007.docx]

**S6 Text. Included and excluded references in pregnancies section**

**Included**

[1-14]

**Excluded**

Not sufficient data on the total number of pregnant women

[15, 16]

No data on pregnancies

[17-184]

1. Benhamou E. Aspects actuels de la fièvre recurrente épidémique en Afrique du Nord. Bulletin de l'Academie de medecine. 1945;129(25-29):530-2. Epub 1945/01/01. PubMed PMID: 21008569.

2. Borgnolo G, Hailu B, Ciancarelli A, Almaviva M, Woldemariam T. Louse-borne relapsing fever. A clinical and an epidemiological study of 389 patients in Asella Hospital, Ethiopia. Trop Geogr Med. 1993;45(2):66-9. PubMed PMID: 8511813.

3. Brown V, Larouze B, Desve G, Rousset JJ, Thibon M, Fourrier A, et al. Clinical presentation of louse-borne relapsing fever among Ethiopian refugees in northern Somalia. Ann Trop Med Parasitol. 1988;82(5):499-502. PubMed PMID: 3257078.

4. Bryceson ADM, Parry EHO, Perine PL, Warrell DA, Vukotich D, Leithead CS. A clinical and laboratory study of 62 cases in ethiopia and a reconsideration of the literature1. QJM. 1970;39(1):129-70. doi: 10.1093/oxfordjournals.qjmed.a067198.

5. Chung H-L, Chang FC. Relapsing Fever. Clinical and Statistical Study of 337 Cases. Chinese Medical Journal. 1939;55(1):6-33 pp.

6. De Jong J, Wilkinson RJ, Schaeffers P, Sondorp HE, Davidson RN. Louse-borne relapsing fever in southern Sudan. Transactions of the Royal Society of Tropical Medicine and Hygiene. 1995;89(6):621. PubMed PMID: 26016342.

7. Gebrehiwot T, Fiseha A. Tetracycline versus penicillin in the treatment of louse-borne relapsing fever. Ethiopian medical journal [Internet]. 1992; 30(3):[175-81 pp.]. Available from: <http://onlinelibrary.wiley.com/o/cochrane/clcentral/articles/444/CN-00087444/frame.html>.

8. Ingraham HS, Lapenta RG, Author A, Correspondence A, Ingraham HS. Penicillin in the treatment of louse-borne relapsing fever. United States naval medical bulletin. 1946;46(11):1719-23. PubMed Central PMCID: PMC21002713.

9. McCuLloch WE. Relapsing Fever in Northern Nigeria-A Study of 300 Cases. Journal of Tropical Medicine and Hygiene. 1925;28(18):332-41 pp.

10. Robertson RC. Relapsing Fever in Shanghai (First Report). Chinese Medical Journal. 1932;46(9):853-85 pp.

11. Sergent E, Foley H. L'épidémiologie de la fiévre récurrente dans l'afrique du nord. Transactions of the Royal Society of Tropical Medicine and Hygiene. 1922;16(3):170-87. doi: <https://doi.org/10.1016/S0035-9203(22)90496-1>.

12. El Ramley AH. Relapsing fever. Relapsing Fever Journal of the Egyptian Public Health Association. 1946;21:1-48.

13. Garnham PC, Davies CW. An epidemic of louse-borne relapsing fever in Kenya. Transactions of the Royal Society of Tropical Medicine and Hygiene. 1947;41(1):141-70. PubMed PMID: 608181014.

14. Jukes AM. Preliminary note on some cases of Spirillar Fever in the Darjeeling district. Indian Med Gaz. 1912;47:476-7.

15. Salih SY, Mustafa D, Abdel Wahab SM, Ahmed MA, Omer A. Louse-borne relapsing fever: I. A clinical and laboratory study of 363 cases in the Sudan. Trans R Soc Trop Med Hyg. 1977;71(1):43-8. PubMed PMID: 871032.

16. Bruns A. On Relapsing Fever in Abyssinia. Archiv fur Schiffs- und Tropenhygiene. 1937;41(3):343-8 pp.

17. Nitzan O, Blum A, Marva E, Katz A, Tzadok BS, Nachum-Biala Y, et al. Case report: Infectious diseases in pilgrims visiting the holy land. American Journal of Tropical Medicine and Hygiene. 2017;97(2):611-4. doi: <http://dx.doi.org/10.4269/ajtmh.17-0097>. PubMed PMID: 617714405.

18. Jochum J, Tannich E, Tappe D, Schmiedel S. A Somali refugee with fever and abnormal blood smear. Internist. 2017;58(3):287-92. doi: 10.1007/s00108-016-0154-3.

19. Hytonen J, Khawaja T, Gronroos JO, Jalava A, Meri S, Oksi J. Louse-borne relapsing fever in Finland in two asylum seekers from Somalia. Apmis. 2017;125(1):59-62. doi: <http://dx.doi.org/10.1111/apm.12635>. PubMed PMID: 613575266.

20. Grecchi C, Zanotti P, Pontarelli A, Chiari E, Tomasoni LR, Gulletta M, et al. Louse-borne relapsing fever in a refugee from Mali. Infection. 2017;45(3):373-6. doi: <http://dx.doi.org/10.1007/s15010-017-0987-2>. PubMed PMID: 614317686.

21. Cutuli SL, De Pascale G, Spanu T, Dell'Anna AM, Bocci MG, Pallavicini F, et al. Lice, rodents, and many hopes: A rare disease in a young refugee. Critical Care. 2017;21 (1) (no pagination)(81). doi: <http://dx.doi.org/10.1186/s13054-017-1666-5>. PubMed PMID: 615072527.

22. Bloch-Infanger C, Battig V, Kremo J, Widmer AF, Egli A, Bingisser R, et al. Increasing prevalence of infectious diseases in asylum seekers at a tertiary care hospital in Switzerland. PLoS One. 2017;12(6):e0179537. doi: 10.1371/journal.pone.0179537. PubMed PMID: BIOSIS:PREV201700716182.

23. Zammarchi L, Antonelli A, Bartolini L, Pecile P, Trotta M, Rogasi PG, et al. Louse-Borne Relapsing Fever with Meningeal Involvement in an Immigrant from Somalia to Italy, October 2015. Vector-Borne and Zoonotic Diseases. 2016;16(5):352-5. doi: <http://dx.doi.org/10.1089/vbz.2015.1928>. PubMed PMID: 610464500.

24. Von Both U, Alberer M. Borrelia recurrentis infection. New England Journal of Medicine. 2016;375(5):e5. doi: <http://dx.doi.org/10.1056/NEJMicm1513366>. PubMed PMID: 611576321.

25. Seilmaier M, Guggemos W, Wieser A, Fingerle V, Balzer L, Fenzl T, et al. 25 Cases of Louse-borne Relapsing Fever in Refugees from East Africa. Deutsche Medizinische Wochenschrift. 2016;141(14):E133-E42. PubMed PMID: CCC:000381007800002.

26. Osthoff M, Schibli A, Fadini D, Lardelli P, Goldenberger D. Louse-borne relapsing fever - report of four cases in Switzerland, June-December 2015. BMC Infectious Diseases. 2016;16 (1) (no pagination)(210). doi: <http://dx.doi.org/10.1186/s12879-016-1541-z>. PubMed PMID: 610362799.

27. Lucchini A, Lipani F, Costa C, Scarvaglieri M, Balbiano R, Carosella S, et al. Louseborne relapsing fever among East African refugees, Italy, 2015. Emerging Infectious Diseases. 2016;22(2):298-301. doi: <http://dx.doi.org/10.3201/eid2202.151768>. PubMed PMID: 607873433.

28. Keller C, Zumblick M, Streubel K, Eickmann M, Muller D, Kerwat M, et al. Hemorrhagic diathesis in borrelia recurrentis infection imported to Germany. Emerging Infectious Diseases. 2016;22(5):917-9. doi: <http://dx.doi.org/10.32032/eid2205.151557>. PubMed PMID: 609995922.

29. Hertting O, Bennet R, Luthander J, Eriksson M. Infection-related hospitalisations in refugee children-a one year study from Northern Stockholm. Cogent Medicine Conference: 8th Excellence in Pediatrics Conference. 2016;3(1). doi: <http://dx.doi.org/10.1080/2331205X.2016.1265203>. PubMed PMID: 614265435.

30. Darcis G, Hayette MP, Bontems S, Sauvage AS, Meuris C, Van Esbroeck M, et al. Louse-borne relapsing fever in a refugee from Somalia arriving in Belgium. Journal of Travel Medicine. 2016;23(3). doi: <http://dx.doi.org/10.1093/jtm/taw009>. PubMed PMID: 614260542.

31. Costescu Strachinaru DI, Cambier J, Kandet-Yattara H, Konopnicki D. Relapsing fever in asylum seekers from Somalia arriving in Belgium in August 2015. Acta Clinica Belgica: International Journal of Clinical and Laboratory Medicine. 2016;71(5):353-5. doi: <http://dx.doi.org/10.1080/17843286.2016.1157942>. PubMed PMID: 612314718.

32. Colomba C, Scarlata F, Di Carlo P, Giammanco A, Fasciana T, Trizzino M, et al. Fourth case of louse-borne relapsing fever in Young Migrant, Sicily, Italy, December 2015 Mini Review Article. Public Health. 2016;139:(5p). doi: 10.1016/j.puhe.2016.05.019. PubMed PMID: 118802975.

33. Ciervo A, Mancini F, Di Bernardo F, Giammanco A, Vitale G, Dones P, et al. Louseborne relapsing fever in young migrants, sicily, Italy, july-september 2015. Emerging Infectious Diseases. 2016;22(1):152-3. doi: <http://dx.doi.org/10.3201/eid2201.151580>. PubMed PMID: 607341375.

34. Benz R, Majer S. Unexpected cause of high fever in the blood film. Blood. 2016;127(18):2264. doi: <http://dx.doi.org/10.1182/blood-2016-01-691980>. PubMed PMID: 610329290.

35. Antinori S, Mediannikov O, Corbellino M, Grande R, Parravicini C, Bestetti G, et al. Louse-Borne Relapsing Fever (Borrelia recurrentis) in a Somali Refugee Arriving in Italy: A Re-emerging Infection in Europe? PLoS Neglected Tropical Diseases. 2016;10 (5) (no pagination)(e0004522). doi: <http://dx.doi.org/10.1371/journal.pntd.0004522>. PubMed PMID: 610558073.

36. Wilting KR, Stienstra Y, Sinha B, Braks M, Cornish D, Grundmann H. Louse-borne relapsing fever (Borrelia recurrentis) in asylum seekers from Eritrea, The Netherlands, July 2015. Eurosurveillance. 2015;20(30):3. PubMed PMID: 605514444.

37. Hoch M, Wieser A, Loscher T, Margos G, Purner F, Zuhl J, et al. Louse-borne relapsing fever (Borrelia recurrentis) diagnosed in 15 refugees from northeast Africa: Epidemiology and preventive control measures, Bavaria, Germany, July to October 2015. Eurosurveillance. 2015;20(42). doi: <http://dx.doi.org/10.2807/1560-7917.ES.2015.20.42.30046>. PubMed PMID: 606659340.

38. Goldenberger D, Claas GJ, Bloch-Infanger C, Breidthardt T, Suter B, Martinez M, et al. Louse-borne relapsing fever (Borrelia Recurrentis) in an Eritrean refugee arriving in Switzerland, August 2015. Eurosurveillance. 2015;20(32). PubMed PMID: 605710959.

39. Gitta SN, Kamadjeu R, Mwesiga A. Proceedings of the 2013 AFENET Scientific Conference - Posters sessions. Pan Afr Med J. 2015;21. doi: 10.11604/pamj.2015.21.209.7259. PubMed Central PMCID: PMC4586171.

40. Yimer M, Mulu W, Ayalew W, Abera B. Louse-borne relapsing fever profile at Felegehiwot referral hospital, Bahir Dar city, Ethiopia: a retrospective study. BMC research notes. 2014;7:250. doi: <http://dx.doi.org/10.1186/1756-0500-7-250>. PubMed PMID: 602155400.

41. Yimer M, Abera B, Mulu W, Bezabih B, Mohammed J. Prevalence and risk factors of louse-borne relapsing fever in high risk populations in Bahir Dar city Northwest, Ethiopia. BMC research notes. 2014;7:615. doi: <http://dx.doi.org/10.1186/1756-0500-7-615>. PubMed PMID: 604824174.

42. Alfaifi AA, Masoodi I, Alzaidi O, Hussain S, Khurshid S, Sirwal IA. Spirocheatal shock syndrome. Indian Journal of Medical Microbiology. 2014;32(2):183-5. doi: <http://dx.doi.org/10.4103/0255-0857.129822>. PubMed PMID: 372815941.

43. Chandra EM, Mandefro Y, Yinges S, Chere BT. Is over-dependence on malaria RDTS disguising morbidity due to other deadly hemoparasites at peripheral health facilities? American Journal of Tropical Medicine and Hygiene. 2013;1):241. PubMed PMID: 71312626.

44. Aarsland SJ, Castellanos-Gonzalez A, Lockamy KP, Mulu-Droppers R, Mulu M, White AC, et al. Treatable Bacterial Infections Are Underrecognized Causes of Fever in Ethiopian Children. Am J Trop Med Hyg. 2012;87(1):128-33. doi: 10.4269/ajtmh.2012.12-0171. PubMed PMID: 22764303; PubMed Central PMCID: PMC3391037.

45. Ramos JM, Malmierca E, Reyes F, Tesfamariam A. Louse-borne relapsing fever in Ethiopian children: Experience of a rural hospital. Tropical Doctor. 2009;39(1):34-6. doi: <http://dx.doi.org/10.1258/td.2008.080157>. PubMed PMID: 354731406.

46. Ramos JM, Malmierca E, Reyes F, Tesfamariam A. Results of a 10-year survey of louse-borne relapsing fever in southern Ethiopia: A decline in endemicity. Annals of Tropical Medicine and Parasitology. 2008;102(5):467-9. doi: <http://dx.doi.org/10.1179/136485908X300887>. PubMed PMID: 352008728.

47. Ramos JM, Reyes F, Tesfamariam A, Malmierca E. Louse-borne relapsing fever and malaria co-infection in Ethiopia. Tropical Doctor. 2007;37(2):121-2. doi: <http://dx.doi.org/10.1258/004947507780609419>. PubMed PMID: 354729852.

48. Melaku Z, Alemayehu M, Oli K, Tizazu G. Pattern of admissions to the Medical Intensive Care Unit of Addis Ababa University Teaching Hospital. Ethiopian Medical Journal. 2006;44(1):33-42.

49. Ramos JM, Malmierca E, Reyes F, Wolde W, Galata A, Tesfamariam A, et al. Characteristics of louse-borne relapsing fever in Ethiopian children and adults. Annals of Tropical Medicine and Parasitology. 2004;98(2):191-6. doi: <http://dx.doi.org/10.1179/000349804225003136>. PubMed PMID: 38388887.

50. Mitiku K, Mengistu G. Relapsing fever in Gondar, Ethiopia. East African Medical Journal. 2002;79(2):85-7.

51. Cobey FC, Goldbarg SH, Levine RA, Patton CL. Short report: Detection of Borrelia (relapsing fever) in rural Ethiopia by means of the quantitative buffy coat technique. American Journal of Tropical Medicine and Hygiene. 2001;65(2):164-5. PubMed PMID: 32744225.

52. Porcella SF, Raffel SJ, Schrumpf ME, Schriefer ME, Dennis DT, Schwan TG. Serodiagnosis of louse-borne relapsing fever with glycerophosphodiester phosphodiesterase (GlpQ) from Borrelia recurrentis. Journal of Clinical Microbiology. 2000;38(10):3561-71. PubMed PMID: 30768416.

53. Cooper PJ, Fekade D, Remick DG, Grint P, Wherry J, Griffin GE. Recombinant human interleukin-10 fails to alter proinflammatory cytokine production or physiologic changes associated with the Jarisch-Herxheimer reaction. Journal of Infectious Diseases. 2000;181(1):203-9. doi: <http://dx.doi.org/10.1086/315183>. PubMed PMID: 30049050.

54. Orloski K, Tharmaphornpilas P, O'Leary D, Ryan M, Schriefer M, Shoo R, et al. Epidemic louse-borne relapsing fever, Rumbek County, Sudan, 1998-1999. American Journal of Tropical Medicine and Hygiene. 1999;61(3 SUPPL.):223-4. PubMed PMID: BIOSIS:PREV199900470785.

55. Cutler SJ, Moss J, Fukunaga M, Wright DJM, Fekade D, Warrell D. Borrelia recurrentis characterization and comparison with relapsing- fever, lyme-associated, and other Borrelia spp. International Journal of Systematic Bacteriology. 1997;47(4):958-68. PubMed PMID: 27441217.

56. Remick DG, Negussie Y, Fekade D, Griffin G. Pentoxifylline fails to prevent the Jarisch-Herxheimer reaction or associated cytokine release. Journal of Infectious Diseases. 1996;174(3):627-30. PubMed PMID: 26299701.

57. Mekasha A, Meharie S. Outbreak of louse-borne relapsing fever in Jimma, south western Ethiopia. East African medical journal. 1996;73(1):54-8. PubMed PMID: 126225733.

58. Fekade D, Knox K, Hussein K, Melka A, Lalloo DG, Coxon RE, et al. Prevention of Jarisch-Herxheimer reactions by treatment with antibodies against tumor necrosis factor α. New England Journal of Medicine. 1996;335(5):311-5. doi: 10.1056/NEJM199608013350503 FULL TEXT LINK <http://dx.doi.org/10.1056/NEJM199608013350503>. PubMed PMID: 1996331136; PubMed Central PMCID: PMC8663853.

59. Seboxa T, Rahlenbeck S. Treatment of louse-borne relapsing fever with low dose penicillin or tetracycline: a clinical trial. Scandinavian journal of infectious diseases [Internet]. 1995; 27(1):[29-31 pp.]. Available from: <http://onlinelibrary.wiley.com/o/cochrane/clcentral/articles/105/CN-00115105/frame.html>.

60. Cuevas LE, Borgnolo G, Hailu B, Smith G, Almaviva M, Hart CA. Tumour necrosis factor, interleukin-6 and C-reactive protein in patients with louse-borne relapsing fever in Ethiopia. Annals of Tropical Medicine and Parasitology. 1995;89(1):49-54. PubMed PMID: 25151119.

61. Knox K, Fekade D, Hussein K, Melka A, Coxon R, Smith D, et al. Ovine polyclonal anti-TNF fab antibody suppresses Jarische-Herxheimer reaction of Louse-Borne relapsing fever. Abstracts of the Interscience Conference on Antimicrobial Agents and Chemotherapy. 1994;34(0):143. PubMed PMID: BIOSIS:PREV199598025505.

62. De Beer PAM. Louse-borne relapsing fever. Giulio Borgnolo et al. Tropical and Geographical Medicine 1993;45(2):66-9. Tropical and Geographical Medicine. 1994;46(3):192. PubMed PMID: 24220492.

63. Sundnes KO, Haimanot AT. Epidemic of louse-borne relapsing fever in Ethiopia.[Erratum appears in Lancet 1994 Jan 22;334(8891):244]. Lancet. 1993;342(8881):1213-5. PubMed PMID: 7901534.

64. Borgnolo G, Denku B, Chiabrera F, Hailu B. Louse-borne relapsing fever in Ethiopian children: A clinical study. Annals of Tropical Paediatrics. 1993;13(2):165-71. PubMed PMID: 23193344.

65. Aknaviva M, Hailu B, Borgnolo G, Chiabrera F, Tolesse G, Gebre B. Louse-borne relapsing fever epidemic in Arssi Region, Ethiopia: A six months survey. Transactions of the Royal Society of Tropical Medicine and Hygiene. 1993;87(2):153. doi: <http://dx.doi.org/10.1016/0035-9203%2893%2990466-4>. PubMed PMID: 23136331.

66. Negussie Y, Remick DG, DeForge LE, Kunkel SL, Eynon A, Griffin GE. Detection of plasma tumor necrosis factor, interleuklns 6, and 8 during the jarisch-herxheimer reaction of relapsing fever. Journal of Experimental Medicine. 1992;175(5):1207-12. doi: 10.1084/jem.175.5.1207.

67. Mekasha A. Louse-borne relapsing fever in children. Journal of Tropical Medicine and Hygiene. 1992;95(3):206-9. PubMed PMID: 22183251.

68. Borgnolo G, Hailu B, Chiabrera F. Louse-borne relapsing fever in Ethiopia. Lancet. 1991;338(8770):827. PubMed PMID: 1681201.

69. Haider MJ, Zafar F, Khan KR. A SURVEY OF BORRELIA-RECURRENTIS IN HUMAN BLOOD IN KARACHI PAKISTAN. Karachi University Journal of Science. 1990;18(1-2):89-94. PubMed PMID: BIOSIS:PREV199293018028.

70. Zein ZA. Louse borne relapsing fever (LBRF): Mortality and frequency of Jarisch-Herxheimer reaction. Journal of the Royal Society of Health. 1987;107(4):146-7. PubMed PMID: 17127609.

71. Isacsohn M, Gondard G, Yaoul E, Gindacu A, Kadosh D, Greenberg Z. LOUSE-BORNE RELAPSING FEVER IN ETHIOPIAN IMMIGRANTS. Israel Journal of Medical Sciences. 1985;21(8):710. PubMed PMID: BIOSIS:PREV198630047889.

72. Warrell DA, Perine PL, Krause DW, Bing DH, MacDougal SJ. Pathophysiology and immunology of the Jarisch-Herxheimer-like reaction in louse-borne relapsing fever: Comparison of tetracycline and slow-release penicillin. Journal of Infectious Diseases. 1983;147(5):898-909. PubMed PMID: 13094242.

73. Teklu B, Habte-Michael A, Warrell DA, White NJ, Wright DJM. MEPTAZINOL DIMINISHES THE JARISCH HERXHEIMER REACTION OF RELAPSING FEVER. Lancet. 1983;1(8329):836-9. PubMed PMID: BIOSIS:PREV198376081118.

74. Perine PL, Teklu B. Antibiotic treatment of louse-borne relapsing fever in Ethiopia: A report of 377 cases. American Journal of Tropical Medicine and Hygiene. 1983;32(5):1096-100. PubMed PMID: 13012101.

75. Butler T, Aikawa M, Habte-Michael A, Wallace C. Phagocytosis of Borrelia recurrentis by blood polymorphonuclear leukocytes is enhanced by antibiotic treatment. Infection and Immunity. 1980;28(3):1009-13. PubMed PMID: 10122535.

76. Ahmed MAM, Wahab SMA, Malik MOA. Louse-borne relapsing fever in the Sudan. A historical review and a clinico-pathological study. Tropical and Geographical Medicine. 1980;32(2):106-11. PubMed PMID: 11250204.

77. Butler T, Hazen P, Wallace CK. Infection with Borrelia recurrentis: Pathogenesis of fever and petechiae. Journal of Infectious Diseases. 1979;140(5):665-75. PubMed PMID: 10214189.

78. Butler T, Jones PK, Wallace CK. Borrelia recurrentis infection: single-dose antibiotic regimens and management of the Jarisch-Herxheimer reaction. Journal of Infectious Diseases. 1978;137(5):573-7. PubMed PMID: 8331814.

79. Ormsbee R, Peacock M, Philip R, Casper E, Plorde J, Gabre-Kidan T, et al. Serologic diagnosis of epidemic typhus fever. American journal of epidemiology. 1977;105(3):261-71. Epub 1977/03/01. PubMed PMID: 403761.

80. Galloway RE, Levin J, Butler T, Naff GB, Goldsmith GH, Saito H, et al. Activation of protein mediators of inflammation and evidence for endotoxemia in Borrelia recurrentis infection. American Journal of Medicine. 1977;63(6):933-8. PubMed PMID: 8251835.

81. Dennis DT, Awoke S, Doberstyn EB, Fresh JW. Bleeding in louse borne relapsing fever in Ethopia. Clinical and laboratory features in 29 patients. East African Medical Journal. 1976;53(4):220-5. PubMed PMID: 7139090.

82. Perine PL, Reynolds DF. RELAPSING-FEVER EPIDEMIC IN THE SUDAN AND ETHIOPIA. Lancet. 1974;304(7892):1324-5. doi: 10.1016/S0140-6736(74)90189-5.

83. Perine PL, Krause DW, Awoke S, McDade JE. Single dose doxycycline treatment of louse borne relapsing fever and epidemic typhus. Lancet. 1974;2(7883):742-4. PubMed PMID: 5111608.

84. Knaack RH, Wright LJ, Leithead CS, Kidan TG, Plorde JJ. Penicillin vs. tetracycline in the treatment of louse borne relapsing fever. A preliminary report. Ethiopian Medical Journal. 1972;10(1):15-22. PubMed PMID: 4035835.

85. Bryceson AD, Cooper KE, Warrell DA, Perine PL, Parry EH. Studies of the mechanism of the Jarisch-Herxheimer reaction in louse-borne relapsing fever: evidence for the presence of circulating Borrelia endotoxin. Clinical science. 1972;43(3):343-54. PubMed PMID: 92522463.

86. Rijkels DF, Author A, Correspondence A, Rijkels DF. Louse-borne relapsing fever in Ethiopia. Tropical and Geographical Medicine. 1971;23(4):335-40.

87. Perine PL, Parry EH, Vukotich D, Warrell DA, Bryceson AD. Bleeding in louse-borne relapsing fever. I. Clinical studies in 37 patients. Transactions of the Royal Society of Tropical Medicine and Hygiene. 1971;65(6):776-81. PubMed PMID: 92372178.

88. Perine PL, Kidan TG, Warrell DA, Bryceson AD, Parry EH. Bleeding in louse-borne relapsing fever. II. Fibrinolysis following treatment. Transactions of the Royal Society of Tropical Medicine and Hygiene. 1971;65(6):782-7. PubMed PMID: 92372179.

89. Warrell DA, Pope HM, Parry EH, Perine PL, Bryceson AD. Cardiorespiratory disturbances associated with infective fever in man: studies of Ethiopian louse-borne relapsing fever. Clinical science. 1970;39(1):123-45. PubMed PMID: 90429807.

90. Abdalla RE. Some studies on relapsing fever in the Sudan. The Journal of tropical medicine and hygiene. 1969;72(5):125-8. PubMed PMID: 89111553.

91. Schofield TP, Talbot JM, Bryceson AD, Parry EH. Leucopenia and fever in the "Jarisch-Herxheimer" reaction of louse-borne relapsing fever. Lancet. 1968;1(7533):58-62. PubMed PMID: 88019294.

92. Ombati DG, Ojiambo HP. Louse-borne relapsing fever. East African medical journal. 1968;45(9):630-1. PubMed PMID: 89018323.

93. Parry EH, Bryceson AD, Leithead CS. Acute hemodynamic changes during treatment of louse-borne relapsing fever. Lancet. 1967;1(7481):81-3. PubMed PMID: 87050972.

94. Cochran S. The Story of Hope Hospital*. Bull N Y Acad Med. 1961;37(1):47-65. PubMed PMID: 13694213; PubMed Central PMCID: PMC1804639.

95. Anderson TR, Zimmerman LE. RELAPSING FEVER IN KOREA - A CLINICOPATHOLOGIC STUDY OF 11 FATAL CASES WITH SPECIAL ATTENTION TO ASSOCIATION WITH SALMONELLA INFECTIONS. American Journal of Pathology. 1955;31(6):1083-109. PubMed PMID: WOS:A1955WD46500007.

96. Hirschboeck MM. THE USE OF CHLORAMPHENICOL IN RELAPSING FEVER. American Journal of Tropical Medicine and Hygiene. 1954;3(4):712-3. PubMed PMID: WOS:A1954UY65900016.

97. Zimmerman LE. Some Experiences with Enteric Diseases in Korea *†: Perforation of Paratyphoid Ulcers and Salmonella Septicemia Complicating Relapsing Fever. Am J Public Health Nations Health. 1953;43(3):279-84. PubMed PMID: 13030878; PubMed Central PMCID: PMC1620059.

98. Tadić RM. An epidemiological investigation of a rural district in Yugoslavia*. Bull World Health Organ. 1952;7(4):431-44. PubMed PMID: 13032787; PubMed Central PMCID: PMC2554137.

99. Harrison IB, Whittington RM. Antibiotics in the treatment of relapsing fever. United States Armed Forces medical journal. 1951;2(12):1859-62. PubMed PMID: MEDLINE:14884341.

100. Legerton CW, Chambers WL. Spontaneous rupture of the spleen in relapsing fever. United States Armed Forces medical journal. 1950;1(1):88-90. PubMed PMID: MEDLINE:15404480.

101. Gaud M, Bey MK, Vaucel M. The Evolution of the Epidemic of Relapsing Fever, 1942-1946. Bull World Health Organ. 1948;1(1):93-101. PubMed PMID: 20603923; PubMed Central PMCID: PMC2556134.

102. Bodman R, Steward I. Louse-borne relapsing fever in Persia. Brit Med J [Internet]. 1948; 4545:[291-3 pp.]. Available from: <http://onlinelibrary.wiley.com/o/cochrane/clcentral/articles/384/CN-00608384/frame.html>.

103. Chafic H, Atallah S, Author A, Correspondence A, Chafic H. Louse born relapsing fever in Haifa. Journal Palestine Arab Medical Association. 1946;2(1):8-13. PubMed Central PMCID: PMC20250218.

104. Wolman M. Louse-borne relapsing fever treated with calcium gold keratinate. Lancet (London, England). 1945;2(6381):775-7. PubMed PMID: 608087033.

105. Greaves FC, Gezon HM, Alston WF, Author A, Correspondence A, Greaves FC. Studies on louse-borne relapsing fever in Tunisia. United States naval medical bulletin. 1945;45:1029-48. PubMed Central PMCID: PMC21004211.

106. Robinson P. Relapsing Fever in Addis Ababa. Br Med J. 1942;2(4259):216-7. PubMed PMID: 20784396; PubMed Central PMCID: PMC2164011.

107. Charters AD. Relapsing fever in Abyssinia. Transactions of the Royal Society of Tropical Medicine and Hygiene. 1942;35(5):271-9. doi: 10.1016/S0035-9203(42)90045-2.

108. Mallannah S. Relapsing Fever in Raichur. Ind Med Gaz. 1923;58(4):168. PubMed Central PMCID: PMC5178439.

109. Sinton JA. Relapsing Fever at Meshed, North-East Persia. Ind Med Gaz. 1921;56(7):241-50. PubMed Central PMCID: PMC5166278.

110. Newcomb C. On an Outbreak of Relapsing Fever in Turkey in 1918. Ind Med Gaz. 1920;55(6):208-17. PubMed Central PMCID: PMC5180621.

111. Fry AS. An Epidemic of Fifty-Four Cases of Relapsing Fever Observed in Birjand, East Persia. Ind Med Gaz. 1920;55(1):2-8. PubMed Central PMCID: PMC5180769.

112. Stott H. On Two Varieties of Relapsing Fever Spirochætal Infection in India. Ind Med Gaz. 1911;46(8):292-8. PubMed Central PMCID: PMC5171664.

113. Mackie FP. THE PART PLAYED BY PEDICULUS CORPORIS IN THE TRANSMISSION OF RELAPSING FEVER. Br Med J. 1907;2(2450):1706-9. PubMed PMID: 20763589; PubMed Central PMCID: PMC2358735.

114. Amanzougaghene N, Akiana J, Mongo Ndombe G, Davoust B, Nsana NS, Parra HJ, et al. Head Lice of Pygmies Reveal the Presence of Relapsing Fever Borreliae in the Republic of Congo. PLoS Neglected Tropical Diseases. 2016;10 (12) (no pagination)(e0005142). doi: <http://dx.doi.org/10.1371/journal.pntd.0005142>. PubMed PMID: 613987217.

115. Boutellis A, Mediannikov O, Bilcha KD, Ali J, Campelo D, Barker SC, et al. Borrelia recurrentis in head lice, Ethiopia. Emerging Infectious Diseases. 2013;19(5):796-8. doi: <http://dx.doi.org/10.3201/eid1905.121480>. PubMed PMID: 368809123.

116. Brouqui P, Stein A, Dupont HT, Gallian P, Badiaga S, Rolain JM, et al. Ectoparasitism and vector-borne diseases in 930 homeless people from Marseilles. Medicine. 2005;84(1):61-8. doi: <http://dx.doi.org/10.1097/01.md.0000152373.07500.6e>. PubMed PMID: 40130185.

117. Raoult D, Birtles RJ, Montoya M, Perez E, Tissot-Dupont H, Roux V, et al. Survey of three bacterial louse-associated diseases among rural Andean communities in Peru: Prevalence of epidemic typhus, trench fever, and relapsing fever. Clinical Infectious Diseases. 1999;29(2):434-6. PubMed PMID: 29380213.

118. Ali M. Relapsing Fever *Paper read at the All-India Sub-Assistant Surgeons' onference at Agra. Ind Med Gaz. 1918;53(5):178-80. PubMed Central PMCID: PMC5201680.

119. Conseil E, Bienassis E. Traitement de la fièvre récurrente par le Néosalvarsan d'Ehrlich. Bull Soc Pathol Exot. 1912;7:476.

120. Hermant. Note sur la fièvre récurrente dans la province de Nghê-An. Bull Soc Med Chir Indochine. 1912;3:427.

121. Mouzels P, Nguyen XM. Note sur 373 cas du fièvre récurrente traités au Lazaret de Hanoi par le 606 au cours de l'année 1912 de 1er janvier au 1er juin. Bulletin de la Société Médico-Chirurgicale de l'Indochine. 1912;3:427.

122. Sterling-Okuniewski S. Der Blutdruck im Verlaufe von Rückfallfieber. Cbl Bact Abt I Orig. 1918;82:456.

123. Jouveau-Dubreuil H. Formule leukocytaire et diminution des éosinophiles dans la fièvre récurrente. Bull Soc Pathol Exot. 1919;12:621.

124. Margolis A. Beobachtungen über Rückfallfieber. Beitr Klin Infektkrk. 1919;7:254.

125. Toyoda H. Studien über die Recurrensspirochäten in Mandschurien. Archives of Experimental Medicine. 1919;3:42.

126. Jouveau-Dubreuil H. Etude clinique sur la fièvre récurrente du Setchouen (Chine occidentale). Bull Soc Pathol Exot. 1920;13:38.

127. Prado Ed. Estudio del tifus recurrente en el Perú. Ann Fac Med Lima. 1920;3:26-45 and 134-52.

128. Sergent E, Foley H. Fièvre récurrente et ictère. Bull Soc Pathol Exot. 1921;14:632.

129. Oettinger J, Halbreich J. Ueber das Vorkommen einer ephemeren Roseola beim Rückfallfieber. Münch Med Woch. 1922;69:788.

130. Levy GS. Typhus recurrens im Kindesalter. Zeitschr Kinderh. 1926;42:627.

131. Chung HL. Presence of spirochetes in Urine and Prostatic Fluid of patients with Relapsing Fever. Proceedings of the Society for Experimental Biology and Medicine. 1938;38:97-8.

132. Böger A. Die latente Rekurrensinfektion und das Rekurrensrheumatoid. Münch Med Woch. 1943;90:549.

133. Robinson P. Typhus Fever in Addis Ababa. Ann Trop Med Parasit. 1943;37:38.

134. Chakrabarty A. Relapsing Fever (Louse borne) in north-east Bengal. J Indian Med Assoc. 1948;18:352-3.

135. Darwish AE. The Effect of Penicillin on the Behaviour of Spirochaetes in Natural and Inoculated Relapsing Fever. J Egypt Med Assoc. 1949;32:605-9.

136. Ravina A, Pêcher Y, Avril J. Typhus récurrente contracté à Paris. Bull et Mém Soc Med Hopt de Paris. 1950;11-12:508-10.

137. Smirnoff PP. Die Anwendung des Salvarsans bei Febris recurrens. Dtsch med Wochenschr. 1912;38(16):748-9. doi: 10.1055/s-0029-1189447.

138. Millous. Les Epidémies Fébriles non classées et la Fièvre Récurrente dans le Thanh-hoa en 1910, 1911, et 1912. . Bulletin de la Societe Medico-Chirurgicale de l'Indochine. 1913;4(1):14-8 pp.

139. Conseil E. Le Galyl et le Ludyl dans le Traitement de la Fièvre Récurrente. Bulletin de la Société de Pathologie Exotique. 1914;7(2):101-5 pp.

140. Foley H, Vialatte C. Traitement de la Fièvre Récurrente Nord-Africaine par le Néosalvarsan et I'Olarsol. . Bulletin de la Société de Pathologie Exotique. 1914;7(7):569-71 pp.

141. Babes V. Hémorragies méningées et autres manifestations hémorragiques dans la fièvre récurrente. OR Sianc Soc Biol (Paris). 1916;79:855.

142. Duchamp JC. Contribution to the Pathology of the Balkans: Serbian Spirochaeto-plasmodial Fever. Bulletin de la Société de Pathologie Exotique. 1917;10(9):827-34 pp.

143. Duchamp JC. La fievre recurrente chez les Serbes. Le progrès médical, Paris. 1917:10-3.

144. Dudgeon L. EXAMINATION OF THE URINE IN CASES OF RELAPSING FEVER OCCURRING IN MACEDONIA. The Lancet. 1917;190(4918):823-5. doi: <https://doi.org/10.1016/S0140-6736(01)56862-2>.

145. Hoesslin HV. Clinical picture of relapsing fever. Munchener Medizinische Wochenschrift. 1917;64(34):1106-9.

146. Kostoff KH. Arsal treatment in cases of relapsing fever. Dtsch Med Wochenschr. 1917;43(27-52):1168-9. doi: 10.1055/s-0028-1144668. PubMed PMID: WOS:000201475900124.

147. Porot A. Délire et réactions psychomotrices dans la fièvre récurrente de l'indigène. Bulletin de la Société de Pathologie Exotique. 1917;10(7):532-6 pp.

148. Portocalis A. Un cas d'insuffisance surrénale aiguë au cours de ia fièvre récurrente ; lésions préexistantes des capsules surrénales. Bulletin et Memoires de la Societe Medicale des Hopitaux de Paris. 1917;33(11-12):545-50 pp.

149. Tausig, Jurinac. A Case of Ruptured Spleen in R. F. Wiener Klinische Wochenschrift. 1917;30(52):1651 p.

150. Dumitresco M. Injections intraveineuses d'arrhénal dans la fièvre récurrente. Presse Medicale. 1918;26(17):155-6 pp.

151. Loewy R. The clinical picture and therapy of relapsing fever. Medizinische Klinik. 1918;14(3):62-3.

152. Portocalis A. The treatment of recurring fever. Comptes Rendus Seances Soc Biol Fil. 1918;81:273-4. PubMed PMID: WOS:000200953700111.

153. Sterling-Okuniewski S. Der Blutdruck im Verlaufe von Rückfallfieber. Dtsch med Wochenschr. 1918;44(10):265-6. doi: 10.1055/s-0028-1134308.

154. Calwell WK. RELAPSING FEVER:: AN ACCOUNT OF A SERIES OF 125 CASES, WITH SPECIAL REFERENCE TO THE PALESTINE TYPE. The Lancet. 1920;196(5068):785-8. doi: <https://doi.org/10.1016/S0140-6736(01)19765-5>.

155. Roy SC. Relapsing Fever Epidemic in Seoni District (Central Provinces), February to May, 1920. Indian Medical Gazette. 1921;56(1):7-9 pp.

156. Kerrest J, Gambier A, Bouron A. Recurrent fever in [French] Sudan. Bulletin de la Société de Pathologie Exotique. 1922;15(5):320-31.

157. Selwyn-Clarke PS, Le Fanu GH, Ingram A. Relapsing Fever in. the Gold Coast. Annals of Tropical Medicine and Parasitology. 1923;17(3):389-426 pp.

158. Cunningham J. Serological Observations on Relapsing Fever in Madras. Transactions of the Royal Society of Tropical Medicine and Hygiene. 1925;19(1 and 2):11-33 pp.

159. Beveridge GEG. The Louse-Borne Type of Relapsing Fever as Prevalent in the Anglo-Egyptian Sudan, 1926-and 1927. Medical Journal of Australia. 1928;1(4):110-2 pp.

160. Belezky WK, Umanskaja RM. Die Recurrensspirochätose des zentralen Nervensystems des Menschen. Z f d g Neur u Psych. 1930;129(1):21-41. doi: 10.1007/BF02865045.

161. Chu F-Ta, Deitrick S, Chung S-F. Relapsing Fever in Children. A Study of Twenty-Six Epidemic Cases. National Medical Journal of China. 1931;17(2):224-32 pp.

162. Russell H. Human and Experimental Relapsing Fever, Accra, Gold Coast, 1929-1930. West African Medical Journal. 1931;4(3):59-66 pp.

163. Cunningham J, Fraser AGL. Further Observations on Indian Relapsing Fever. Part II. The Serology of Relapsing Fever in Human Beings. Indian Journal of Medical Research. 1935;22(4):595-616 pp.

164. Chung H-L. Studies on the Transmission of Relapsing Fever in North China. Preliminary Observations. Chinese Medical Journal. 1936;50(12):1723-34 pp.

165. Chang SL. Relapsing fever in Changsha. A report of 41 cases. Chinese Med J. 1938;54:163-72.

166. Chung H-L. The Cerebrospinal Fluid of Patients suffering from the Chinese Strain of Relapsing Fever. Transactions of the Royal Society of Tropical Medicine and Hygiene. 1938;31(6):625-34 pp. doi: 10.1016/S0035-9203(38)90155-5.

167. Chung H-L, Wei Y-L. Studies on the Transmission of Relapsing Fever in North China II. Observations on the Mechanism of Transmission of Relapsing Fever in Man. The American Journal of Tropical Medicine and Hygiene. 1938;s1-18(6):661-74. doi: doi:<https://doi.org/10.4269/ajtmh.1938.s1-18.661>.

168. Wolman M. Observations on the Value of Treatment in Louse-borne Relapsing Fever. East African Medical Journal. 1944;21(11):336-40.

169. Wolff BP. ASiatic relapsing fever; report of 134 cases treated with mapharsen*. Annals of Internal Medicine. 1946;24(2):203-16. doi: 10.7326/0003-4819-24-2-203.

170. Gaud M, Morgan M. Etude épidémiologique sur la fièvre récurrente en afrique du nord (1943-1945). Bull Wrld Hlth Org. 1947;1:75-98.

171. Shaul JF, Saferstein TH. Penicillin therapy in relapsing fever; report of four cases. United States naval medical bulletin. 1947;47(2):238-43. Epub 1947/03/01. PubMed PMID: 20288517.

172. Corkill NL. Activation of Latent Kala-Azar by Malaria and Relapsing Fever. Annals of Tropical Medicine & Parasitology. 1948;42(2):230-5. doi: 10.1080/00034983.1948.11685367.

173. Nasr LA. STERILE SPLENIC ABSCESS AFTER RELAPSING FEVER. Lancet. 1948;254(APR10):555-8. PubMed PMID: WOS:A1948UB99100004.

174. Senecal J, Ahmad A. [Treatment of recurrent fever with penicillin]. La semaine des hopitaux : organe fonde par l'Association d'enseignement medical des hopitaux de Paris. 1950;26(35):1634-8. Epub 1950/05/10. PubMed PMID: 15418271.

175. Vukotic D. Clinical and electrocardiographic findings of the heart in patients with louse-borne relapsing fever at the " day of crisis " and seven days later. Ethiopian medical journal. 1968;6(5):167-70.

176. Bolton BH, Anderson H, R K. Imported louse-borne relapsing fever -Ohio. Morbidity Mortality Weekly Rep. 1976.

177. Tewdros Eguale, Getahun Abate, Balcha F. Relapsing Fever in Hossana, Ethiopia: A Clinical and Epidemiological Study. Ethiopian Journal of Health Sciences. 2002;12(2):103-8.

178. Legesse MW, Gebre-Selassie S. Louse-Borne Relapsing Fever Profile at Jimma Hospital, Ethiopia: a retrospective study. Ethiopian Journal of Education and Sciences. 2005;1(1):60-4. doi: 10.4314/ejesc.v1i1.41987.

179. Ludlow AI. Relapsing fever in Korea. Ohio State Medical Journal. 1943;39:1011-3.

180. Nordmann T, Feldt T, Bosselmann M, Tufa TB, Lemma G, Holtfreter M, et al. Outbreak of Louse-Borne Relapsing Fever among Urban Dwellers in Arsi Zone, Central Ethiopia, from July to November 2016. American Journal of Tropical Medicine and Hygiene. 2018;98(6):1599-602. doi: 10.4269/ajtmh.17-0470. PubMed PMID: BIOSIS:PREV201800626499.

181. Alberer M, Malinowski S, Sanftenberg L, Schelling J. Notifiable infectious diseases in refugees and asylum seekers: experience from a major reception center in Munich, Germany. Infection. 2018;46(3):375-83. doi: 10.1007/s15010-018-1134-4.

182. Fasciana T, Calà C, Colomba C, Mascarella C, Scarlata F, Capra G, et al. A new case of louse-borne relapsing fever in sicily: Case report and mini review. Pharmacologyonline. 2017;1(Special Issue):62-6.

183. Antinori S, Tonello C, Edouard S, Parravicini C, Gastaldi D, Grande R, et al. Diagnosis of louse-borne relapsing fever despite negative microscopy in two asylum seekers from Eastern Africa. American Journal of Tropical Medicine and Hygiene. 2017;97(6):1669-72. doi: 10.4269/ajtmh.17-0320.

184. Daniel E, Beyene H, Tessema T. Relapsing fever in children--demographic, social and clinical features. Ethiopian Medical Journal. 1992;30(4):207-14. PubMed PMID: 1459120.
