# Supplementary figures and images for: Louse-borne relapsing fever—A systematic review and analysis of the literature: Part 2—Mortality, Jarisch–Herxheimer reaction, impact on pregnancy
Source: PLoS Negl Trop Dis. 2021 Mar 11;15(3):e0008656. doi: 10.1371/journal.pntd.0008656 (PMC7951929; doi:10.1371/journal.pntd.0008656)

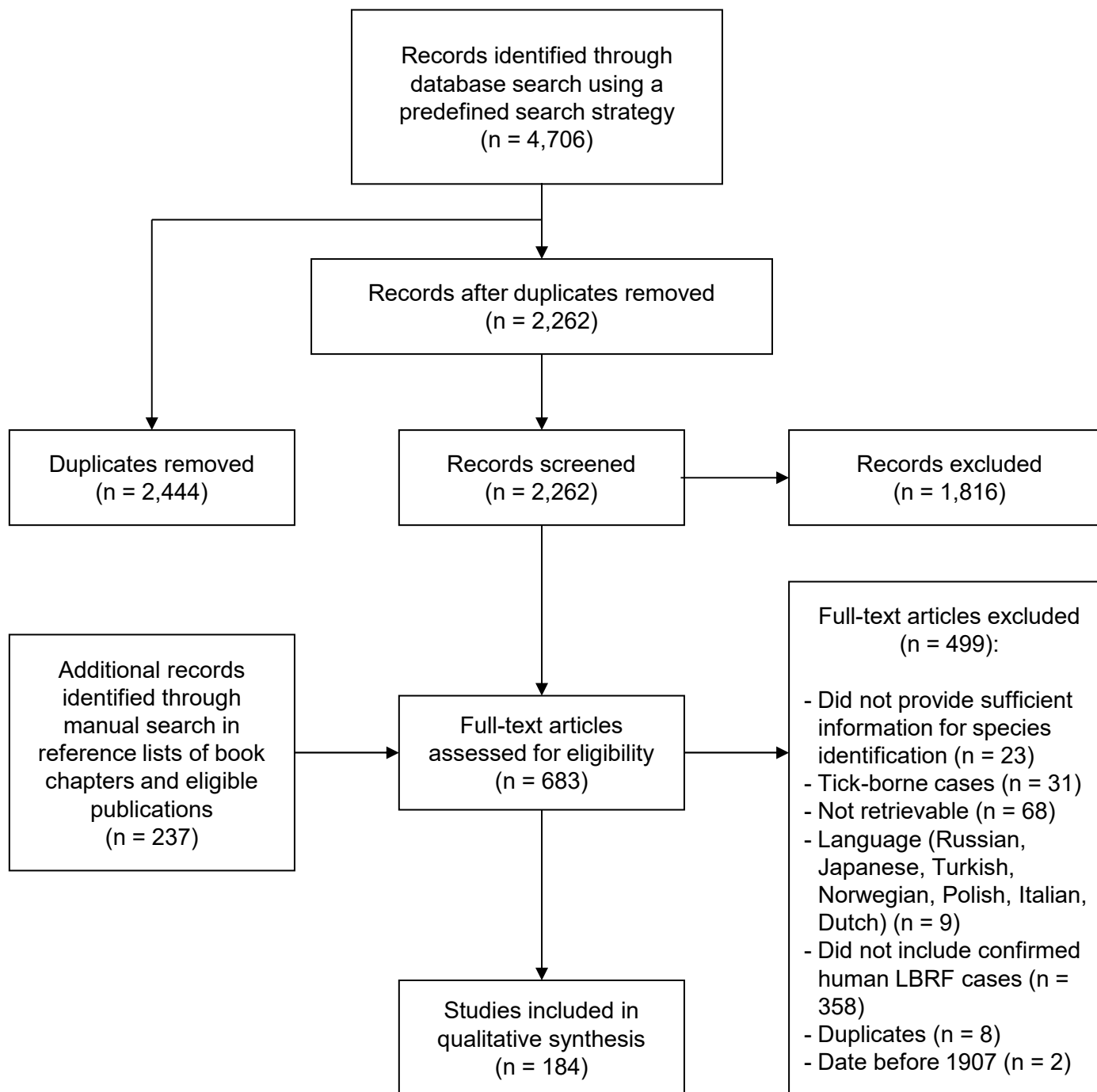

Supplement: S1 Fig — (PDF) [file pntd.0008656.s008.pdf]
